# Effective Harmonic Potentials: Insights into the Internal Cooperativity and Sequence-Specificity of Protein Dynamics

**DOI:** 10.1371/journal.pcbi.1003209

**Published:** 2013-08-29

**Authors:** Yves Dehouck, Alexander S. Mikhailov

**Affiliations:** 1 Department of Physical Chemistry, Fritz-Haber-Institut der Max-Planck-Gesellschaft, Berlin, Germany; 2 Department of BioModelling, BioInformatics and BioProcesses, Université Libre de Bruxelles (ULB), Brussels, Belgium; Fox Chase Cancer Center, United States of America

## Abstract

The proper biological functioning of proteins often relies on the occurrence of coordinated fluctuations around their native structure, or on their ability to perform wider and sometimes highly elaborated motions. Hence, there is considerable interest in the definition of accurate coarse-grained descriptions of protein dynamics, as an alternative to more computationally expensive approaches. In particular, the elastic network model, in which residue motions are subjected to pairwise harmonic potentials, is known to capture essential aspects of conformational dynamics in proteins, but has so far remained mostly phenomenological, and unable to account for the chemical specificities of amino acids. We propose, for the first time, a method to derive residue- and distance-specific effective harmonic potentials from the statistical analysis of an extensive dataset of NMR conformational ensembles. These potentials constitute dynamical counterparts to the mean-force statistical potentials commonly used for static analyses of protein structures. In the context of the elastic network model, they yield a strongly improved description of the cooperative aspects of residue motions, and give the opportunity to systematically explore the influence of sequence details on protein dynamics.

## Author Summary

Decades of experimental evidence have underlined the fact that protein structures can hardly be considered as static objects. To understand how a protein achieves its biological purpose, it is therefore quite often necessary to unravel the complexity of its dynamical behavior. However, the definition of accurate and computationally tractable descriptions of protein dynamics remains a highly challenging task. Indeed, even though proteins are all built from a limited set of amino acids and local conformational arrangements, the specific nature of biologically relevant motions may vary widely from one protein to another, which constitutes a serious obstacle to the identification of common rules and properties. Here, instead of focusing on the study of a single protein, we adopt a more general perspective by condensing the information contained in a multitude of NMR conformational ensembles. This approach allows us to characterize the dynamical behavior of residues and residue pairs in a mean protein environment, independently of each protein's specific architecture. We describe how this analysis can be exploited to assess the performances of coarse-grained models of protein dynamics, to take advantage of existing experimental data for a more rational and efficient parametrization of these models and, ultimately, to improve our understanding of the intrinsic dynamical properties of amino acid chains.

## Introduction

Deciphering the motions that underlie many aspects of protein function is a major current challenge in molecular biology, with the potential to generate numerous applications in biomedical research and biotechnology. Although molecular dynamics (MD) hold a prominent position among computational approaches, considerable efforts have been devoted to the development of coarse-grained models of protein dynamics [1]. Besides their ability to follow motions on time scales that are usually not accessible to MD simulations, these models also give the possibility to better understand the general principles that rule the dynamical properties of proteins.

The elegant simplicity of the elastic network models (ENM) certainly contributed to their popularity, and they have been successfully exploited in a wide range of applications [2]–[5]. In these models, the residues are usually represented as single particles and connected to their neighbors by Hookean springs [6], [7]. The input structure is assumed to be the equilibrium state, i.e. the global energy minimum of the system. Common variants include the homogeneous ENM, in which springs of equal stiffness connect pairs of residues separated by a distance smaller than a predefined cutoff, and other versions in which the spring stiffness decays as the interresidue distance increases [8]–[10]. In all cases, the equations of motion can be either linearized around equilibrium, to perform a normal mode analysis of the system [11]–[13], or integrated to obtain time-resolved relaxation trajectories [14], [15].

Despite their many achievements, purely structural ENM also come with severe limitations. Notably, modeling the possible effects of mutations within this framework usually requires random local perturbations of the spring constants [16], or a more drastic removal of links from the network [17]. A few attempts have been made to include sequence-specificity in the ENM by setting the spring constants proportional to the depth of the energy minima, as estimated by statistical contact potentials [18], [19]. However, this approach cannot be extended to distance-dependent potentials, for they are not consistent with the ground hypothesis of the ENM, i.e. that all pairwise interaction potentials are at their minimum in the native structure. Other studies have led to the conclusion that the ENM behave as entropic models dominated by structural features, and that the level of coarse-graining is probably too high to incorporate sequence details [7], [20]. Still, the chemical nature of residues at key positions can have significant effects on the main dynamical properties of a protein. Hinge motions [21], for instance, obviously require some architectural conditions to be fulfilled, such as the presence of two domains capable of moving relatively independently. But the amplitude and preferred direction of the motion are most likely determined by fine tuning of specific interactions in the hinge region. In proteins subject to domain swapping, the hinge loops have indeed been shown to frequently include residues that are not optimal for stability [22]. The importance of the amino acid sequence has also been repeatedly emphasized by experimental studies of the impact of mutations on the conformational dynamics of proteins [23]–[25].

A major obstacle to the definition of accurate coarse-grained descriptions of protein dynamics lies in the highly cooperative nature of protein motions, which makes it difficult to identify the properties of the individual building blocks independently of the overall architecture of each fold. By condensing the information contained in a multitude of NMR ensembles, we build here a mean protein environment, in which the behavior of residue pairs can be tracked independently of each protein's specific structure. This methodology brings an efficient way of assessing coarse-grained models of protein dynamics and of deriving effective energy functions adapted to these models. In the context of the ENM, we identify a set of spring constants that depend on both the interresidue distances and the chemical nature of amino acids, and that markedly improve the performances of the model.

## Results

### Dynamical properties of proteins from the perspective of an average pair of residues

The mean-square fluctuations of individual residues (MSRF) have been extensively relied on to characterize protein flexibility and to evaluate coarse-grained models of protein dynamics [26], in part because of their widespread availability as crystallographic B-factors. However, since the MSRF carry little information about the cooperative and anisotropic nature of residue motions, we propose to examine the dynamical behavior of proteins from the perspective of residue pairs rather than individual residues. Information about the fluctuations of interresidue distances is contained in the data of NMR experiments for numerous proteins, and will be exploited here. We define the apparent stiffness of a pair of residues 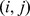 in a protein 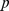:
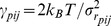
(1)where 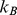 is the Boltzmann constant, 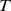 the temperature, and 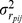 the variance of the distance 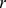 between residues 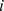 and 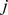, in a structural ensemble representative of the equilibrium state. 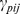 is defined up to a multiplicative factor, which corresponds to the temperature. We also introduce the uncorrelated apparent stiffness 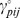, to quantify the impact of the individual fluctuations of residues 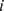 and 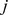 on the fluctuations of the distance that separates them. This is achieved by using 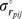 instead of 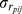 in eq. **1**, where 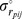 is computed after exclusion of all correlations between the motions of residues 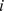 and 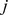 (see Methods).

**Figure 1:**
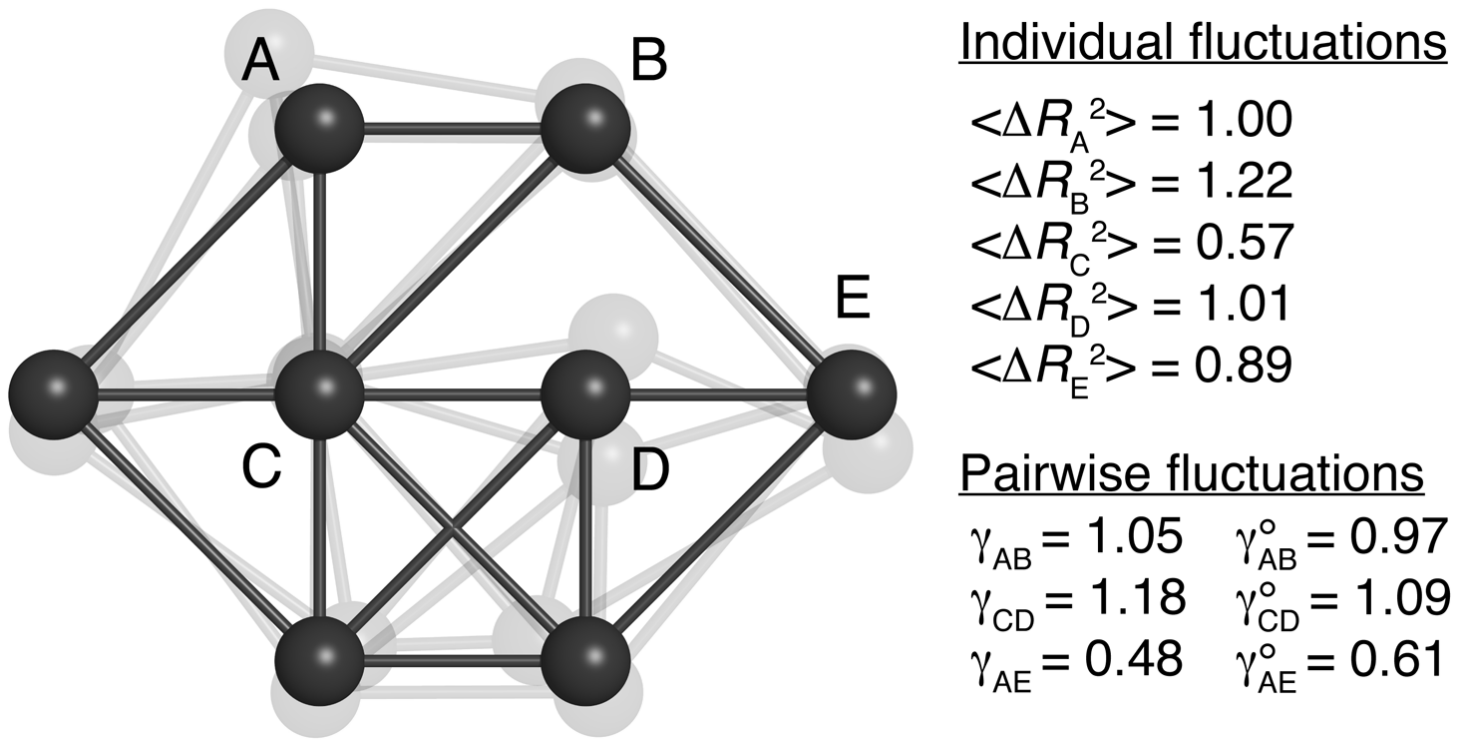
Schematic illustration of the apparent stiffness 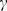. A simple model containing 8 beads connected by elastic springs was subjected to 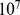 integration steps under Gaussian noise. Selected values of 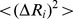, 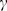 and 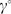 are given in arbitrary units. Individually, the pairs A–B and C–D would be identical, but they experience differently the influence of the other beads. As a result, the C–D pair is effectively more rigid than A–B (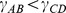). In both cases, the motions are somewhat correlated, as the apparent stiffness 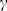 is larger than what is expected from the knowledge of their individual motions (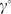). Beads A and E do not interact directly but the effect of the network on their relative motions is captured by the values of 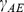 and 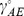.

As illustrated in Figure 1, 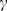 can be quite different from one residue pair to another. Indeed, besides the impact of direct interactions, 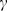 is also strongly dependent on the overall fold of the protein, and on the position of the pair within the structure. To remove the specific influence of each protein's architecture, we define the apparent stiffness in a mean protein environment 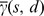:
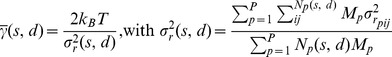
(2)where 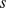 is one of 210 amino acid pairs, 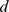 the discretized equilibrium distance between pairs of residues (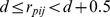
Å), 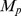 the number of structures in the equilibrium ensemble of protein 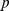, and 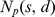 the number of 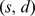 residue pairs in protein 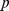. Pairs of consecutive residues were dismissed, so as to consider only non-bonded interactions. The mean protein environment is thus obtained by averaging over a large number of residue pairs in a dataset of 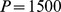 different proteins (see Methods).

**Figure 2:**
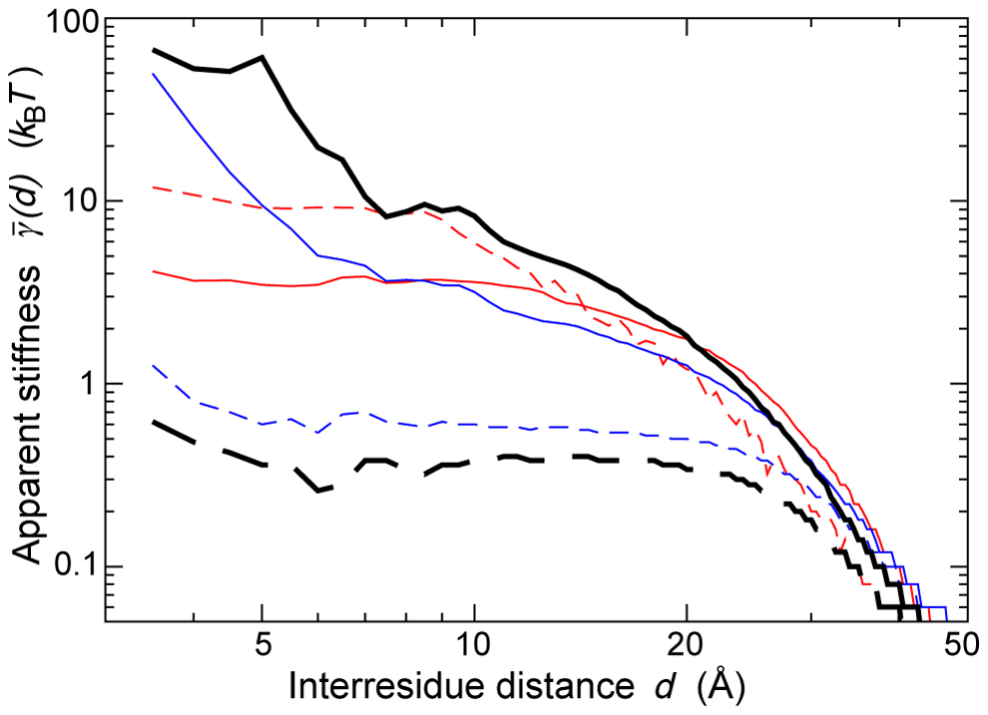
Comparison of the experimental and predicted values of the apparent stiffness 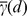. Experimental values of 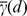 (continuous black) and 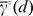 (dashed black), extracted from the dataset of 1500 NMR ensembles. Values of 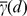 predicted on the same dataset by the 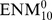 (dashed red); 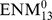 (continuous red); 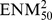 (dashed blue); 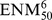 (continuous blue).

The influence of the distance separating two residues on the cooperativity of their motions can be investigated by considering amino acid types indistinctively in eq. **2**. Interestingly, 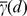 follows approximately a power law, with an exponent of about −2.5 (Figure 2). Finer details include a first maximal value occurring for 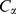
–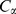 distances between 5 and 5.5 Å, i.e. the separation between hydrogen-bonded residues within regular secondary structure elements, and a second around 9 Å, which corresponds to indirect, second neighbor, interactions. The high level of cooperativity in residue motions is well illustrated by the comparison of 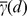 and its uncorrelated counterpart 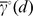. Indeed, these two functions would take identical values if the variability of the distance between two residues could be explained solely by the extent of their individual fluctuations. In a mean protein environment, however, 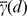 is about two orders of magnitude larger than 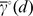 at short-range, and the difference remains quite important up to about 30–40 Å.

The comparison of 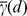 values extracted from subsets containing exclusively small, large, all-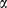, or all-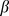 proteins indicates that the content of the dataset has a remarkably limited impact on 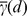 (Figure S1). This distance dependence can thus be seen as a general property of protein structures, a signature of protein cooperativity at the residue pair level. Of course, since 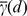 is representative of a mean protein environment, deviations may occur for individual proteins, according to their specific structural organizations (Figure S2).

**Figure 3:**
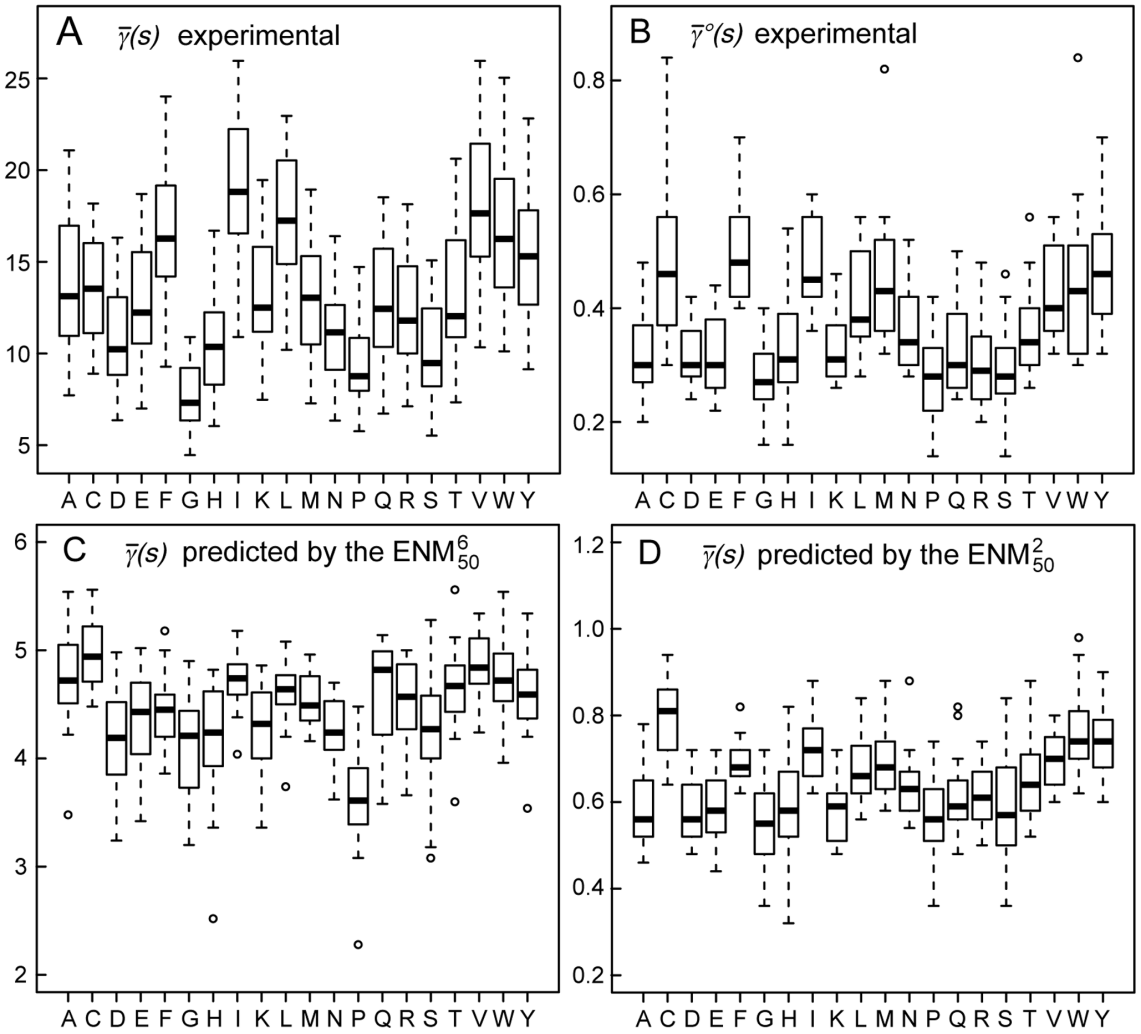
Comparison of the experimental and predicted values of the apparent stiffness 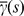. For each amino acid, the median value of 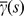 over the 20 possible partners is given in units of 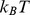, along with the maximal, minimal, 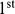 and 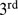 quartile values. Outliers from these distributions are depicted as circles. (A) Experimental values of 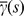, extracted from the dataset of 1500 NMR ensembles. (B) Experimental values of 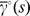, extracted from the same dataset. (C) Values of 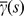 predicted by the 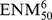, on the same dataset. (D) Values of 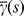 predicted by the 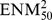, on the same dataset.

The apparent stiffness 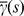 is computed for each type of amino acid pair 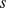 using eq. **2**, by considering only residue pairs separated by less than 10 Å. As shown in Figure 3A, the chemical nature of the interacting residues is a major determinant of their dynamical behavior. Unsurprisingly, Glycine and Proline appear as the most effective ingredients of flexibility. Pairs involving hydrophobic and aromatic amino acids tend to be considerably more rigid, with 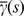 values up to 6 times larger. These differences originate in part in the individual propensities of different amino acids to be located in more or less flexible regions (e.g. hydrophobic core vs. exposed surface loops). However, there is only a limited agreement between 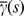 and 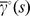 (Figure 3A–B): the correlation coefficient is equal to 0.71, and 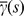 spans a much wider range of values. Beyond individual amino acid preferences, the specifics of residue-residue interactions play thus a significant role in determining the extent of cooperativity in residue motions.

### Accuracy of elastic network models in reproducing the dynamical properties of proteins

The computation of the apparent stiffness of residue pairs in a mean protein environment provides an interesting tool to probe the dynamical properties of proteins. It also generates a very straightforward approach to assess the ability of coarse-grained models to reproduce accurately this general behavior.

We focus here on four common variants of the residue-based ENM [27], [28], which differ only by the functional form of the spring constants 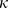. The dependence of 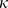 on the interresidue distance 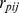 is defined by two parameters: the cutoff distance 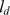, above which residues 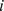 and 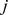 are considered disconnected, and the exponent 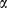 that determines how fast 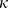 decreases with increasing distances:
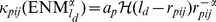
(3)where 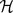 is the Heaviside function. The value of the temperature-related factor 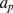 is obtained, for each protein independently, by fitting the predicted MSRF with the experimental ones. This ensures that the amplitude of the individual fluctuations of the beads in the network is on average equal to that observed in the corresponding NMR ensemble, and that the predicted 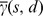 values can thus be directly compared with those extracted from the NMR data. We consider the following models: 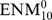, 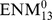, 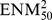, 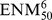. These ENM variants were used to estimate the value of 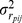 for each pair of residues in the 1500 proteins of our NMR dataset (see Methods), and to subsequently compute 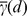 and 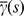 from eq. **2**.

Strikingly, all ENM variants systematically predict 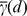 values to be lower than the experimental ones, at least up to interresidue distances of 20–30 Å (Figure 2). These models overestimate thus the amplitude of pairwise fluctuations, relatively to the amplitude of individual fluctuations. For example, if two residues in a protein undergo highly correlated motions, the amount of thermal energy necessary to induce a moderate variance on the distance between them will generate high variances on their individual coordinates. Consequently, if the motions of the beads of the ENM are less coordinated, adjusting the scale of the spring constants to reproduce the amplitude of individual fluctuations leads to an overestimated variance on the interresidue distances, and thus to lower 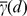 values. This problem is particularly apparent when 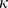 is assumed to decrease proportionally to the square of the interresidue distance, in the 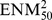. Although this model was shown to perform well in predicting MSRF values [10], our results suggest that it negates almost completely the coordinated aspect of residue motions. Indeed, as shown in Figure 2, the 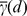 values predicted by this model are very close to those obtained from the experimental data after removal of the correlations between the motions of the different residues (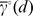). This observation is consistent with the extremely short atom-atom correlation length characteristic of the 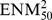, recently estimated on the basis of an X-ray structure of Staphylococcal nuclease [27].

The ENM is often considered as an entropic model, not detailed enough to include sequence information in a relevant way [7], [20]. It is therefore hardly surprising that common ENM variants produce a poor description of the sequence specificities of protein dynamics. Individual amino acid preferences for more or less densely connected regions are responsible for some variety in the predicted values of 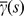 (Figure 3C–D). However, this variety is far from matching the one observed in the experimental data, as shown by a much narrower range of 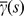 values, and a limited correlation coefficient with the experimental 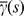 values, e.g. 0.64 for the 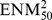 and 0.62 for the 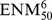 (Figure S3). There is a much better agreement between the 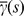 values predicted by the 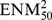, and the experimental values of the uncorrelated apparent stiffness 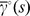 (Figure 3B,D, correlation coefficient of 0.84), which confirms that this model ignores the coordinated aspects of residue motions.

### Derivation of effective harmonic potentials

Mean-force statistical potentials are commonly used to perform energetic evaluations of static protein structures [29]–[31]. These potentials do not describe explicitly the “true” physical interactions, but provide effective energies of interaction in a mean protein environment, in the context of a more or less simplified structural representation. Similarly, within the ENM framework, 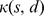 defines for each pair of residues an harmonic interaction potential. This potential is also effective in nature, accounting implicitly for everything that is not included in the model (e.g. the surrounding water). Hence, we seek to identify the value of 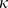 yielding the most accurate reproduction of the dynamical behavior of each type of pair 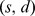 in a mean protein environment, which is conveniently captured by the apparent stiffness 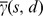.

For that purpose, let us define 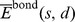 as the energy of the elastic spring connecting two residues of type 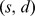, in a mean protein environment:
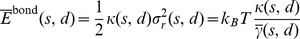
(4)where 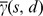 is the apparent stiffness extracted from the experimental data. 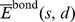 is unknown and is expected to be different for different pair types 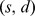. The knowledge of 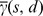 is thus not sufficient to estimate directly 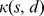. However, from any approximate set of spring constants 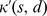, we may build the ENM for all proteins in our dataset, to reproduce the mean protein environment, and compute for each pair type an estimated value of the apparent stiffness, 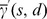, and bond energy, 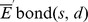.

Since the behavior of a given residue pair is highly dependent on its environment, we can make the assumption that 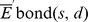 is a relatively good approximation of 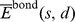, even if 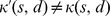:
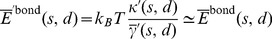
(5)


Indeed, if the spring stiffness of a residue pair is underestimated 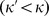, it will also appear as less rigid in the ENM than in the experimental data 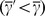. A more detailed discussion is given in Supporting Text S1. From eqs. **4** and **5**, we devise thus an iterative procedure in which 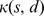 is updated at each step 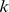 by confronting the predicted values of the apparent stiffness, 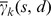, with the experimental ones, 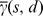. It is expected to converge when 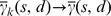, that is, when the predictions of the model agree with the experimental data:
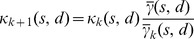
(6)


We used this approach to derive, from the NMR data, four novel ENM variants: the distance-dependent dENM ; the sequence-dependent 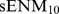 and 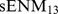, with a distance cutoff of 10 and 13 Å, respectively, and the sequence- and distance-dependent sdENM (see Methods).

**Figure 4:**
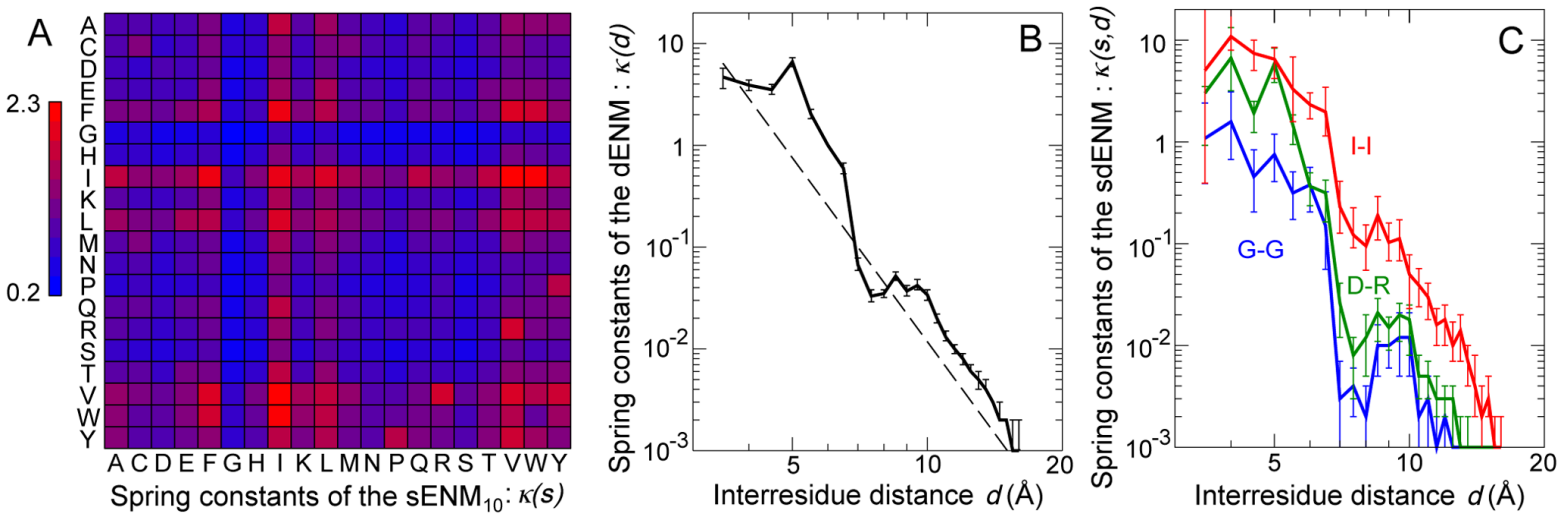
Effective harmonic potentials. (A) Spring constants of the 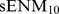, for the 210 amino acid pairs. (B) Spring constants of the dENM. The dashed line corresponds to 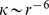. (C) Spring constants of the sdENM for 3 amino-acid pairs. The error bars in panels B–C correspond to the bootstrap estimates of the 90% confidence intervals (see Methods). All 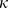 values are given in Tables S2, S3, S4, S5, and in Dataset S1.

Interestingly, the 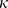 values for the 210 amino acid pairs in the 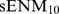 are relatively well correlated with the corresponding contact potentials [30], even though they result from totally different approaches (Figure S4). Some common general trends can be identified, e.g. hydrophobic contacts tend to be associated with both favorable interaction energies and large 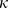 values (Figure 4A). However, the overall correspondence remains limited, indicating that the determinants of protein rigidity and stability are related, but distinct.

The distance dependence of 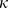 in the dENM is remarkably similar to the 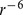 power law that was previously obtained by fitting against a 1.5 ns MD trajectory of a C-phycocyanin dimer [8] (Figure 4B), although our new model presents more detailed features. Notably, 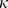 remains approximately constant up to interresidue distances of 5–6 Å, and then drops by about two orders of magnitude to reach a second plateau between 7 and 12 Å. The bootstrap estimates of the 90% confidence intervals displayed on Figure 4B underline the robustness of our derivation scheme, and indicate that the 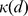 values determined here depend only marginally on the content of the dataset.

The 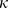 values of the sdENM are shown in Figure 4C, for a few amino acid pairs. This model not only combines the strengths of the sENM and the dENM, but also reveals the sequence specificity of the 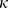 distance dependence. The D-R pair, for example, is almost as rigid as I-I at short distances consistent with the formation of a salt bridge, but almost as flexible as G-G at larger distances. There is of course a larger uncertainty on the determination of 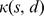 values, which is reflected by wider confidence intervals than those on 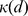 in the dENM (Figure 4B,C). This is due to the limited amount of available experimental data, and to the fact that the modelled dynamical behavior of a protein is obviously less sensitive to variations of the spring constant values for one type of amino acid pair, than for all amino acid pairs indistinctively. However, this uncertainty remains small enough to allow the identification of significant differences between the 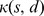 values determined for different types of amino acid pairs. In the example of Figure 4C, 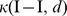 is consistently larger than 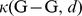 over the whole range of inter-residue distances, whereas 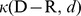 is significantly larger than 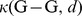 at short-range (4–6 Å), and significantly smaller than 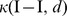 at mid-range (6–12 Å).

### Performances of the new ENM

**Figure 5:**
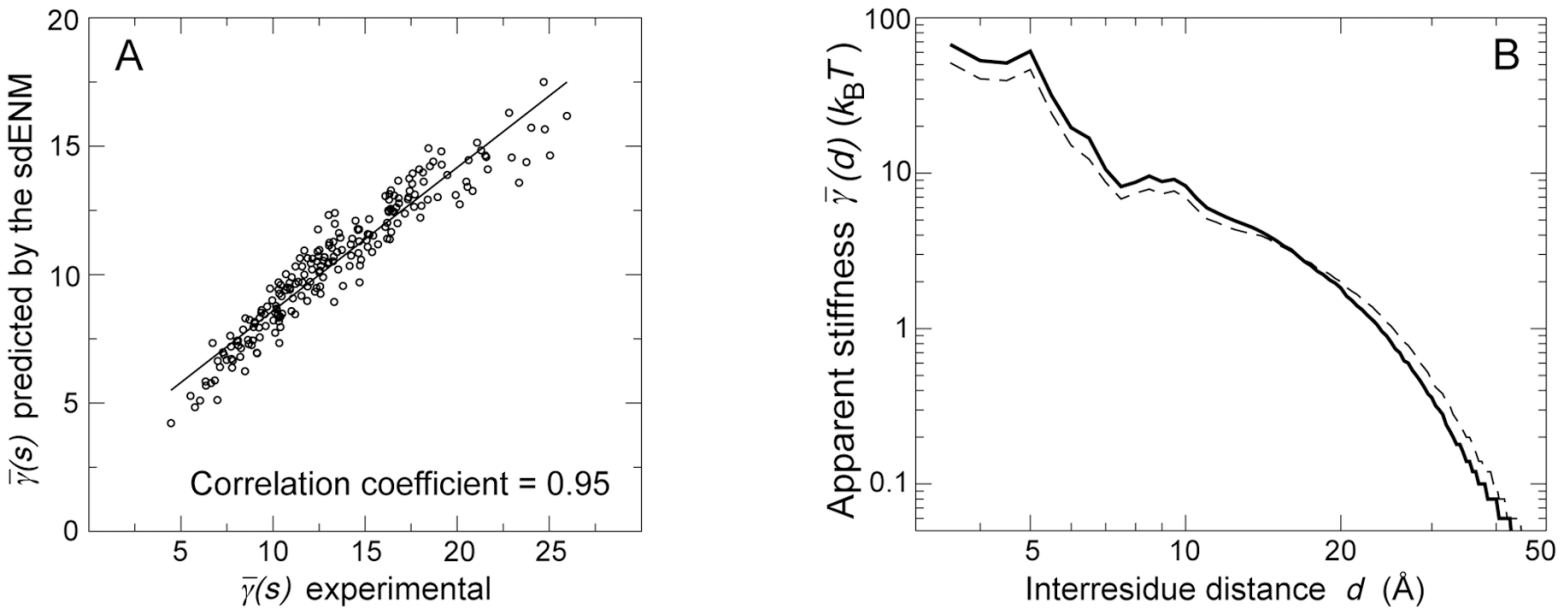
Performances of the sdENM in a mean protein environment. (A) Experimental and predicted values of 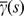, in the dataset of 1500 NMR ensembles. The Pearson correlation coefficient between predictions and experimental data is equal to 0.95 (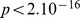). See also Figures S3 and S5. (B) Experimental (continuous) and predicted (dashed) values of 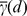, in the dataset of 1500 NMR ensembles. See also Figure S2.

The sdENM yields a much more accurate reproduction of the dynamical behavior of residue pairs in a mean protein environment than the common ENM variants, as demonstrated by the good agreement between experimental and predicted values of 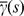 (Figures 5A, S5), and 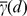 (Figure 5B).

**Figure 6:**
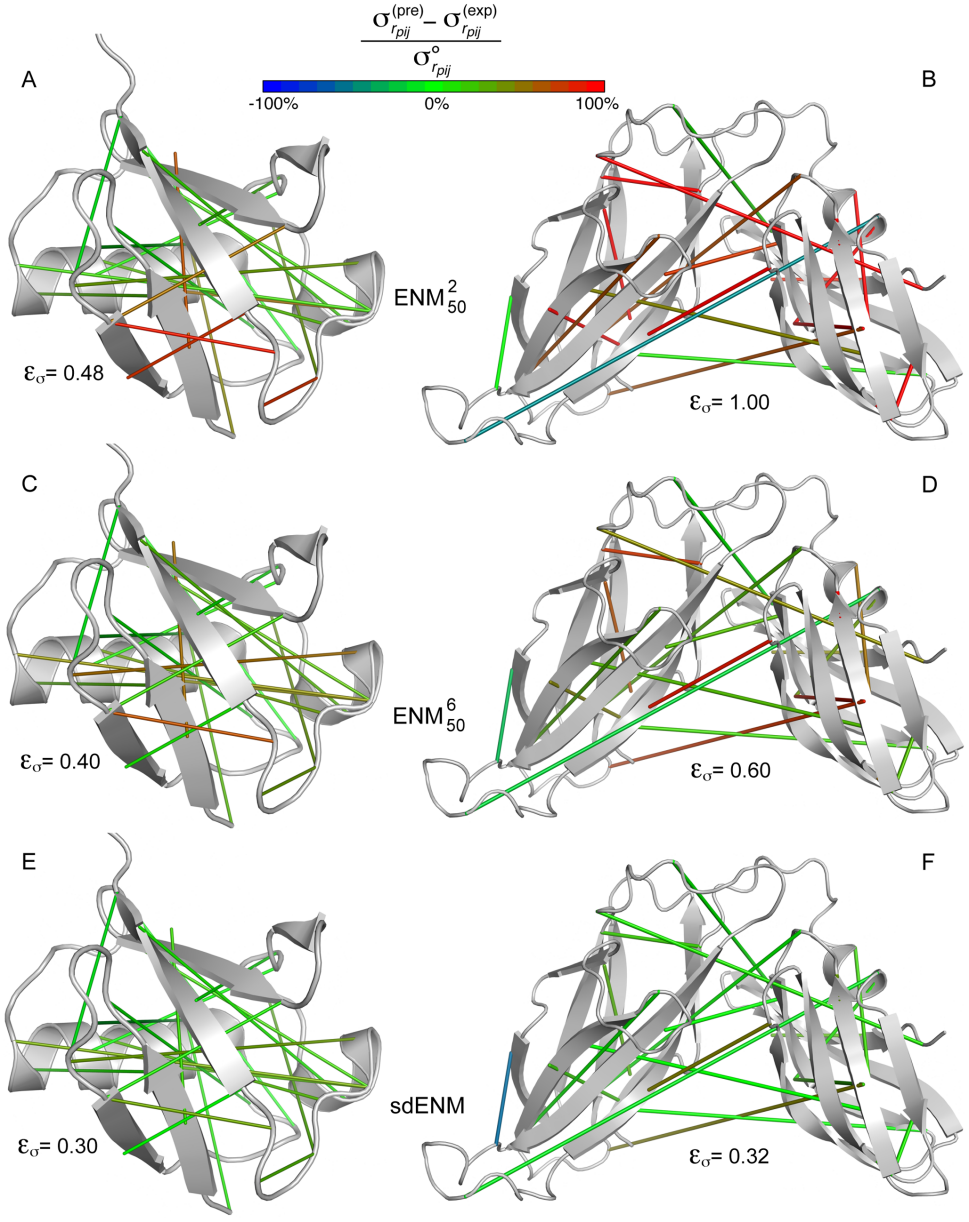
Performances of the sdENM on individual proteins. The accuracy of the estimation of pairwise residue fluctuations by different ENM variants is illustrated on the basis of two individual proteins. For each protein, 20 randomly selected residue pairs (10 with 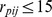
Å, and 10 with 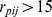
Å) are connected by solid lines. A green line indicates that the amplitude of the fluctuations of the interresidue distance is well estimated by the model. A red (blue) line indicates that the amplitude of the fluctuations of the interresidue distance is largely overestimated (underestimated) by the model. Values larger than 100% or lower than −100% are assimilated to 100% and −100%, respectively. In addition, for each protein and each ENM variant, we report the error 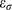 on the estimation of pairwise fluctuations (eq. 15), which accounts for all pairs of residues in the protein. (A,C,E) High quality structural ensemble of ubiquitin, obtained by combining NMR information with molecular dynamics simulations (PDB: 1xqq) [38]. (B,D,F) NMR structural ensemble of periplasmic chaperone FimC (PDB: 1bf8). The relatively rigid orientation of the two domains is ensured by specific interdomain interactions [39]. (A–B) 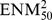. (C–D) 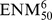. (E–F) 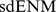.

Beyond its performances in a mean protein environment, our new model also brings highly notable improvements with respect to previously described ENM variants when it is applied to the specific architecture of a given protein. This is illustrated by two examples, on Figure 6. A more thorough assessment of the ability of the different ENM variants to capture the motions of individual proteins was performed on an independent dataset of 349 proteins. The correlation coefficient between predicted and observed MSRF (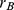) has been widely used in the past but ignores the cooperativity inherent to protein dynamics, and presents other shortcomings. Therefore, we introduce a new measure (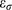) that quantifies the relative error on the estimation of the variability of the distance between residue pairs, and is thus focused on the cooperative aspects of residue motions (see Methods).

Among the 4 previously described ENM variants, the 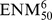 is better at predicting the individual residue fluctuations (Table 1). Interestingly, the 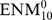, with its simple cutoff distance, appears superior when it comes to the reproduction of cooperative motions (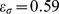). The new ENM variants based on our effective harmonic potentials present enhanced performances in comparison with the common models. In particular, the dENM reaches the same level of quality as the 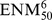 for individual fluctuations (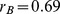), but surpasses even the 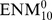 for the description of cooperativity (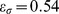). On the other hand, the impact of introducing sequence specificity can be examined by comparing 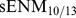 with 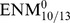, and sdENM with dENM. It consists in a slight improvement of the correlation coefficient 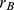, and a pronounced decrease of the error 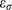, especially at short- (0–15 Å) and mid- (15–30 Å) range.

**Table 1:** Performances of different ENM variants.

	*r_B_* (a)	*ε_σ_* (b)	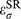	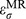	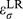
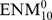	0.63	0.59	0.53	0.59	0.68
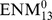	0.65	0.68	0.69	0.68	0.68
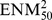	0.66	0.97	1.07	0.96	0.74
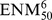	0.69	0.64	0.59	0.66	0.66
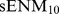	0.63	0.55	0.49	0.55	0.67
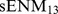	0.66	0.63	0.63	0.63	0.67
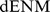	0.69	0.54	0.48	0.56	0.60
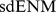	0.70	0.48	0.41	0.49	0.57

(a) Average correlation coefficient between experimental and measured MSRF.

(b) Average relative error on the fluctuations of interresidue distances.

## Discussion

For the last decades, statistical potentials extracted from datasets of known protein structures [29]–[31] have played a critical role in static analyses of protein structures, with major applications including structure prediction, protein-protein docking, or rational mutant design. Our study demonstrates that a similar approach can be taken to derive effective energy functions that are specifically adapted to the coarse-grained modeling of protein dynamics.

More precisely, in the context of the ENM, we exploited a dataset of 1500 NMR ensembles to determine the values of the spring constants that describe best the behavior of pairs of residues, as a function of both their chemical nature and the distance separating them. The success of our approach is attested by a drastic enhancement of the ability to accurately reproduce the cooperative nature of residue motions, with respect to previously described ENM variants. Moreover, a definite advantage of our method is that the effective parameters characterizing the strength of the virtual bonds are directly extracted from the experimental data without any a priori conception of their functional form. The fact that the distance dependence of the spring constants that we retrieve is quite similar to the 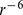 power law, which was considered so far as underlying one of the best performing ENM variants [8], [27], also constitutes a major support to our approach.

In our derivation scheme, the virtual bonds are parametrized so as to reproduce the behavior of amino acid pairs in a mean protein environment. The analysis of the ability of different models of protein dynamics to describe the motions of residues within this environment sheds an interesting new light on the properties of these models. In particular, our results indicate that previous ENM variants underestimate, sometimes dramatically, the rigidity of amino acid pairs at short- and mid-range. Our new model does however provide a much more accurate reproduction of the balance between short-range and long-range coordinated motions. This is arguably a crucial aspect when considering, for example, the consequences of localized alterations induced by ligand binding on signal transduction or global conformational changes, such as in ATP-powered molecular motors.

Importantly, our results also demonstrate that the ENM does not have to be exclusively structural, and that sequence details may be allowed to play a major role in coarse-grained descriptions of protein dynamics. Thereby, this study paves the way towards comparative analyses of motions in proteins that share a similar structure but present differences in sequence. Such investigations will prove particularly interesting in the context of the rational design of (modified) proteins with controlled dynamical properties. On the other hand, the importance of orientational effects in protein dynamics has been underlined by both experimental and computational studies [5], [7], [32]–[36]. At the protein level, these effects are nicely illustrated by the strong anisotropy of a protein's response to applied external forces [33], [34], [36]. At the residue level, the anisotropy can be related to the directional variability of the packing density experienced by any given residue [5], [35]. The accurate description of such orientational effects should benefit from the availability of sequence-specific models. Indeed, beyond the number of contacts established in each direction, the actual nature of these contacts can also have a substantial influence on the anisotropy of residue fluctuations. Although we focused here on residue-based elastic network models, our approach is not limited to this particular family, and can be readily implemented to use available dynamical data for the evaluation and optimization of other coarse-grained models of protein dynamics. Notably, the impact of chemical specificity on the dynamical behavior of residues should be even more accurately rendered by effective potentials based on a more detailed structural description.

## Methods

### NMR dataset

We retrieved, from the Protein Data Bank [37], ensembles of at least 20 models from solution NMR experiments, corresponding to monomeric proteins of at least 50 residues that present at most 30% sequence identity with one another. Entries under the SCOP classifications “Peptides” or “Membrane and cell surface proteins” were not considered. The presence of ligands, DNA or RNA molecules, chain breaks, non-natural amino acids, and differences in the number of residues per model were also grounds for rejection. These criteria led to the selection of 1849 distinct structural ensembles. A subset of 1500 ensembles was randomly selected for the main analysis, and the remaining 349 were used to assess the performances of the different ENM variants. Unfolded C- or N-terminal tails were automatically identified (MSRF values larger than twice the average for all residues in the protein) and removed from consideration. In each ensemble, the structure with the lowest root mean square deviation from the mean structure, after superposition, is chosen as representative and used to build the ENM.

### Elastic network model

The network is built by considering each residue as a single bead, placed at the position of the corresponding 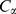 atom in the input structure, and connecting neighboring beads with Hookean springs [6], . The ENM variants considered here differ only by the form of the spring constant 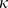 as a function of interresidue distance and of amino acid types. In all variants, bonded interactions are described by a larger value of 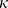, defined as ten times the value of 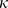 for non-bonded interactions at a separation of 3.5 Å, averaged over all amino acid types. The potential energy of the network is given by: 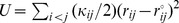, where 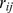 and 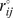 are the instantaneous and equilibrium distances between residues 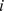 and 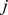, respectively. By definition, the input structure corresponds to the global energy minimum, with 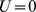. For a protein of 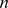 residues, the Hessian 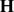 of the system is the 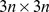 matrix of the second derivatives of 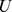 with respect to the spatial coordinates of the residues. The eigenvalue decomposition of 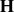 yields the covariance matrix 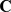 of the spatial coordinates, which constitutes the output of the model:
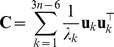
(7)where the sum is performed over the 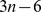 non-zero eigenvalues 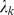 of 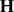, and 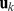 are the corresponding eigenvectors. 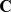 is a 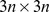 symmetrical matrix, constituted of 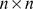 submatrices 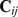:
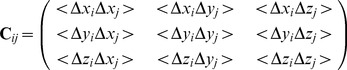
(8)where 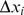, 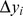, and 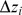 correspond to the displacements of residue 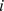 from its equilibrium position, along the three Cartesian coordinates. The predicted MSRF of residue 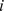 is given by the trace of submatrix 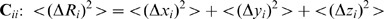.

### Variance of the interresidue distance

For each pair of residues in a given protein 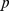, the experimental value of this variance is readily computed from the NMR data:
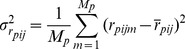
(9)where 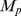 is the number of structures in the NMR ensemble, 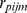 the distance between the 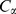 atoms of residues 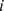 and 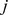 in structure 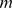 of protein 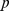, and 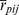 the average distance over all 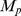 structures. In the context of the ENM, 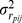 values are estimated from the covariance matrix of the spatial coordinates, by standard statistical propagation of uncertainty:
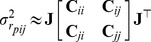
(10)where 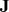 is the Jacobian of the distance 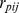 as a function of the six spatial coordinates:
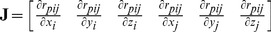
(11)This estimation of 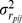 takes into account the individual, anisotropic, fluctuations of both residues, as well as the correlations between their respective motions. It relies on the validity of the first order Taylor expansion of the distance as function of the coordinates in the vicinity of the average distance. We ensured that no systematic bias arose from this approximation (Figure S6).

To quantify the impact of the individual motions of residues on their relative positions, we use eq. **10** to compute 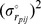 in an artificial construct where residue motions are not correlated. This is achieved by extracting the covariance matrix from the NMR data, and setting to zero all submatrices 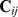 where 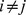.

### Iterative procedure

The values of the spring constants of the new ENM variants were derived from the dataset of 1500 NMR ensembles using eq **6**. For the dENM, 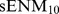 and 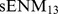, the initial values of the spring constants were set equal to the experimental values of the apparent stiffness: 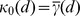 or 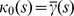. Note that the 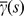 values were computed by considering only residue pairs separated by a distance lower than the cutoff of 10 or 13 Å. For the sdENM, the 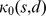 values were set equal to the final values of the spring constants in the dENM, 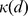, for all amino acid types.

A correction for sparse data was devised to ensure that 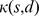 tends to 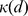 when the number of residue pairs of type 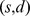 is too small to obtain relevant estimations of 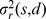. Instead of eq. **2**, we used the following definition to compute both the experimental and predicted apparent stiffness:
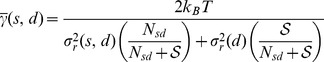
(12)where 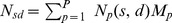, 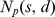 is the number of pairs of type 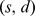 in protein 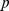, and 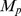 is the number of structures in the NMR ensemble of protein 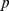. The adjustable parameter 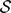 can be understood as the number of occurrences of a 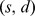 residue pair, 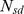, that is needed to obtain a relevant estimation of 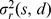. For a given type of residue pair 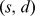, if 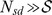, then no correction is necessary, and eq. **12** reduces to eq. **2**. On the contrary, if 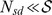, then the data on 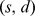 pairs is considered too sparse to reliably estimate 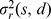, and 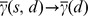. We found that the value of 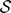 has little impact on the overall quality of the model, as long as it is not too small (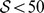), in which case aberrant values of 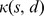 are determined for some uncommon 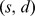 pairs, or too large (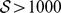), in which case the performances decrease because of a loss of information on sequence-specificity. The value of the parameter 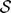 was set here to 500.

The 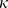 values were rescaled after each iteration step, so that the average value of 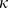 over all amino acid types is equal to 1 for pairs separated by a distance of 6 Å. Residue pairs of a given type 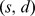 for which 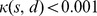 (after rescaling), were considered to establish no direct interaction: 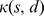 was set to 0, and they were no longer considered in the iterative procedure. The performances of the new ENM variants after the first nine iteration steps are reported in Table S1. The procedure converged rapidly for the dENM and the sdENM, and the final models were selected after 5 and 3 iteration steps, respectively. The sENM variants did not improve significantly with respect to the initial models (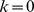), indicating that 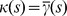 is a good approximation, contrary to 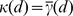. The procedure was thus stopped after one iteration step, for both the 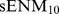 and the 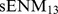.

To assess the robustness of the derivation scheme, and the sensitivity of the 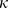 values determined for each type of residue pair to the content of the dataset, we calculated the bootstrap estimates of the 90% confidence intervals on 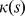, 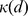, and 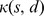. For that purpose, the iterative procedure was repeated with 100 different datasets, each one consisting of 1500 NMR ensembles randomly picked, with replacement, from the original training dataset. All 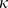 values, and the corresponding confidence intervals, are given in Dataset S1.

### Performance measures

The ability of coarse-grained models to accurately describe protein dynamics is commonly evaluated by computing the Pearson correlation coefficient between predicted and experimental MSRF, 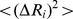, over all 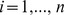 residues of a given protein:
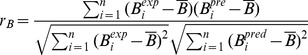
(13)where, for simplicity, 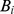 was used instead of 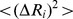. There is indeed a direct relationship between the MSRF and the cristallographic B-factors: 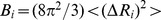. 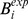 and 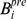 correspond thus here to the MSRF of residue 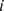 extracted from the NMR data and predicted by the ENM, respectively. The scale of the predicted MSRF values depends on the scale of the spring constants, which are only defined up to a constant factor. This factor was determined, for each protein independently, by fitting the scales of the predicted and experimental MSRF, i.e. to ensure that:
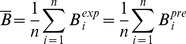
(14)


Although it has been widely used in previous studies, 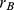 is probably not the most adequate measure to evaluate the performances of coarse-grained models of protein dynamics. As pointed out previously [26], [27], it does indeed present several shortcomings: e.g. it is strongly affected by the presence of highly flexible regions, and does not account for possible flaws leading to an intercept of the regression line different from zero. Most importantly, the MSRF describe individual fluctuations but provide no information about the cooperative aspects of residue motions. The quality of the MSRF predictions gives thus no guarantee about the ability of the model to describe the cooperativity of protein dynamics. The 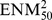 provides an interesting example, for it performs quite well in predicting the MSRF but basically negates all cooperativity (Figure 2, Table 1).

Therefore, we introduce a new measure that exploits the information contained in the correlation matrix 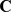, to quantify the error on the estimation of the fluctuations of the interresidue distances:
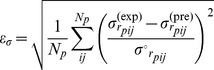
(15)where 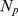 is the number of non-bonded residue pairs in protein 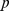, 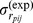 and 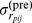 are the experimental (eq. **9**) and predicted (eq. **10**) values of 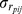, respectively. 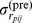 is obtained after fitting the experimental MSRF with the predicted ones (eq. **14**). The error is normalized by 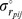, which is the expected value of 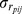 given the individual, anisotropic, fluctuations of both residues extracted from the NMR data, but neglecting all correlations between their respective motions. This normalization ensures that the contributions of the different pairs of residues are equivalent, and that the measure is not dominated by highly flexible regions.

Both 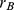 and 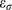 are computed independently for each of the 349 proteins of our test set, and the average values are reported. We also report the short- (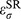), mid- (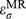), and long-range (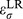) contributions to 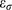, obtained by considering only pairs separated by 0–15 Å, 15–30 Å, and more than 30 Å, respectively.

## Supporting Information

Dataset S1
**Spring constants of the** 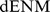 **,** 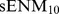 **,** 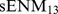 **,** 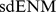 **(plain text).**
(BZ2)

Figure S1
**Comparison of the apparent stiffness** 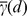 **extracted from different protein datasets.** The different lines correspond to the full dataset of 1500 proteins (bold line), a subset of 646 small (i.e. less than 100 residues) proteins (green), a subset of 225 larger (i.e. more than 150 residues) proteins (magenta), a subset of 253 all-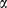 proteins (red), and a subset of 200 all-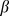 proteins (blue).(PDF)

Figure S2
**Comparison of the experimental and predicted apparent stiffness** 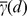 **on two individual proteins.** (A) Schematic representation of the structural ensemble of ubiquitin, obtained by combining NMR information with molecular dynamics simulations (PDB: 1xqq) [38]. (B) Schematic representation of the NMR structural ensemble of periplasmic chaperone FimC (PDB: 1bf8). The relatively rigid orientation of the two domains is ensured by specific interdomain interactions [39]. (C–D) Comparison of the experimental and predicted values of the apparent stiffness 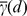 extracted from either of these two proteins. The bold black curves correspond to the experimental values of 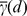. The other curves correspond to the values of 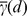 predicted by different ENM variants: 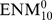 (dashed red), 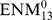 (continuous red); 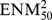 (dashed blue); 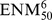 (continuous blue), 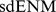 (continuous green). The grey curves correspond to the experimental values of 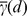 extracted from the full dataset of 1500 proteins.(PDF)

Figure S3
**Comparison of the experimental and predicted apparent stiffness** 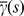 **on the dataset of 1500 NMR ensembles.** (A–E) For each amino acid, the median value of 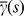 over the 20 possible partners is given in units of 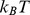, along with the maximal, minimal, 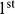 and 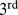 quartile values. Only residue pairs separated by an equilibrium distance of 10 Å, at most, were considered. (F–I) The predicted values of 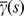 are plotted against the experimental ones.(PDF)

Figure S4
**Correlation between spring constants and contact potentials.** The energy values of the static contact potentials previously derived by Miyazawa and Jernigan [30] are plotted against the spring constants of the 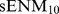, for the 210 amino acid pairs.(PDF)

Figure S5
**Comparison of the experimental and predicted apparent stiffness** 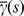 **on the dataset of 1500 NMR ensembles.** (A–E) For each amino acid, the median value of 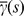 over the 20 possible partners is given in units of 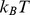, along with the maximal, minimal, 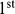 and 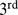 quartile values. Only residue pairs separated by an equilibrium distance of 10 Å, at most, were considered. (F–I) The predicted values of 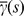 are plotted against the experimental ones.(PDF)

Figure S6
**Comparison of the apparent stiffness** 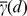 **computed with or without the linear approximation.** The black bold line corresponds to the apparent stiffness observed in the test set of 349 proteins. The red and blue lines correspond to the apparent stiffness predicted by the 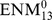 and the 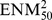, respectively, on the same dataset. The continuous lines were obtained in the context of the linear approximation, using eqs. **2** and **10**. The dashed lines were obtained by following, for each protein, the motions of the residues in the elastic network subjected to gaussian noise, during 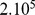 integration steps. 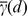 was subsequently computed using eqs. **2** and **9**. We ensured that the sampling was sufficient by comparing the MSRF extracted from these trajectories with those computed from the correlation matrix (eqs. **7**, **8**). The correlation coefficient between these two sets of MSRF values was equal to 0.95 for the 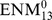 and 0.98 for the 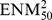, on average over the 349 proteins of the test set. However, for some proteins (46 with the 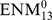 and 6 with the 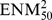), the length of the simulation appeared to be insufficient, as the correlation coefficient between the MSRF obtained from both approaches was lower than 0.9. These proteins were discarded from the comparison.(PDF)

Table S1 **Performances of the new ENM during the iterative procedure.**
(PDF)

Table S2
**Spring constants of the** 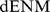 **.**
(PDF)

Table S3
**Spring constants of the** 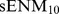 **.**
(PDF)

Table S4
**Spring constants of the** 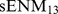 **.**
(PDF)

Table S5
**Spring constants of the** 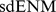 **.**
(PDF)

Text S1 **Derivation of effective harmonic potentials.**
(PDF)
