# Supplementary figures and images for: Effective Harmonic Potentials: Insights into the Internal Cooperativity and Sequence-Specificity of Protein Dynamics
Source: PLoS Comput Biol. 2013 Aug 29;9(8):e1003209. doi: 10.1371/journal.pcbi.1003209 (PMC3757084; doi:10.1371/journal.pcbi.1003209)

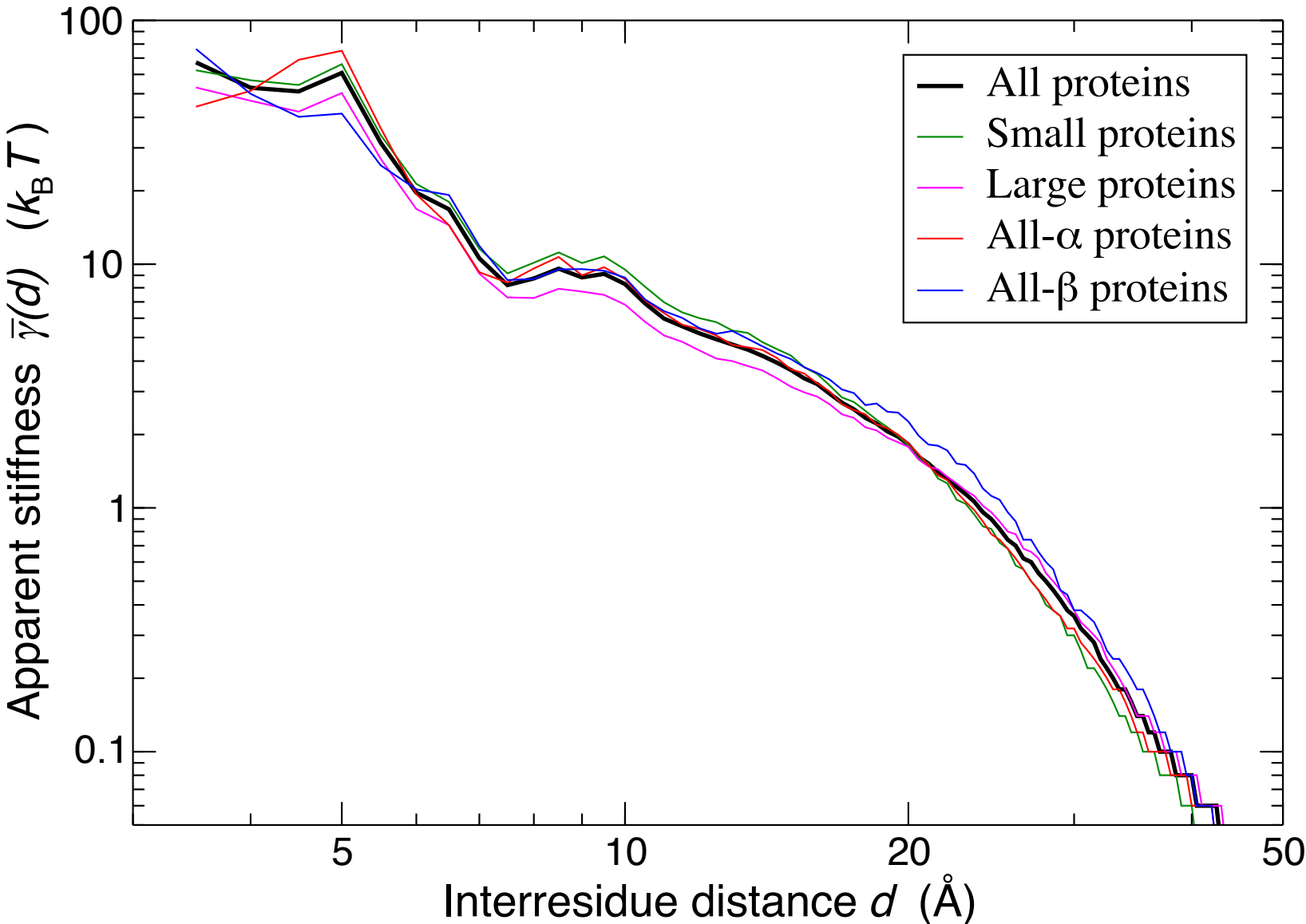

Supplement: Figure S1 — Comparison of the apparent stiffness extracted from different protein datasets. The different lines correspond to the full dataset of 1500 proteins (bold line), a subset of 646 small (i.e. less than 100 residues) proteins (green), a subset of 225 larger (i.e. more than 150 residues) proteins (magenta), a subset of 253 all- proteins (red), and a subset of 200 all- proteins (blue). (PDF) [file pcbi.1003209.s002.pdf]

**A**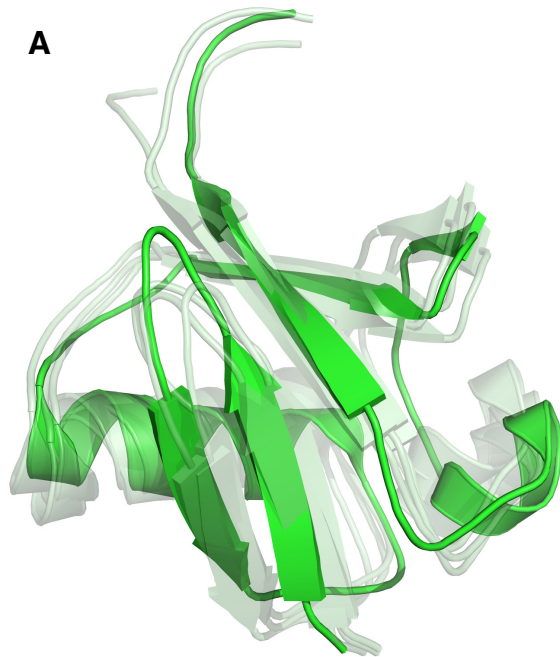**B**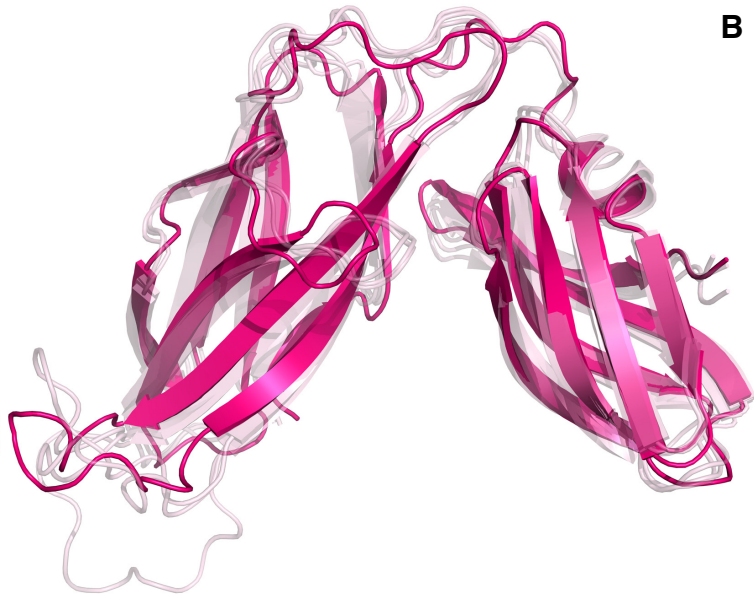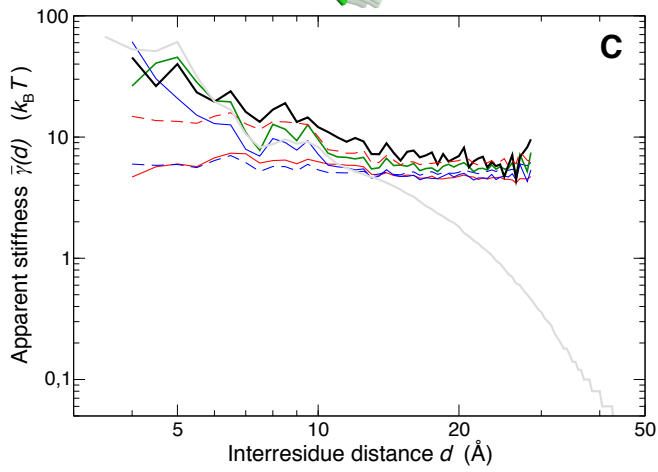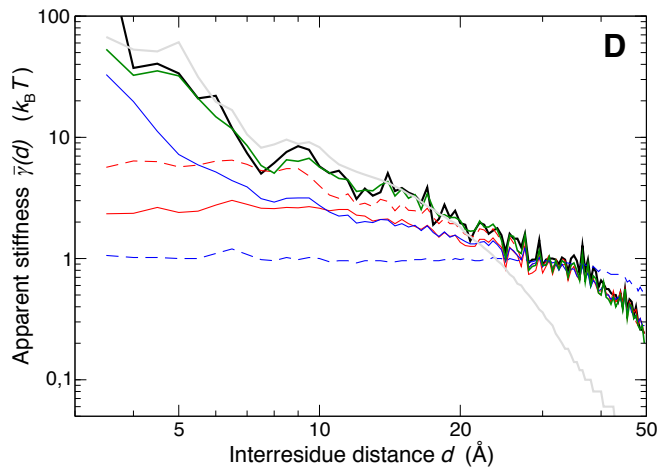

Supplement: Figure S2 — Comparison of the experimental and predicted apparent stiffness on two individual proteins. (A) Schematic representation of the structural ensemble of ubiquitin, obtained by combining NMR information with molecular dynamics simulations (PDB: 1xqq) [38]. (B) Schematic representation of the NMR structural ensemble of periplasmic chaperone FimC (PDB: 1bf8). The relatively rigid orientation of the two domains is ensured by specific interdomain interactions [39]. (C–D) Comparison of the experimental and predicted values of the apparent stiffness extracted from either of these two proteins. The bold black curves correspond to the experimental values of . The other curves correspond to the values of predicted by different ENM variants: (dashed red), (continuous red); (dashed blue); (continuous blue), (continuous green). The grey curves correspond to the experimental values of extracted from the full dataset of 1500 proteins. (PDF) [file pcbi.1003209.s003.pdf]

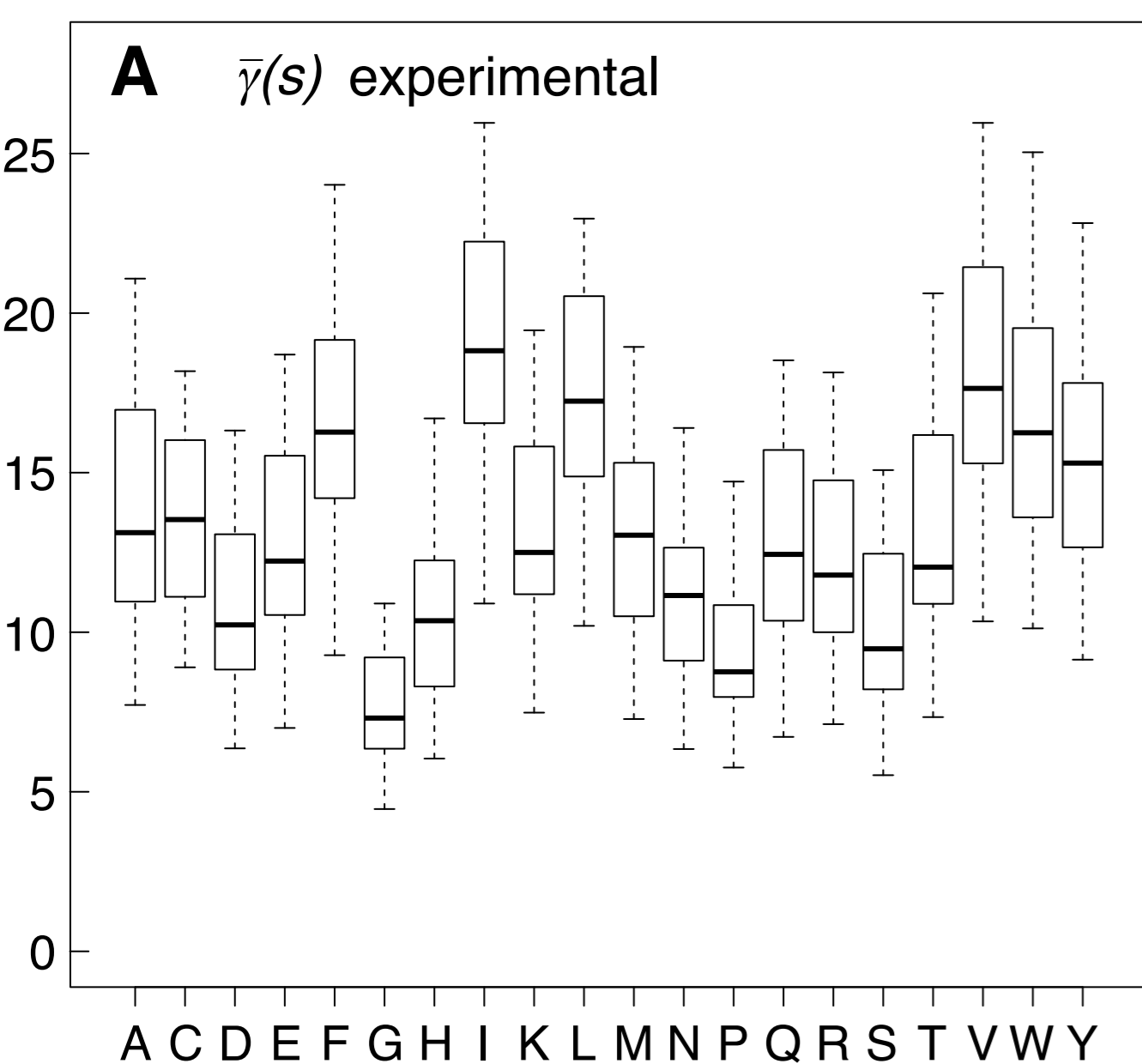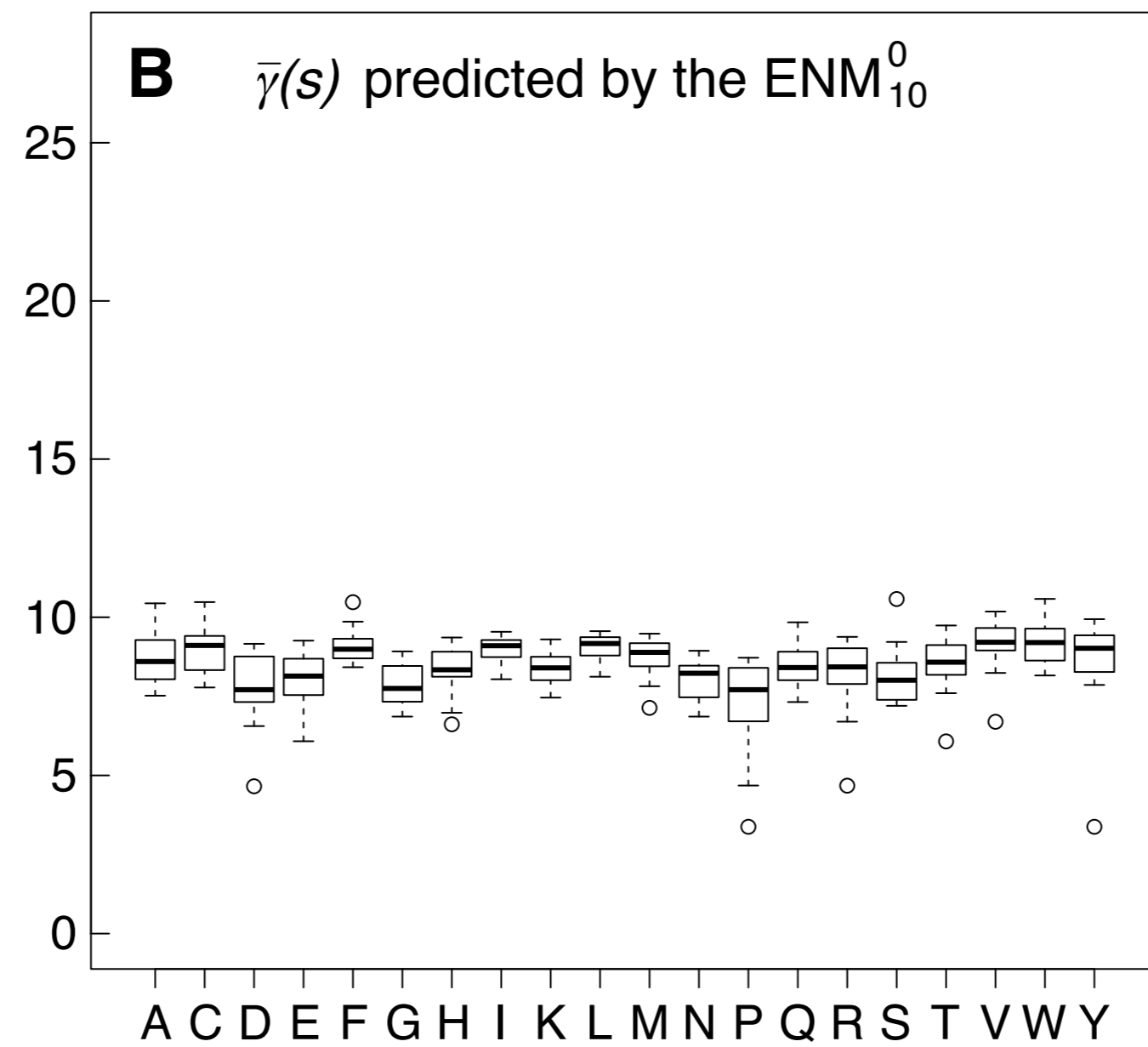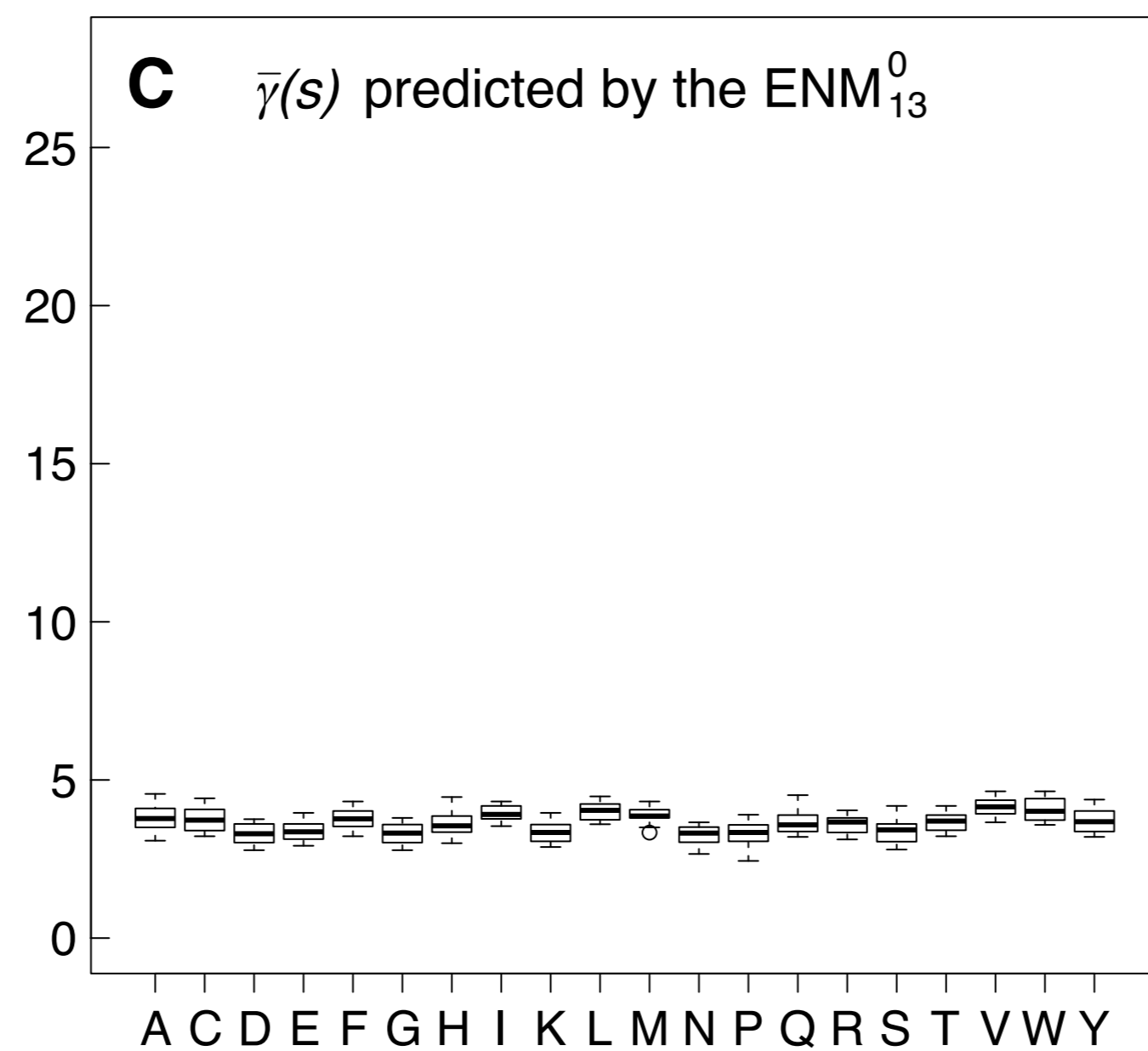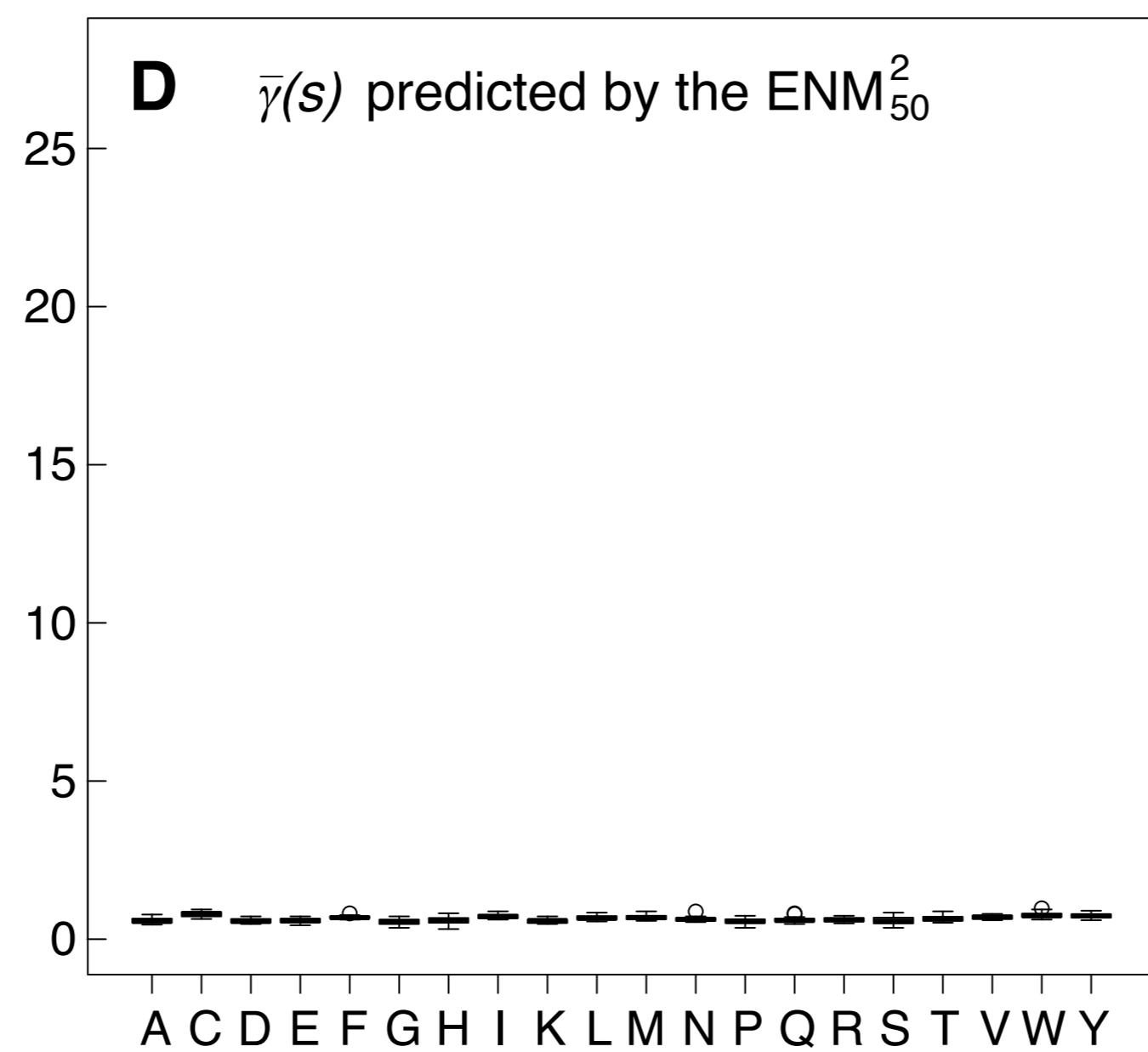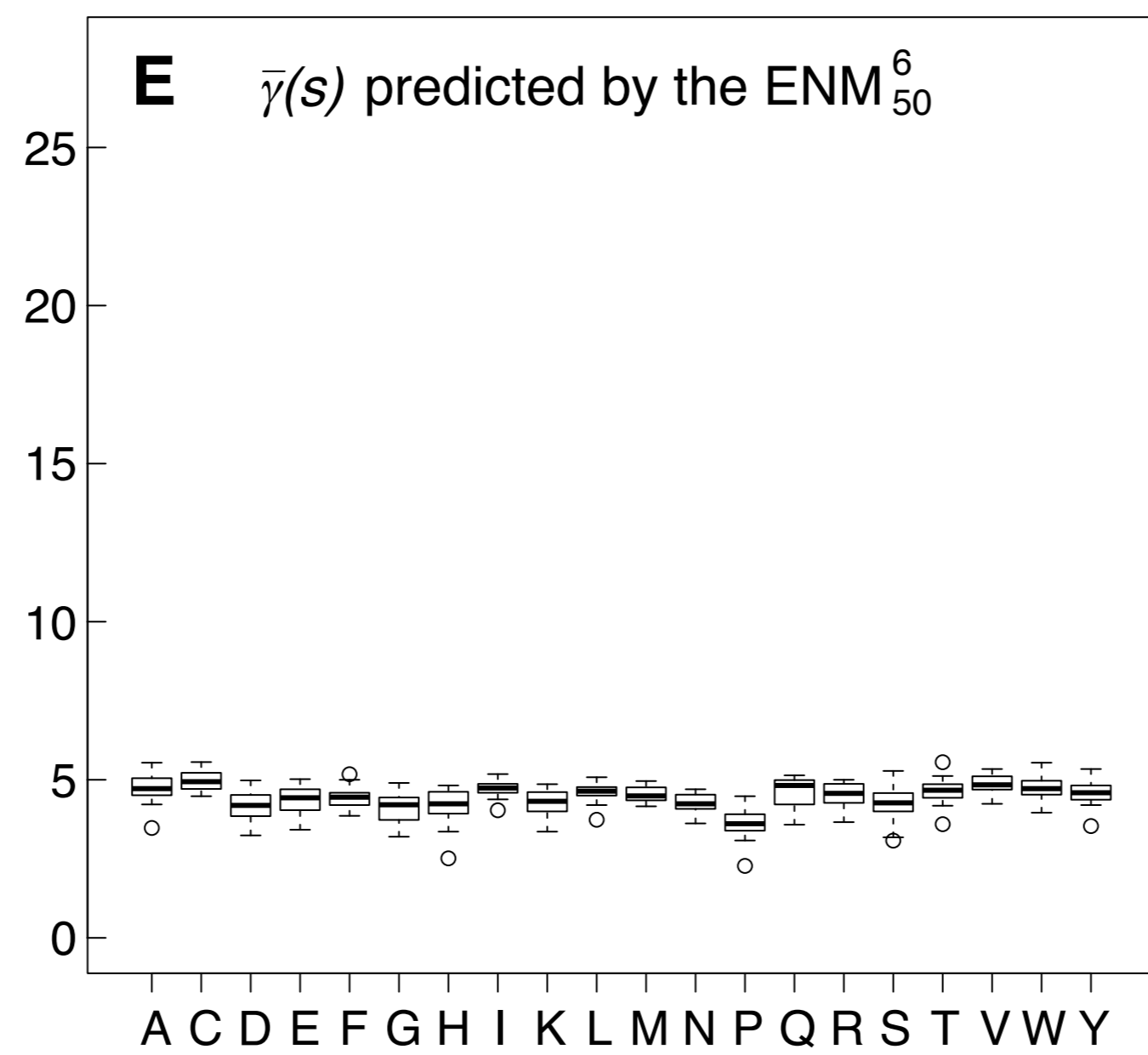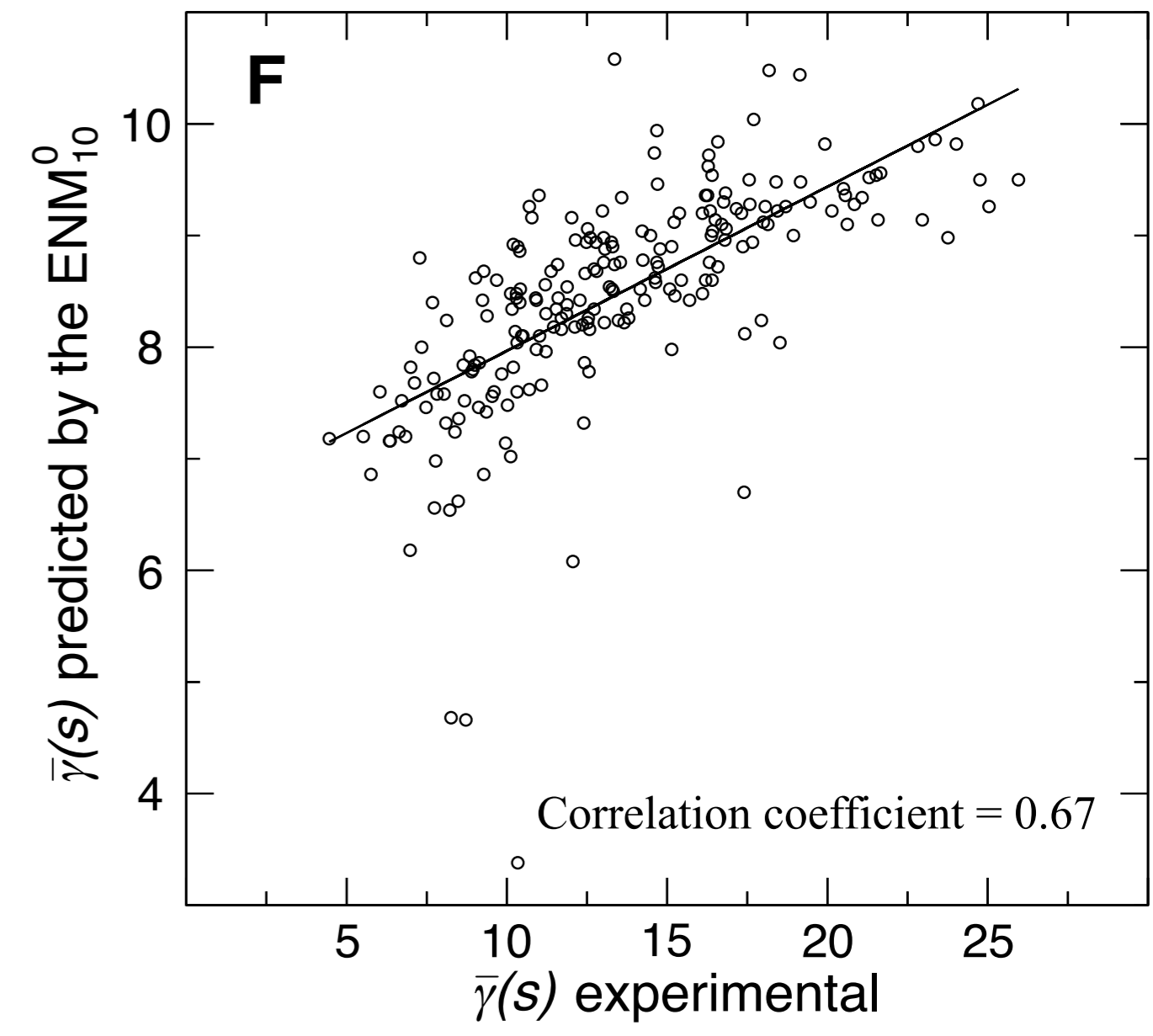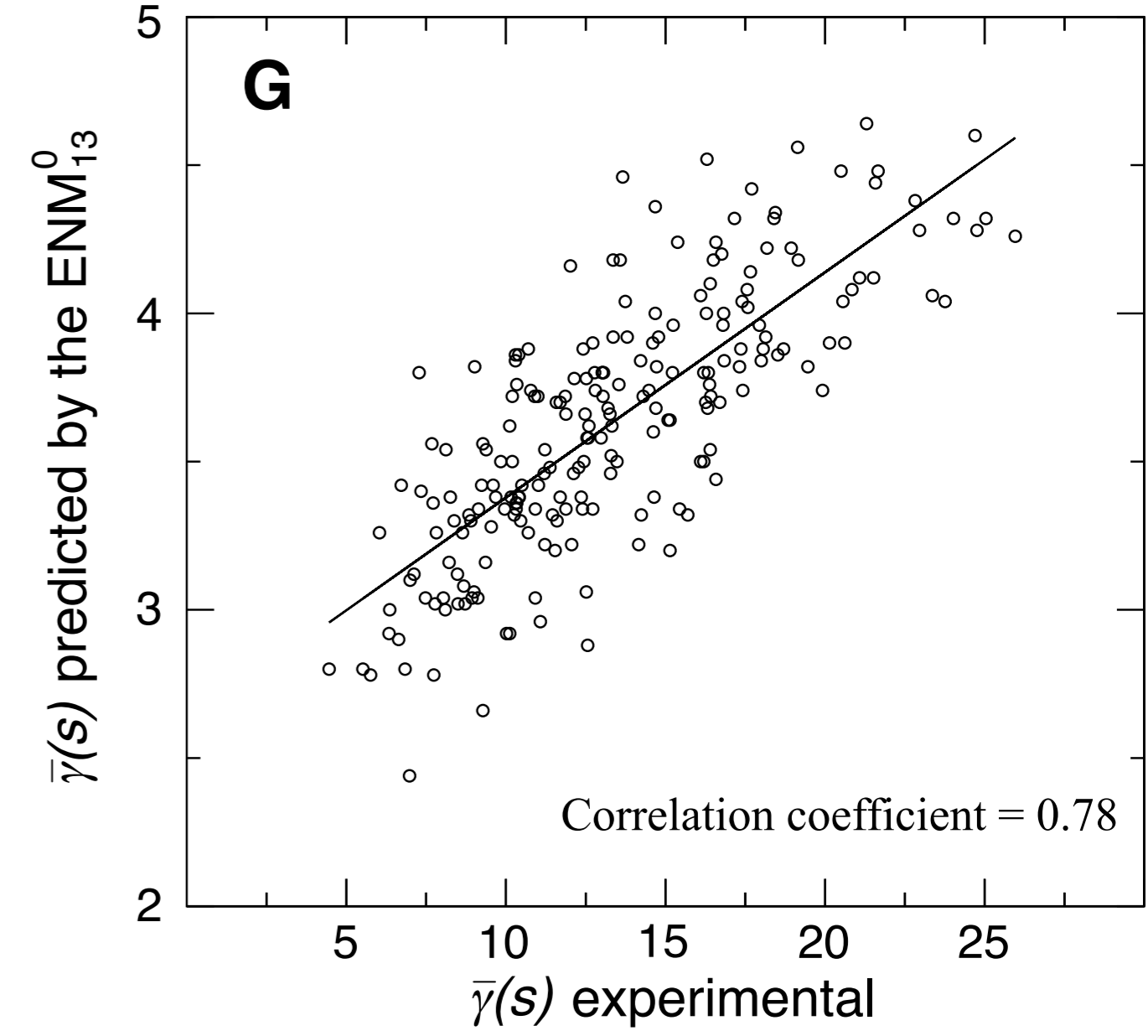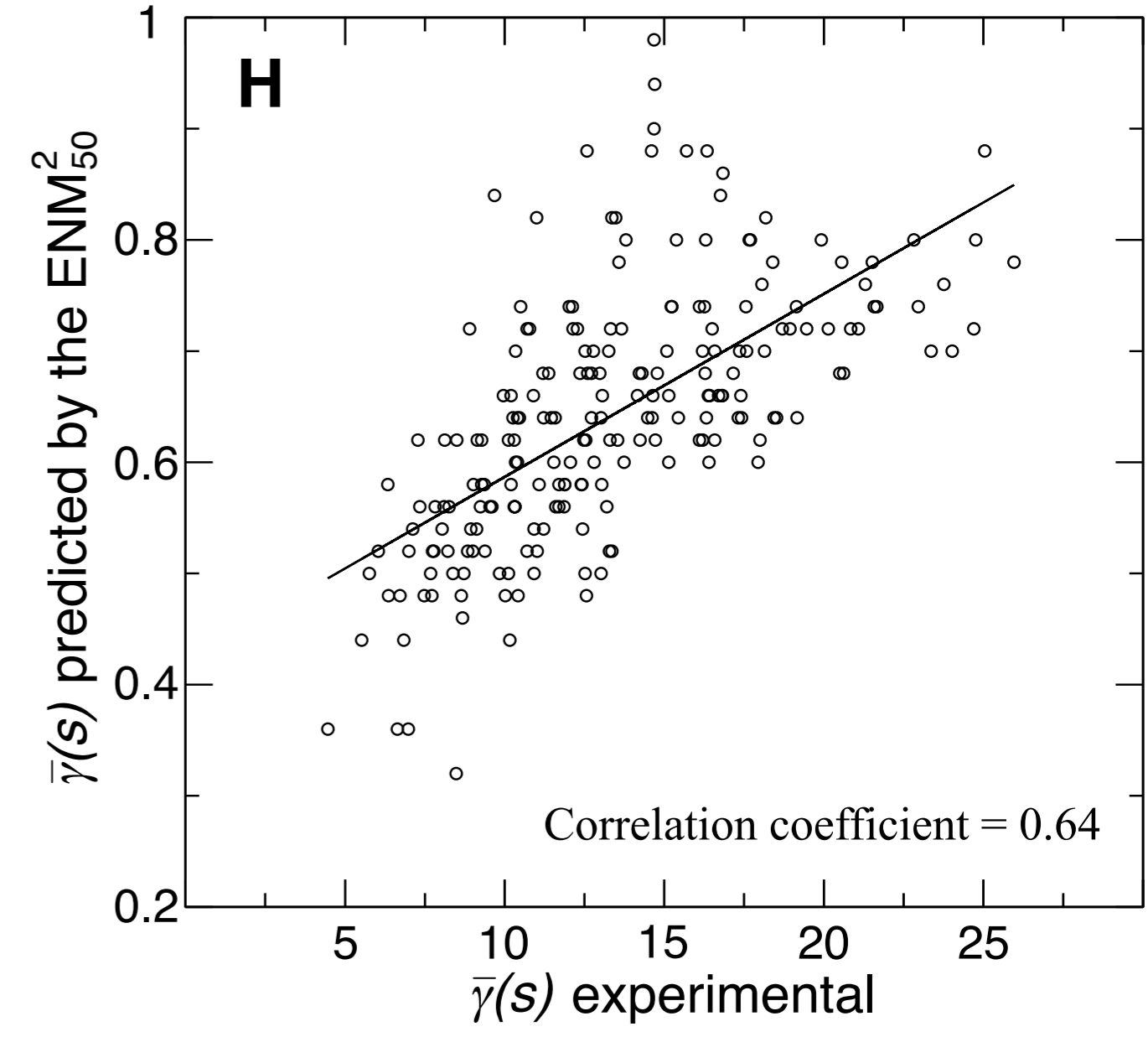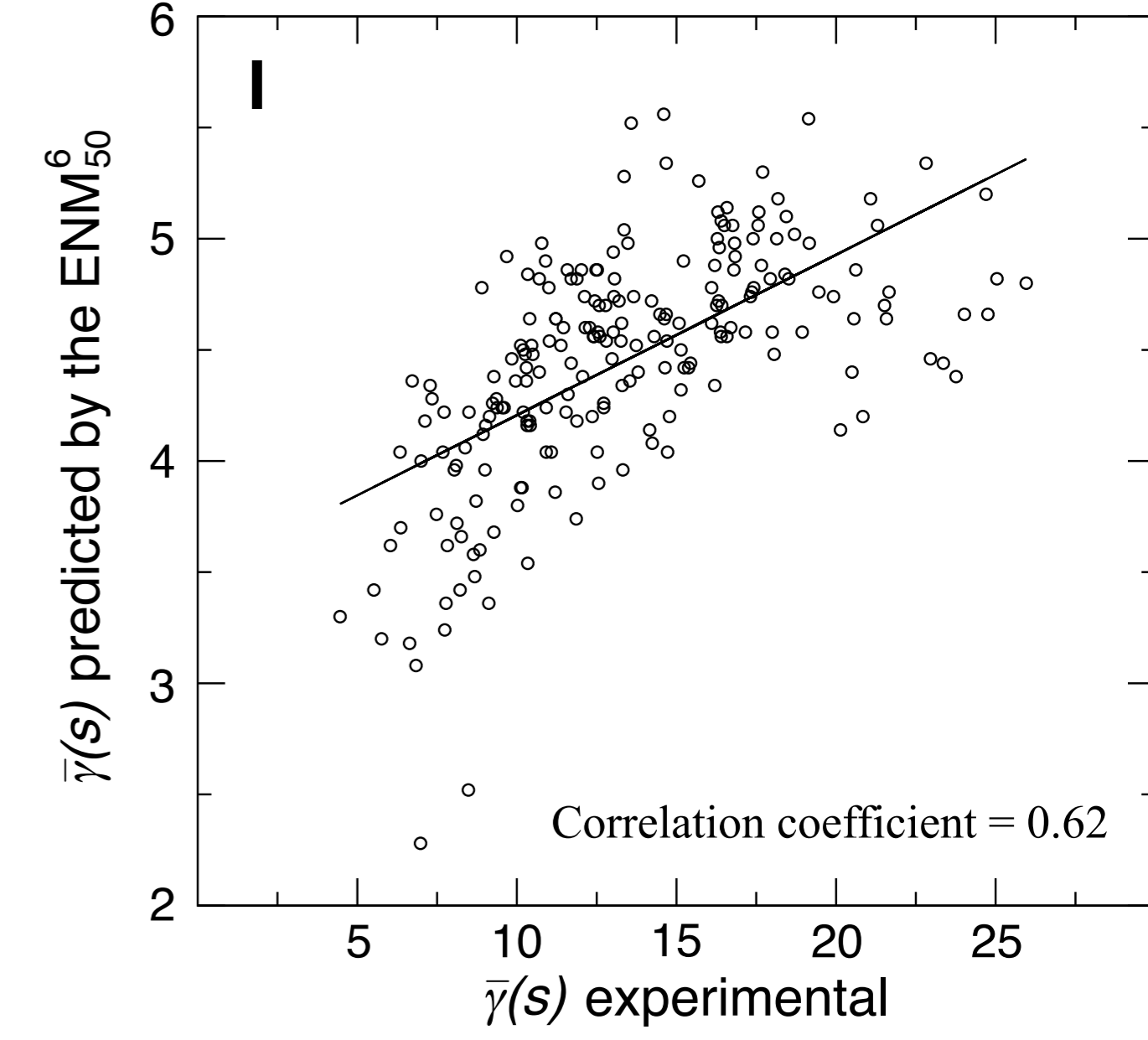

Supplement: Figure S3 — Comparison of the experimental and predicted apparent stiffness on the dataset of 1500 NMR ensembles. (A–E) For each amino acid, the median value of over the 20 possible partners is given in units of , along with the maximal, minimal, and quartile values. Only residue pairs separated by an equilibrium distance of 10 Å, at most, were considered. (F–I) The predicted values of are plotted against the experimental ones. (PDF) [file pcbi.1003209.s004.pdf]

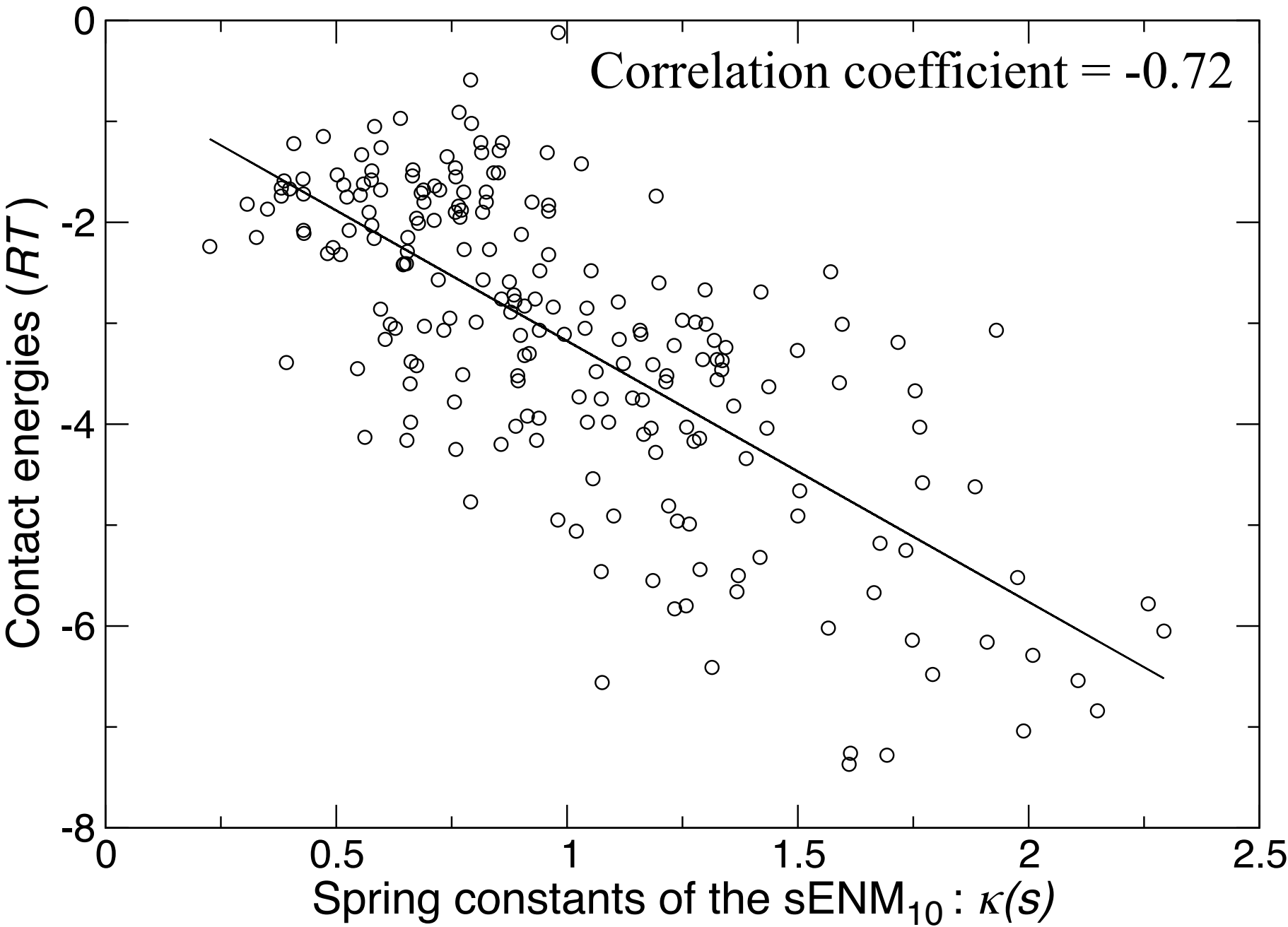

Supplement: Figure S4 — Correlation between spring constants and contact potentials. The energy values of the static contact potentials previously derived by Miyazawa and Jernigan [30] are plotted against the spring constants of the , for the 210 amino acid pairs. (PDF) [file pcbi.1003209.s005.pdf]

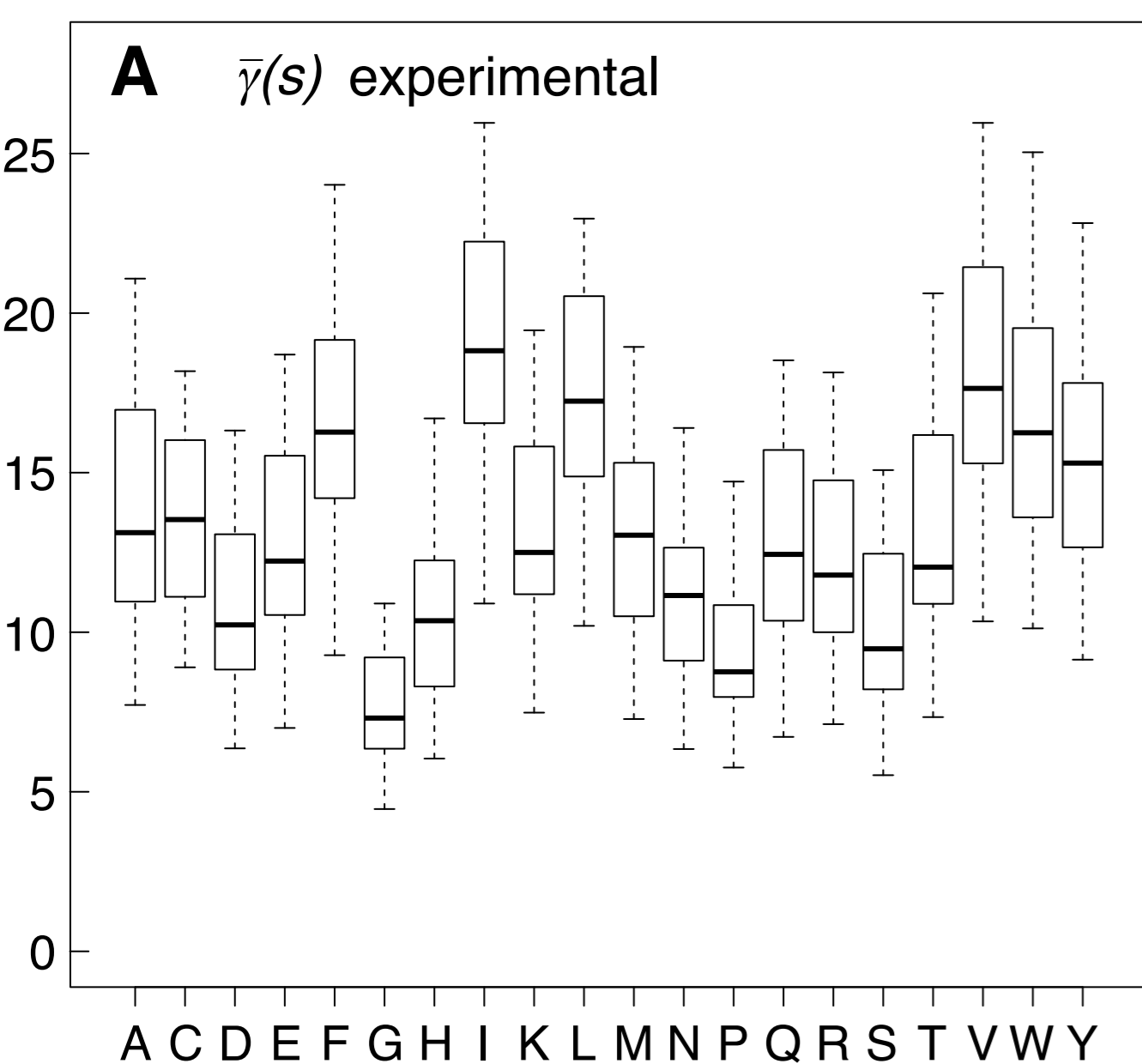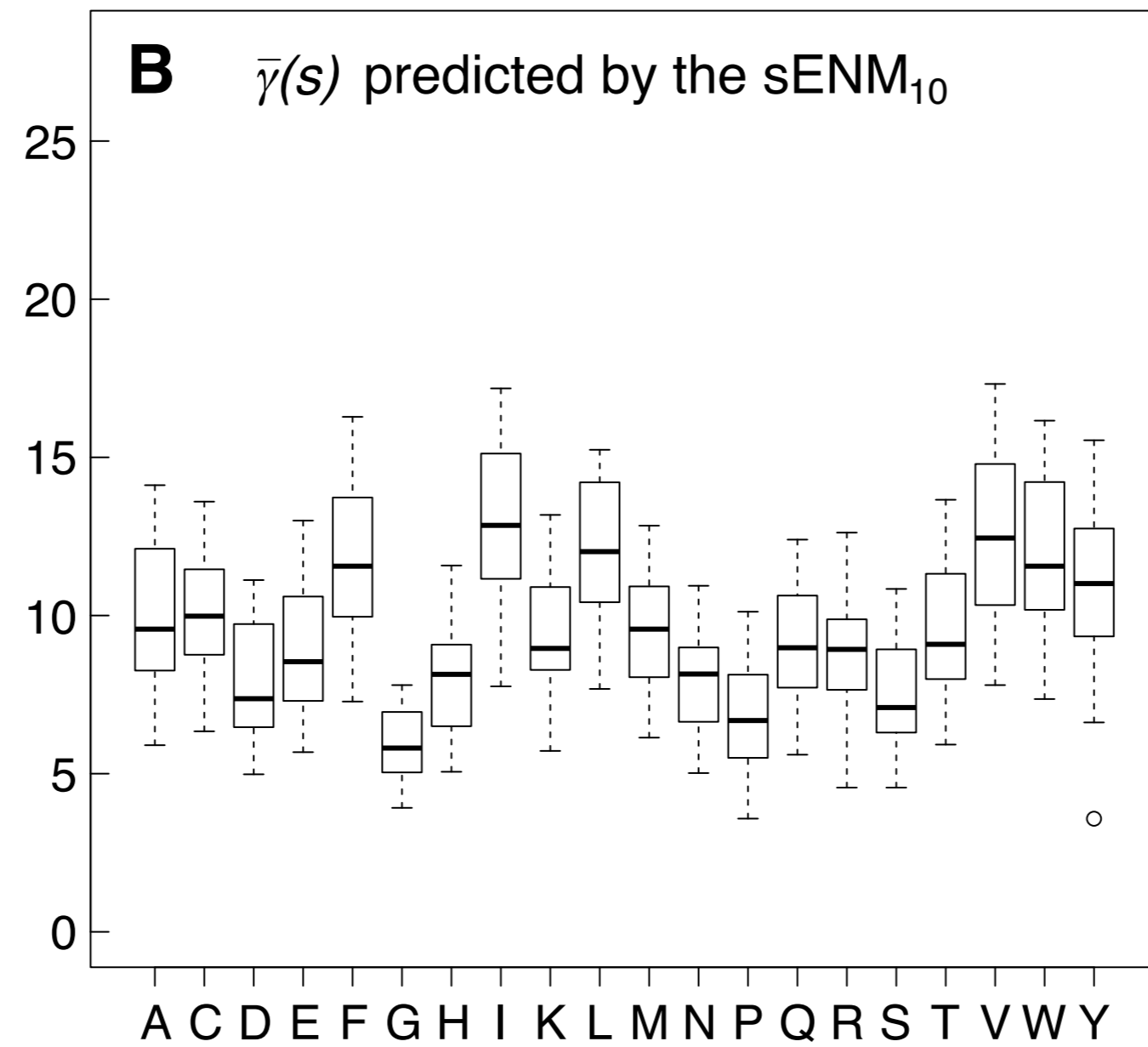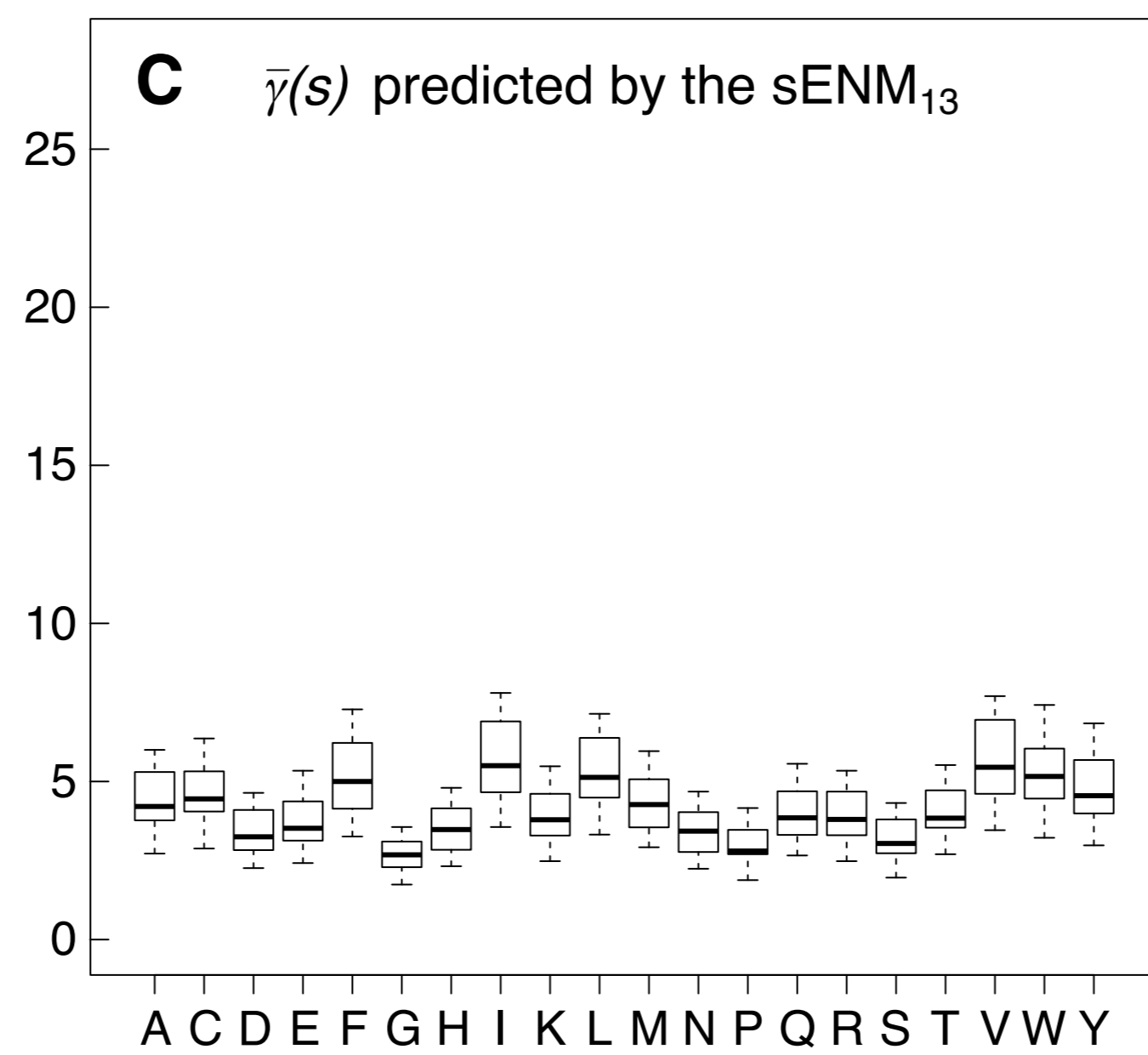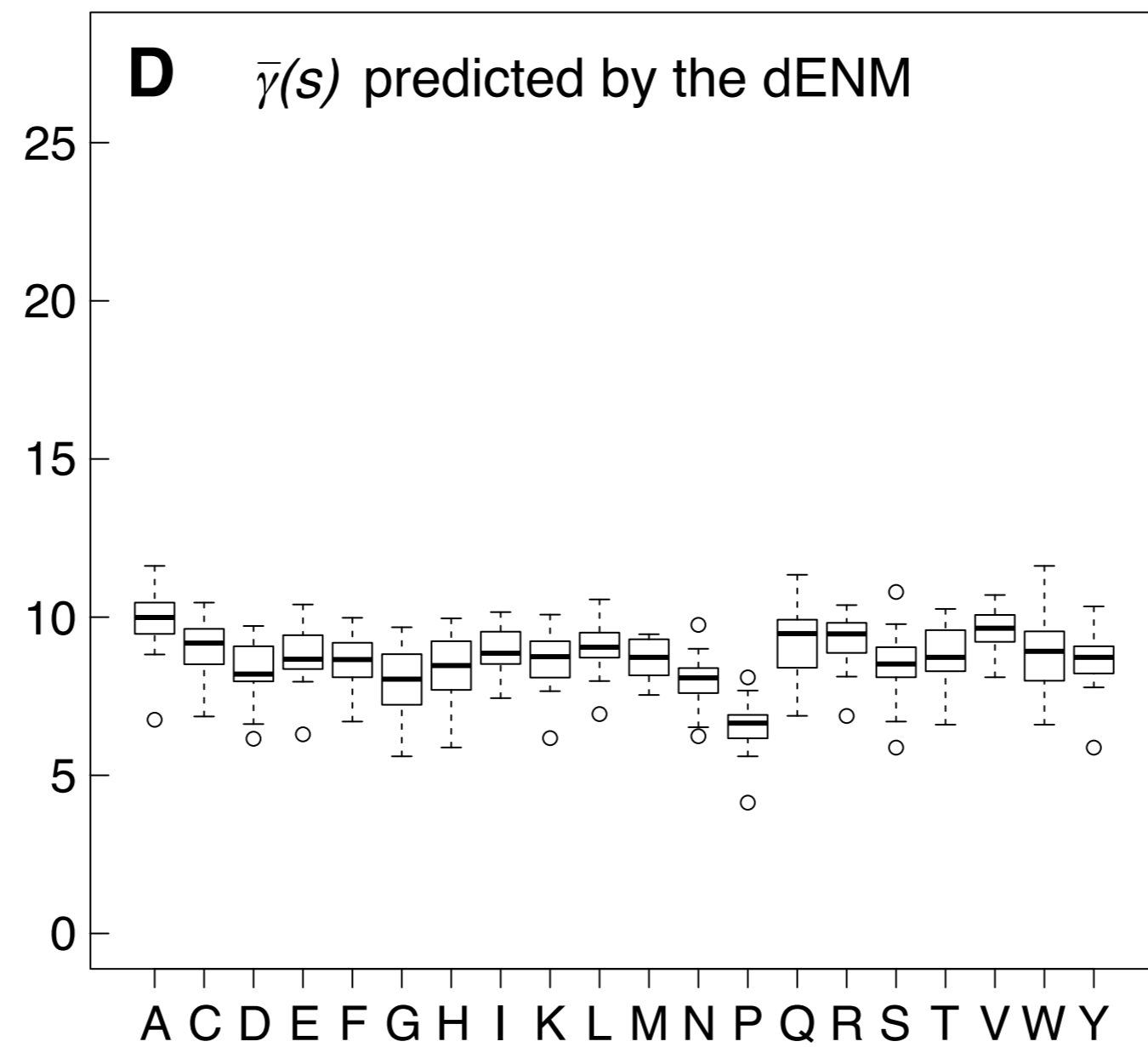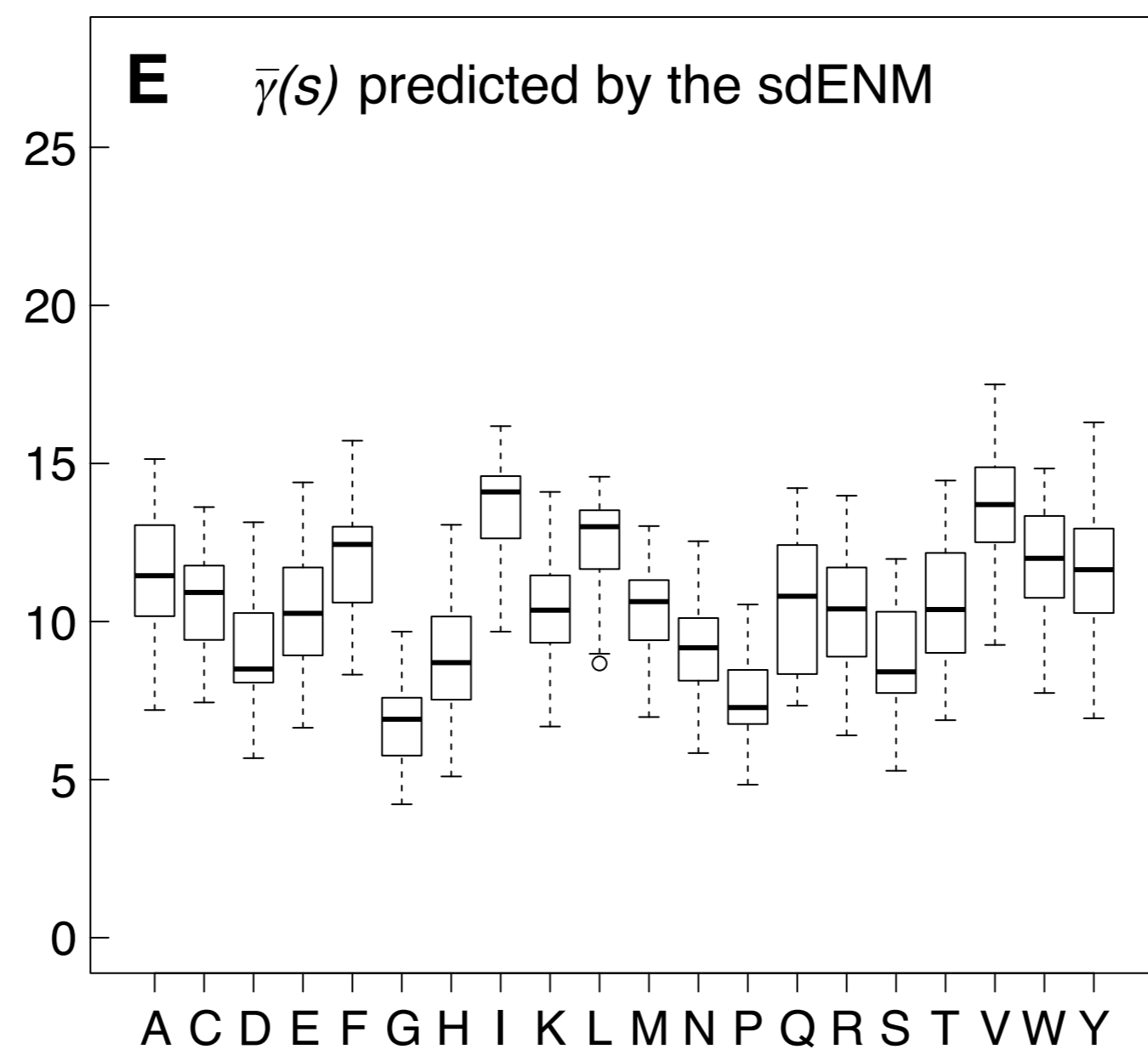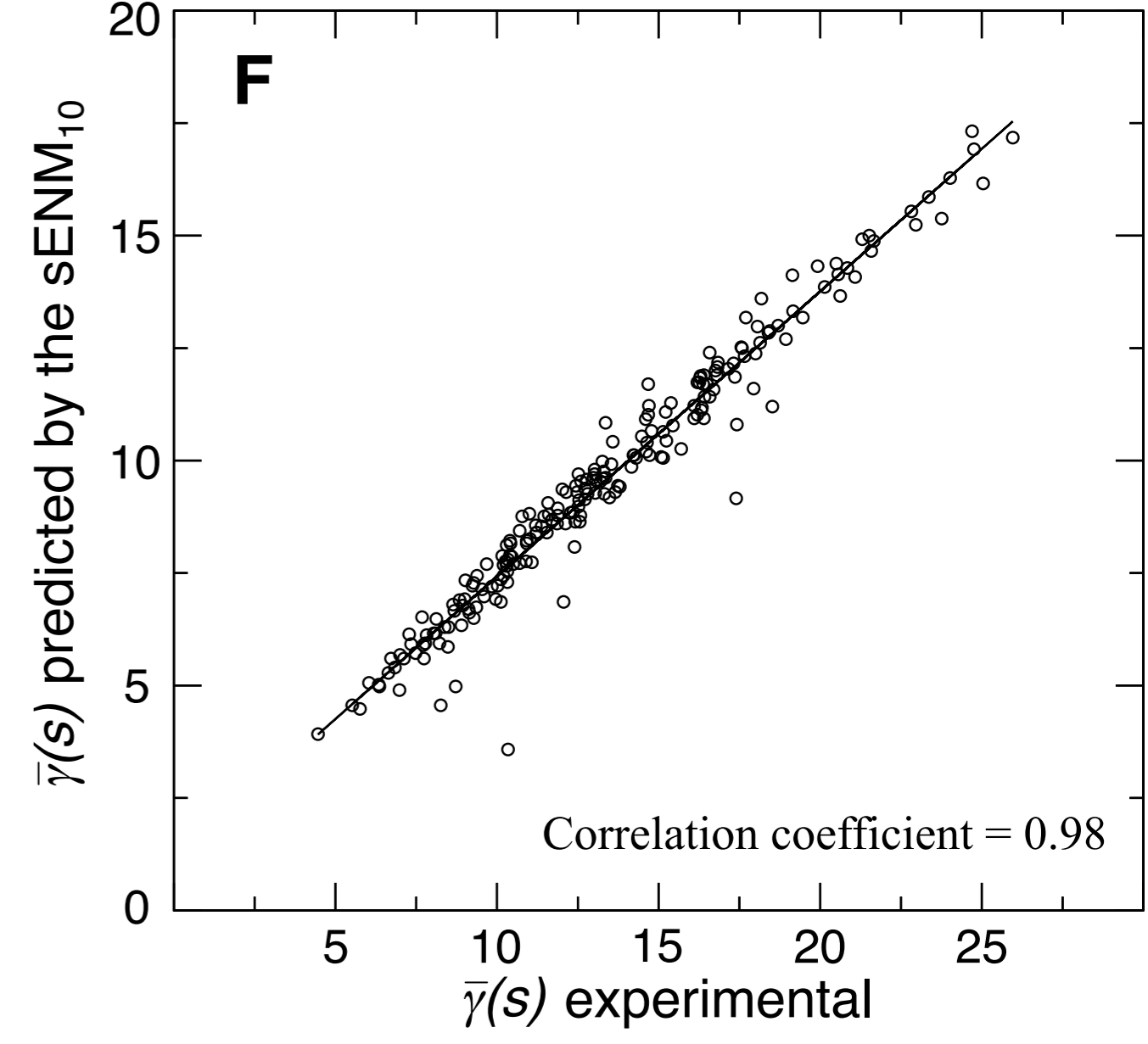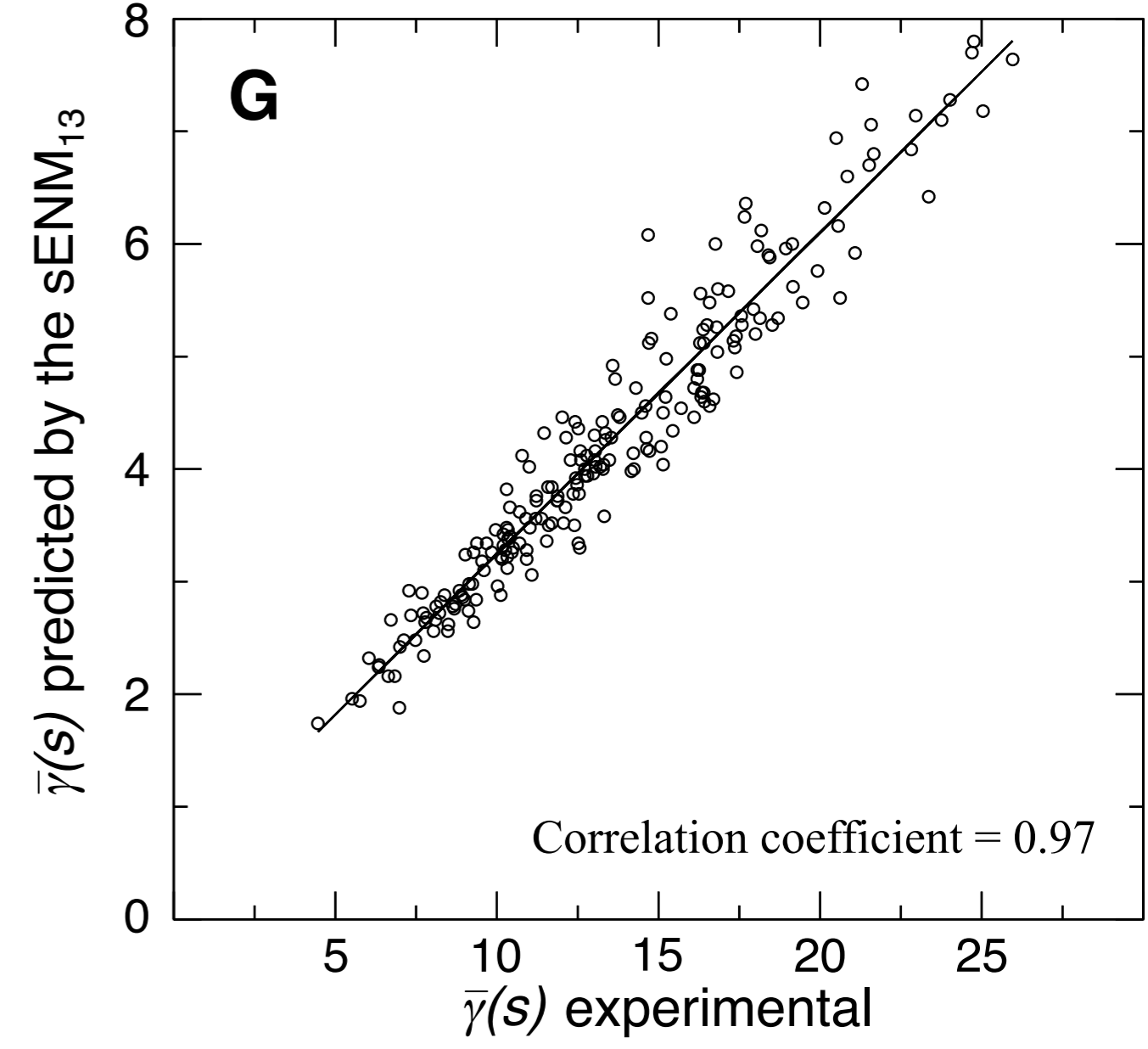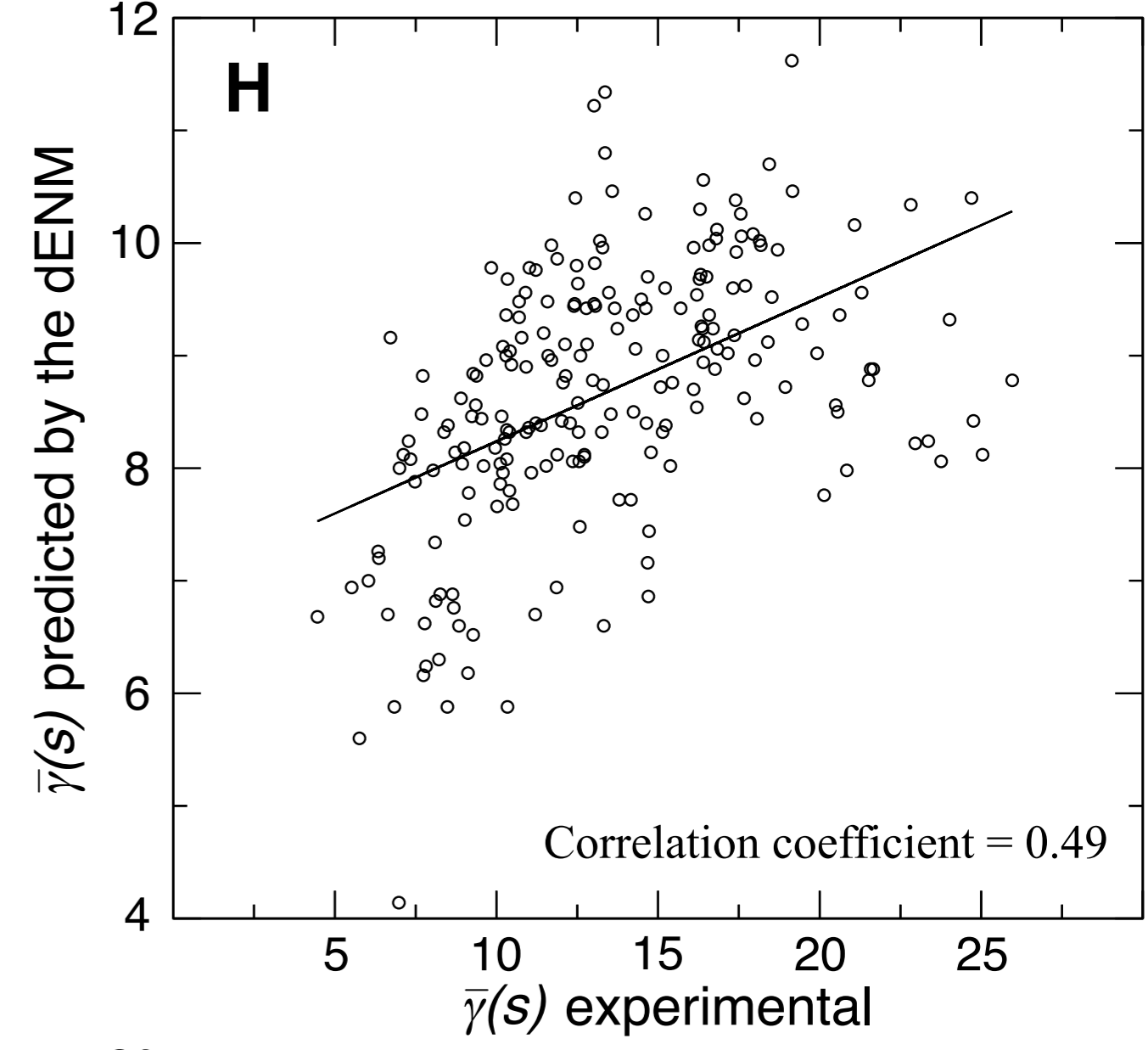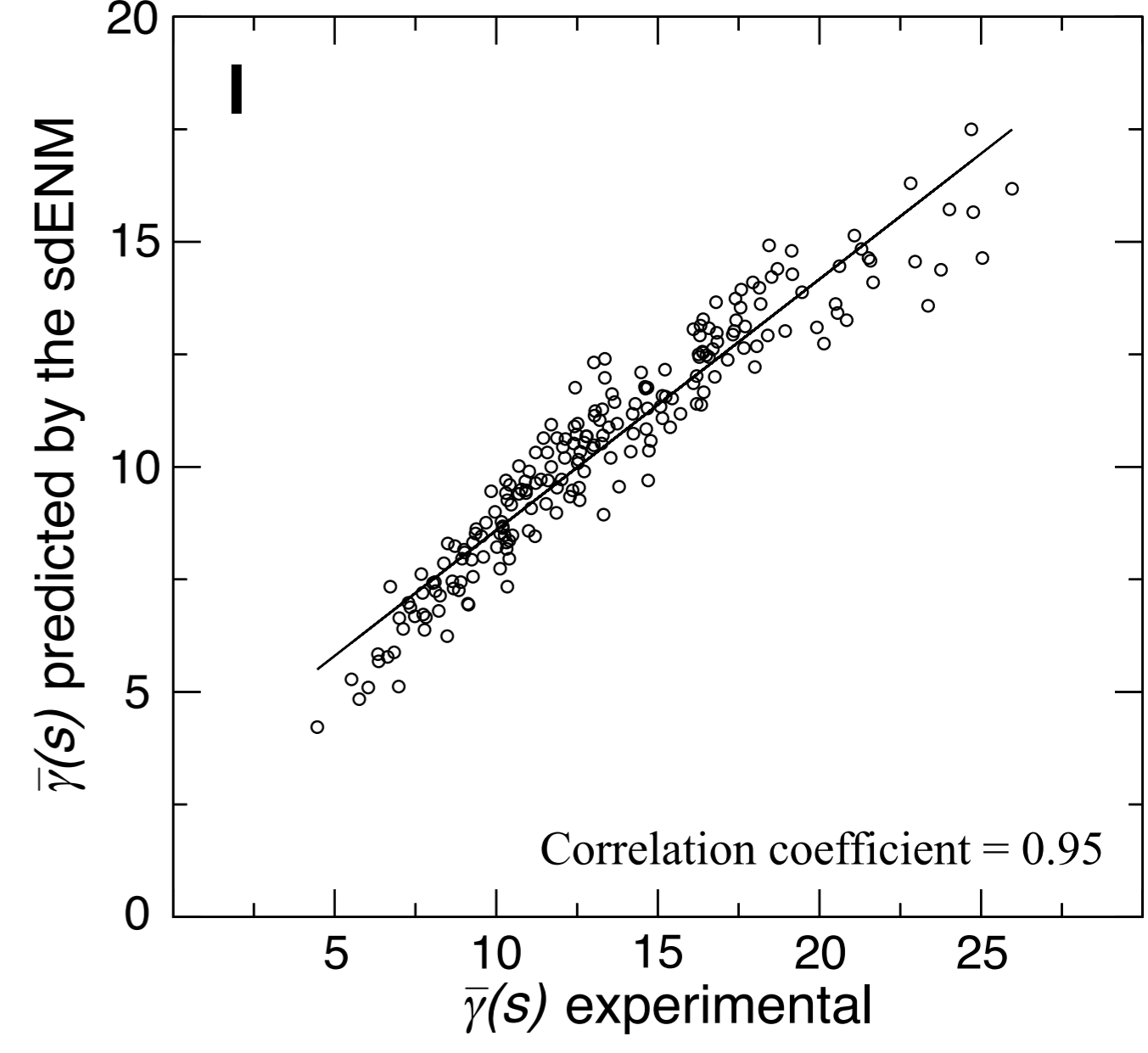

Supplement: Figure S5 — Comparison of the experimental and predicted apparent stiffness on the dataset of 1500 NMR ensembles. (A–E) For each amino acid, the median value of over the 20 possible partners is given in units of , along with the maximal, minimal, and quartile values. Only residue pairs separated by an equilibrium distance of 10 Å, at most, were considered. (F–I) The predicted values of are plotted against the experimental ones. (PDF) [file pcbi.1003209.s006.pdf]

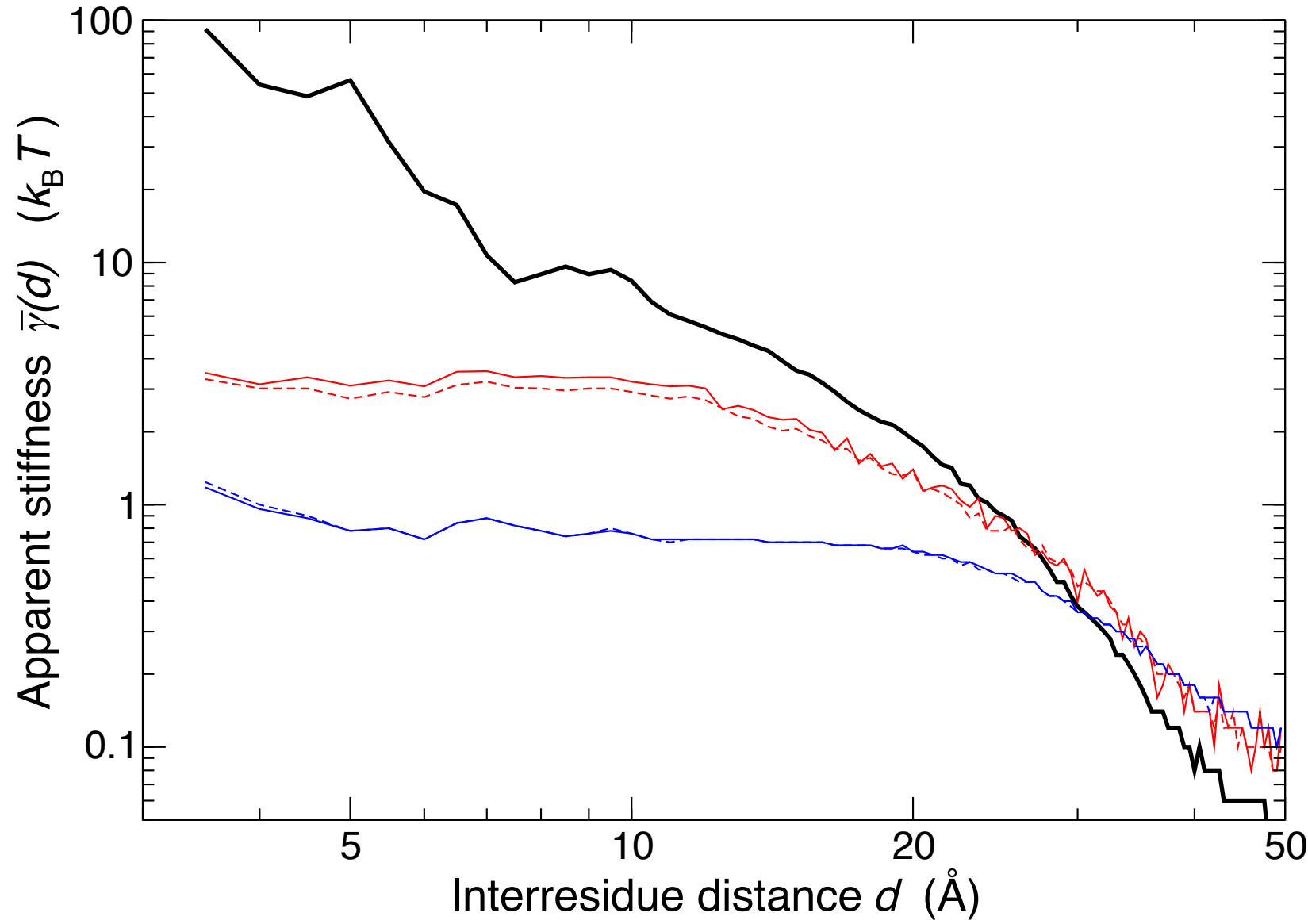

Supplement: Figure S6 — Comparison of the apparent stiffness computed with or without the linear approximation. The black bold line corresponds to the apparent stiffness observed in the test set of 349 proteins. The red and blue lines correspond to the apparent stiffness predicted by the and the , respectively, on the same dataset. The continuous lines were obtained in the context of the linear approximation, using eqs. 2 and 10 . The dashed lines were obtained by following, for each protein, the motions of the residues in the elastic network subjected to gaussian noise, during integration steps. was subsequently computed using eqs. 2 and 9 . We ensured that the sampling was sufficient by comparing the MSRF extracted from these trajectories with those computed from the correlation matrix (eqs. 7 , 8 ). The correlation coefficient between these two sets of MSRF values was equal to 0.95 for the and 0.98 for the , on average over the 349 proteins of the test set. However, for some proteins (46 with the and 6 with the ), the length of the simulation appeared to be insufficient, as the correlation coefficient between the MSRF obtained from both approaches was lower than 0.9. These proteins were discarded from the comparison. (PDF) [file pcbi.1003209.s007.pdf]
