# Supplementary material for: Effective Harmonic Potentials: Insights into the Internal Cooperativity and Sequence-Specificity of Protein Dynamics
Source: PLoS Comput Biol. 2013 Aug 29;9(8):e1003209. doi: 10.1371/journal.pcbi.1003209 (PMC3757084; doi:10.1371/journal.pcbi.1003209)
Supplement: Table S1 — Performances of the new ENM during the iterative procedure. (PDF) [file pcbi.1003209.s008.pdf]

Supporting Table S1: Performances of the new ENM during the iterative procedure

Iteration step	dENM		$sENM_{10}$		$sENM_{13}$		sdENM	
	r_B	ϵ_σ	r_B	ϵ_σ	r_B	ϵ_σ	r_B	ϵ_σ
$k = 0$	0.67	0.92	0.63	0.56	0.66	0.64	0.69	0.54
$k = 1$	0.68	0.75	**0.63**	**0.55**	**0.66**	**0.63**	0.70	0.51
$k = 2$	0.69	0.64	0.63	0.55	0.66	0.63	0.70	0.49
$k = 3$	0.69	0.59	0.63	0.55	0.66	0.63	**0.70**	**0.48**
$k = 4$	0.69	0.56	0.63	0.54	0.66	0.62	0.70	0.47
$k = 5$	**0.69**	**0.54**	0.63	0.54	0.66	0.62	0.69	0.47
$k = 6$	0.69	0.53	0.63	0.54	0.66	0.62	0.69	0.47
$k = 7$	0.69	0.52	0.63	0.54	0.65	0.62	0.69	0.47
$k = 8$	0.69	0.52	0.63	0.54	0.65	0.62	0.69	0.47
$k = 9$	0.68	0.51	0.63	0.54	0.65	0.62	0.69	0.47
