# Supplementary material for: Effective Harmonic Potentials: Insights into the Internal Cooperativity and Sequence-Specificity of Protein Dynamics
Source: PLoS Comput Biol. 2013 Aug 29;9(8):e1003209. doi: 10.1371/journal.pcbi.1003209 (PMC3757084; doi:10.1371/journal.pcbi.1003209)
Supplement: Table S2 — Spring constants of the . (PDF) [file pcbi.1003209.s009.pdf]

Supporting Table S2: Spring constants of the dENM

Interresidue distance d	$\kappa(d)$
[0.0 , 4.0 [	4.683
[4.0 , 4.5 [	3.897
[4.5 , 5.0 [	3.518
[5.0 , 5.5 [	6.616
[5.5 , 6.0 [	2.024
[6.0 , 6.5 [	1.000
[6.5 , 7.0 [	0.592
[7.0 , 7.5 [	0.067
[7.5 , 8.0 [	0.033
[8.0 , 8.5 [	0.035
[8.5 , 9.0 [	0.052
[9.0 , 9.5 [	0.037
[9.5 , 10.0 [	0.042
[10.0 , 10.5 [	0.034
[10.5 , 11.0 [	0.020
[11.0 , 11.5 [	0.013
[11.5 , 12.0 [	0.010
[12.0 , 12.5 [	0.008
[12.5 , 13.0 [	0.006
[13.0 , 13.5 [	0.005
[13.5 , 14.0 [	0.004
[14.0 , 14.5 [	0.003
[14.5 , 15.0 [	0.002
[15.0 , 15.5 [	0.002
[15.5 , 16.0 [	0.001
[16.0 , 16.5 [	0.001
