# Supplementary material for: Effective Harmonic Potentials: Insights into the Internal Cooperativity and Sequence-Specificity of Protein Dynamics
Source: PLoS Comput Biol. 2013 Aug 29;9(8):e1003209. doi: 10.1371/journal.pcbi.1003209 (PMC3757084; doi:10.1371/journal.pcbi.1003209)
Supplement: Table S3 — Spring constants of the . (PDF) [file pcbi.1003209.s010.pdf]

Supporting Table S3: Spring constants of the $sENM_{10}$

	A	C	D	E	F	G	H	I	K	L	M	N	P	Q	R	S	T	V	W	Y
A	0.885	0.894	0.776	0.851	1.220	0.481	0.647	1.770	0.957	1.500	0.939	0.765	0.577	0.960	0.960	0.678	0.960	1.433	1.361	1.325
C		1.288	0.652	0.777	1.258	0.606	0.660	1.371	0.768	1.233	1.265	0.875	0.733	1.043	0.818	0.596	0.994	1.239	0.980	0.934
D			0.860	0.793	1.063	0.387	0.509	1.319	0.724	1.122	0.721	0.596	0.555	0.758	0.654	0.516	0.690	1.052	0.970	0.858
E				0.766	1.325	0.408	0.655	1.499	0.924	1.590	0.878	0.841	0.597	1.031	0.832	0.666	1.193	1.299	1.278	1.111
F					1.614	0.562	0.791	2.149	1.294	1.693	1.076	1.074	0.759	1.166	1.090	0.889	1.192	2.009	1.910	1.368
G						0.226	0.327	0.756	0.472	0.653	0.392	0.381	0.351	0.381	0.429	0.307	0.429	0.662	0.674	0.617
H							0.628	1.287	0.740	1.056	0.661	0.528	0.493	0.712	0.582	0.430	0.645	1.214	1.044	0.893
I								2.107	1.596	1.989	1.566	1.344	1.163	1.754	1.437	1.216	1.764	2.293	2.259	1.734
K									0.981	1.336	0.941	0.813	0.639	0.853	0.791	0.583	0.815	1.571	1.420	1.199
L										1.611	1.314	1.142	0.857	1.182	1.259	0.914	1.388	1.792	1.748	1.665
M											1.074	0.746	0.546	0.918	0.899	0.691	0.774	1.418	1.186	1.101
N												0.689	0.502	0.684	0.713	0.576	0.771	0.908	0.940	0.931
P													0.523	0.552	0.825	0.428	0.571	0.908	1.026	1.717
Q														0.665	0.825	0.577	0.817	1.158	1.160	1.250
R															0.759	0.559	0.758	1.930	1.186	1.113
S																0.399	0.674	1.039	0.803	0.887
T																	0.901	1.335	1.232	1.301
V																		1.976	1.678	1.884
W																			1.020	1.504
Y																				1.275
