# Supplementary material for: Effective Harmonic Potentials: Insights into the Internal Cooperativity and Sequence-Specificity of Protein Dynamics
Source: PLoS Comput Biol. 2013 Aug 29;9(8):e1003209. doi: 10.1371/journal.pcbi.1003209 (PMC3757084; doi:10.1371/journal.pcbi.1003209)
Supplement: Table S4 — Spring constants of the . (PDF) [file pcbi.1003209.s011.pdf]

Supplementary Table S4: Spring constants of the $sENM_{13}$

	A	C	D	E	F	G	H	I	K	L	M	N	P	Q	R	S	T	V	W	Y
A	0.879	0.860	0.731	0.914	1.528	0.479	0.680	1.592	0.955	1.360	0.995	0.744	0.570	0.839	0.896	0.593	0.918	1.325	1.259	1.289
C		1.570	0.854	0.965	1.411	0.632	0.899	1.566	1.267	1.549	1.299	1.151	0.708	1.054	0.945	0.682	0.940	1.415	1.421	1.245
D			0.621	0.711	1.139	0.404	0.494	1.339	0.739	1.124	0.664	0.615	0.519	0.640	0.612	0.501	0.686	1.126	0.980	0.912
E				0.772	1.352	0.426	0.578	1.518	0.865	1.319	0.822	0.834	0.503	0.779	0.728	0.544	0.917	1.327	1.124	1.177
F					1.676	0.688	0.931	2.213	1.553	1.889	1.286	1.200	0.863	1.287	1.182	1.023	1.332	2.053	1.778	1.564
G						0.258	0.326	0.812	0.505	0.628	0.444	0.425	0.361	0.416	0.487	0.353	0.458	0.698	0.625	0.652
H							0.439	1.330	0.699	0.915	0.601	0.488	0.420	0.646	0.623	0.453	0.672	1.068	0.746	0.993
I								2.184	1.588	1.866	1.569	1.429	1.028	1.505	1.465	1.206	1.644	2.348	1.997	1.794
K									1.006	1.430	1.035	0.825	0.597	0.781	0.810	0.633	0.872	1.372	1.424	1.280
L										1.629	1.170	1.233	0.747	1.208	1.199	0.868	1.330	1.709	1.610	1.714
M											1.103	0.819	0.479	0.753	0.898	0.628	0.779	1.429	1.120	1.166
N												0.771	0.434	0.724	0.634	0.516	0.795	1.052	1.032	1.017
P													0.450	0.496	0.472	0.422	0.567	0.850	0.746	0.852
Q														0.683	0.781	0.542	0.669	1.235	1.175	1.314
R															0.723	0.488	0.735	1.230	1.249	1.060
S																0.368	0.604	0.926	0.744	0.900
T																	0.749	1.276	1.209	1.315
V																		1.992	1.958	1.818
W																			1.285	1.892
Y																				1.519
