# Supplementary material for: Effective Harmonic Potentials: Insights into the Internal Cooperativity and Sequence-Specificity of Protein Dynamics
Source: PLoS Comput Biol. 2013 Aug 29;9(8):e1003209. doi: 10.1371/journal.pcbi.1003209 (PMC3757084; doi:10.1371/journal.pcbi.1003209)
Supplement: Table S5 — Spring constants of the . (PDF) [file pcbi.1003209.s012.pdf]

Supplementary Table S5: Spring constants of the sdENM (1/9)

d	A-A	A-C	A-D	A-E	A-F	A-G	A-H	A-I	A-K	A-L	A-M	A-N
[0.0 , 4.0 [	2.469	3.711	0.988	2.603	4.171	2.606	3.355	10.672	2.846	2.504	1.918	3.098
[4.0 , 4.5 [	3.282	2.792	3.875	3.220	3.105	1.427	3.665	6.772	2.309	5.470	3.272	3.656
[4.5 , 5.0 [	4.118	2.725	2.955	4.221	5.193	0.740	2.099	5.126	3.331	6.010	3.626	2.690
[5.0 , 5.5 [	5.658	4.750	6.661	7.862	6.579	2.462	5.686	8.975	6.730	6.935	6.484	4.491
[5.5 , 6.0 [	1.786	2.170	1.387	1.922	2.377	0.723	1.144	2.524	1.600	2.333	1.974	1.640
[6.0 , 6.5 [	1.155	0.654	0.541	0.778	0.923	0.397	0.797	1.511	0.703	1.166	0.520	0.436
[6.5 , 7.0 [	0.122	0.658	0.427	0.425	0.835	0.430	0.322	1.146	0.560	0.595	0.487	0.350
[7.0 , 7.5 [	0.018	0.031	0.010	0.023	0.084	0.007	0.009	0.133	0.036	0.101	0.024	0.019
[7.5 , 8.0 [	0.008	0.024	0.011	0.008	0.061	0.006	0.014	0.081	0.028	0.042	0.054	0.008
[8.0 , 8.5 [	0.010	0.026	0.010	0.011	0.017	0.009	0.008	0.061	0.012	0.050	0.039	0.012
[8.5 , 9.0 [	0.022	0.015	0.022	0.025	0.088	0.017	0.035	0.059	0.017	0.060	0.015	0.016
[9.0 , 9.5 [	0.012	0.019	0.016	0.014	0.033	0.010	0.009	0.029	0.020	0.034	0.007	0.025
[9.5 , 10.0 [	0.019	0.021	0.013	0.012	0.036	0.008	0.016	0.065	0.027	0.041	0.028	0.015
[10.0 , 10.5 [	0.016	0.014	0.012	0.017	0.060	0.009	0.020	0.047	0.020	0.037	0.020	0.010
[10.5 , 11.0 [	0.006	0.007	0.011	0.012	0.019	0.004	0.012	0.014	0.013	0.017	0.016	0.008
[11.0 , 11.5 [	0.004	0.004	0.004	0.007	0.017	0.003	0.006	0.019	0.009	0.010	0.010	0.006
[11.5 , 12.0 [	0.005	0.008	0.004	0.006	0.013	0.003	0.002	0.009	0.004	0.007	0.006	0.004
[12.0 , 12.5 [	0.002	0.003	0.003	0.004	0.008	0.002	0.007	0.008	0.003	0.005	0.006	0.003
[12.5 , 13.0 [	0.002	0.003	0.002	0.003	0.009	0.002	0.003	0.005	0.002	0.005	0.004	0.003
[13.0 , 13.5 [	0.001	0.008	0.002	0.002	0.005	0.001	0.001	0.005	0.004	0.004	0.002	0.002
[13.5 , 14.0 [	0.001	0.001	0.002	0.002	0.003	0.001	0.001	0.003	0.002	0.002	0.001	0.002
[14.0 , 14.5 [	0.001	0.002	0.001	0.001	0.003	0.001	0.001	0.002	0.002	0.002	0.002	0.001
[14.5 , 15.0 [	0.001	0.001	0.001	0.001	0.001	0.001	0.001	0.001	0.001	0.001	0.001	0.001
[15.0 , 15.5 [	0.001	0.001	0.001	0.001	0.001	0.001	0.001	0.001	0.001	0.001	0.001	0.001
[15.5 , 16.0 [	0.001	0.001	0.001	0.001	0.001	0.001	0.001	0.001	0.001	0.001	0.001	0.001
[16.0 , 16.5 [	0.001	0.001	0.001	0.001	0.001	0.001	0.001	0.001	0.001	0.001	0.001	0.001

d	A-P	A-Q	A-R	A-S	A-T	A-V	A-W	A-Y	C-C	C-D	C-E	C-F
[0.0 , 4.0 [	7.593	2.357	4.226	6.504	5.609	7.944	4.095	7.859	10.227	3.320	2.923	4.599
[4.0 , 4.5 [	1.799	1.593	2.008	5.365	2.576	5.932	4.853	5.716	1.944	3.478	2.543	4.895
[4.5 , 5.0 [	2.210	2.965	2.842	3.189	2.616	5.077	3.836	3.255	2.101	1.239	3.520	3.951
[5.0 , 5.5 [	3.054	7.316	5.680	3.569	5.907	6.676	9.903	5.657	2.021	3.957	5.680	7.726
[5.5 , 6.0 [	1.628	1.627	1.747	1.063	2.037	2.658	1.362	2.512	1.400	1.079	2.018	1.592
[6.0 , 6.5 [	0.700	0.425	0.637	0.574	0.540	1.205	0.802	1.222	1.672	0.754	0.620	2.005
[6.5 , 7.0 [	0.582	0.499	0.662	0.231	0.498	1.073	0.448	1.066	0.540	0.522	0.553	0.881
[7.0 , 7.5 [	0.025	0.006	0.036	0.017	0.030	0.135	0.021	0.051	0.065	0.016	0.039	0.122
[7.5 , 8.0 [	0.020	0.019	0.007	0.011	0.019	0.022	0.022	0.026	0.029	0.021	0.008	0.019
[8.0 , 8.5 [	0.018	0.012	0.018	0.008	0.016	0.021	0.036	0.023	0.049	0.013	0.035	0.018
[8.5 , 9.0 [	0.024	0.015	0.025	0.024	0.022	0.044	0.063	0.051	0.111	0.034	0.013	0.053
[9.0 , 9.5 [	0.023	0.016	0.021	0.009	0.019	0.030	0.041	0.042	0.020	0.013	0.014	0.074
[9.5 , 10.0 [	0.020	0.024	0.028	0.009	0.027	0.031	0.042	0.039	0.050	0.016	0.042	0.032
[10.0 , 10.5 [	0.015	0.014	0.007	0.008	0.024	0.028	0.048	0.037	0.013	0.031	0.012	0.053
[10.5 , 11.0 [	0.008	0.005	0.007	0.008	0.012	0.016	0.004	0.014	0.003	0.010	0.013	0.039
[11.0 , 11.5 [	0.004	0.006	0.009	0.003	0.009	0.007	0.014	0.012	0.003	0.006	0.008	0.020
[11.5 , 12.0 [	0.005	0.004	0.003	0.002	0.005	0.009	0.004	0.010	0.008	0.005	0.005	0.005
[12.0 , 12.5 [	0.004	0.003	0.004	0.003	0.003	0.006	0.014	0.005	0.007	0.003	0.005	0.004
[12.5 , 13.0 [	0.002	0.002	0.003	0.001	0.003	0.004	0.003	0.005	0.006	0.002	0.005	0.011
[13.0 , 13.5 [	0.002	0.002	0.002	0.001	0.003	0.002	0.003	0.003	0.003	0.001	0.003	0.005
[13.5 , 14.0 [	0.001	0.001	0.001	0.001	0.001	0.003	0.002	0.002	0.002	0.001	0.001	0.003
[14.0 , 14.5 [	0.001	0.001	0.001	0.001	0.001	0.001	0.001	0.003	0.002	0.001	0.001	0.002
[14.5 , 15.0 [	0.001	0.001	0.001	0.001	0.001	0.001	0.001	0.001	0.001	0.001	0.001	0.001
[15.0 , 15.5 [	0.001	0.001	0.001	0.001	0.001	0.001	0.001	0.001	0.001	0.002	0.001	0.002
[15.5 , 16.0 [	0.001	0.001	0.001	0.001	0.001	0.001	0.001	0.001	0.001	0.001	0.001	0.001
[16.0 , 16.5 [	0.001	0.001	0.001	0.001	0.001	0.001	0.001	0.001	0.001	0.001	0.001	0.001

Supplementary Table S5: Spring constants of the sdENM (2/9)

d	C-G	C-H	C-I	C-K	C-L	C-M	C-N	C-P	C-Q	C-R	C-S	C-T
[0.0 , 4.0 [	4.914	3.320	4.565	3.963	4.578	3.320	3.932	4.060	4.028	2.762	3.892	4.579
[4.0 , 4.5 [	1.044	2.818	5.794	4.722	3.970	3.742	1.612	4.837	2.786	5.512	3.835	2.823
[4.5 , 5.0 [	0.827	4.616	4.672	2.687	2.002	3.062	2.959	1.255	3.633	2.403	3.090	2.201
[5.0 , 5.5 [	1.811	4.587	7.789	5.848	5.149	2.315	4.150	3.566	8.958	3.946	3.599	2.994
[5.5 , 6.0 [	0.805	0.910	1.761	1.982	2.197	2.781	1.215	0.890	1.076	1.100	1.314	1.727
[6.0 , 6.5 [	0.688	0.778	1.308	0.529	1.212	0.678	1.291	1.804	0.656	1.094	0.403	0.934
[6.5 , 7.0 [	0.272	1.009	0.874	0.547	0.636	0.662	0.233	0.448	0.689	0.451	0.291	0.925
[7.0 , 7.5 [	0.027	0.021	0.104	0.085	0.183	0.236	0.054	0.047	0.053	0.076	0.022	0.034
[7.5 , 8.0 [	0.008	0.018	0.021	0.013	0.071	0.019	0.020	0.035	0.016	0.024	0.037	0.028
[8.0 , 8.5 [	0.022	0.035	0.090	0.018	0.068	0.016	0.029	0.019	0.014	0.005	0.006	0.036
[8.5 , 9.0 [	0.017	0.082	0.075	0.025	0.056	0.066	0.043	0.053	0.048	0.016	0.033	0.033
[9.0 , 9.5 [	0.009	0.019	0.086	0.014	0.029	0.057	0.024	0.011	0.025	0.084	0.006	0.017
[9.5 , 10.0 [	0.027	0.011	0.037	0.011	0.041	0.049	0.046	0.031	0.031	0.012	0.017	0.024
[10.0 , 10.5 [	0.014	0.036	0.026	0.034	0.047	0.021	0.018	0.014	0.022	0.027	0.009	0.031
[10.5 , 11.0 [	0.014	0.028	0.043	0.042	0.018	0.027	0.007	0.013	0.011	0.014	0.009	0.011
[11.0 , 11.5 [	0.002	0.024	0.020	0.007	0.015	0.009	0.007	0.004	0.009	0.013	0.005	0.004
[11.5 , 12.0 [	0.008	0.007	0.005	0.014	0.006	0.007	0.011	0.002	0.010	0.012	0.004	0.005
[12.0 , 12.5 [	0.002	0.004	0.007	0.008	0.013	0.005	0.013	0.004	0.004	0.004	0.003	0.003
[12.5 , 13.0 [	0.002	0.006	0.010	0.008	0.009	0.010	0.005	0.009	0.004	0.001	0.001	0.001
[13.0 , 13.5 [	0.001	0.005	0.003	0.002	0.009	0.004	0.002	0.002	0.008	0.001	0.002	0.002
[13.5 , 14.0 [	0.001	0.002	0.004	0.003	0.002	0.002	0.002	0.003	0.001	0.001	0.003	0.001
[14.0 , 14.5 [	0.001	0.001	0.001	0.001	0.003	0.003	0.001	0.002	0.001	0.001	0.001	0.002
[14.5 , 15.0 [	0.001	0.001	0.002	0.001	0.001	0.002	0.002	0.001	0.002	0.001	0.001	0.001
[15.0 , 15.5 [	0.001	0.002	0.001	0.001	0.002	0.001	0.002	0.001	0.001	0.001	0.001	0.001
[15.5 , 16.0 [	0.001	0.001	0.001	0.001	0.001	0.002	0.001	0.001	0.001	0.001	0.001	0.001
[16.0 , 16.5 [	0.001	0.001	0.001	0.001	0.001	0.001	0.001	0.001	0.001	0.001	0.001	0.001

d	C-V	C-W	C-Y	D-D	D-E	D-F	D-G	D-H	D-I	D-K	D-L	D-M
[0.0 , 4.0 [	1.784	3.901	6.127	3.535	2.786	3.012	0.539	1.973	4.736	5.772	1.814	2.328
[4.0 , 4.5 [	3.307	3.024	4.661	2.025	1.991	4.525	0.761	2.793	2.531	6.041	1.442	1.888
[4.5 , 5.0 [	2.495	2.830	3.813	2.393	1.410	1.550	1.801	1.508	5.710	2.307	4.113	2.536
[5.0 , 5.5 [	4.113	7.035	3.417	5.885	7.961	7.165	2.404	7.269	7.526	5.887	9.356	9.082
[5.5 , 6.0 [	2.103	1.296	0.816	1.607	2.201	1.838	1.009	0.845	3.467	1.690	1.308	0.735
[6.0 , 6.5 [	2.037	1.224	1.119	0.702	0.528	1.030	0.300	1.080	1.177	1.297	1.095	0.334
[6.5 , 7.0 [	0.682	0.432	0.150	0.382	0.638	0.853	0.279	0.171	0.794	0.247	0.778	0.639
[7.0 , 7.5 [	0.072	0.022	0.060	0.020	0.048	0.053	0.012	0.046	0.053	0.031	0.052	0.073
[7.5 , 8.0 [	0.014	0.056	0.032	0.008	0.011	0.021	0.005	0.011	0.030	0.015	0.043	0.041
[8.0 , 8.5 [	0.106	0.043	0.043	0.012	0.018	0.036	0.010	0.009	0.018	0.018	0.022	0.018
[8.5 , 9.0 [	0.084	0.362	0.074	0.015	0.034	0.041	0.014	0.094	0.026	0.032	0.044	0.020
[9.0 , 9.5 [	0.026	0.016	0.020	0.009	0.020	0.032	0.012	0.010	0.042	0.029	0.020	0.023
[9.5 , 10.0 [	0.038	0.061	0.030	0.019	0.014	0.052	0.012	0.015	0.045	0.017	0.029	0.014
[10.0 , 10.5 [	0.090	0.058	0.051	0.016	0.017	0.034	0.008	0.009	0.038	0.011	0.041	0.021
[10.5 , 11.0 [	0.011	0.016	0.028	0.009	0.009	0.015	0.003	0.006	0.036	0.010	0.013	0.007
[11.0 , 11.5 [	0.016	0.013	0.024	0.004	0.006	0.011	0.005	0.005	0.006	0.005	0.013	0.012
[11.5 , 12.0 [	0.011	0.006	0.010	0.003	0.004	0.007	0.002	0.004	0.010	0.005	0.007	0.003
[12.0 , 12.5 [	0.006	0.005	0.005	0.003	0.004	0.006	0.002	0.002	0.009	0.004	0.006	0.003
[12.5 , 13.0 [	0.007	0.003	0.003	0.003	0.003	0.004	0.002	0.003	0.008	0.003	0.005	0.004
[13.0 , 13.5 [	0.006	0.007	0.002	0.002	0.002	0.003	0.002	0.003	0.003	0.003	0.003	0.004
[13.5 , 14.0 [	0.002	0.004	0.003	0.001	0.002	0.002	0.001	0.002	0.003	0.002	0.003	0.003
[14.0 , 14.5 [	0.002	0.003	0.001	0.001	0.001	0.002	0.001	0.002	0.002	0.001	0.001	0.002
[14.5 , 15.0 [	0.001	0.001	0.001	0.001	0.001	0.002	0.001	0.001	0.001	0.001	0.002	0.001
[15.0 , 15.5 [	0.002	0.005	0.002	0.001	0.001	0.001	0.001	0.001	0.001	0.001	0.001	0.001
[15.5 , 16.0 [	0.001	0.001	0.001	0.001	0.001	0.001	0.001	0.001	0.001	0.001	0.001	0.001
[16.0 , 16.5 [	0.001	0.001	0.001	0.001	0.001	0.001	0.001	0.001	0.001	0.001	0.001	0.001

Supplementary Table S5: Spring constants of the sdENM (3/9)

d	D-N	D-P	D-Q	D-R	D-S	D-T	D-V	D-W	D-Y	E-E	E-F	E-G
[0.0 , 4.0 [	3.815	3.551	1.796	3.016	8.843	2.355	3.573	3.320	4.065	5.286	4.165	4.341
[4.0 , 4.5 [	5.903	2.251	1.701	6.673	0.780	3.707	1.828	5.007	2.340	4.250	3.288	2.120
[4.5 , 5.0 [	1.563	1.095	5.486	1.913	1.854	1.493	5.280	4.917	2.136	4.458	4.879	1.386
[5.0 , 5.5 [	5.940	3.669	4.026	5.895	4.061	7.778	7.728	7.011	6.134	8.361	6.710	3.122
[5.5 , 6.0 [	1.236	1.126	1.272	1.455	1.639	1.649	2.673	1.204	1.213	1.474	2.058	0.940
[6.0 , 6.5 [	0.508	0.949	0.538	0.367	0.374	0.490	0.719	1.105	0.864	0.890	1.233	0.391
[6.5 , 7.0 [	0.389	0.766	0.636	0.316	0.411	0.455	0.878	1.021	0.725	0.565	0.607	0.412
[7.0 , 7.5 [	0.033	0.040	0.056	0.026	0.017	0.020	0.114	0.031	0.050	0.051	0.038	0.014
[7.5 , 8.0 [	0.008	0.014	0.027	0.008	0.006	0.011	0.016	0.013	0.013	0.014	0.027	0.009
[8.0 , 8.5 [	0.009	0.025	0.022	0.012	0.019	0.008	0.019	0.049	0.035	0.010	0.034	0.005
[8.5 , 9.0 [	0.024	0.022	0.035	0.021	0.014	0.044	0.038	0.026	0.015	0.030	0.060	0.012
[9.0 , 9.5 [	0.016	0.013	0.012	0.015	0.015	0.029	0.018	0.045	0.026	0.014	0.046	0.008
[9.5 , 10.0 [	0.014	0.018	0.025	0.020	0.009	0.019	0.027	0.039	0.038	0.026	0.048	0.011
[10.0 , 10.5 [	0.013	0.012	0.019	0.018	0.013	0.020	0.041	0.050	0.026	0.014	0.040	0.007
[10.5 , 11.0 [	0.009	0.005	0.008	0.005	0.011	0.012	0.019	0.020	0.012	0.011	0.018	0.004
[11.0 , 11.5 [	0.008	0.006	0.003	0.005	0.002	0.006	0.010	0.012	0.011	0.004	0.016	0.004
[11.5 , 12.0 [	0.004	0.007	0.004	0.003	0.002	0.004	0.006	0.011	0.006	0.007	0.008	0.002
[12.0 , 12.5 [	0.002	0.005	0.003	0.003	0.002	0.002	0.008	0.009	0.006	0.002	0.010	0.004
[12.5 , 13.0 [	0.002	0.003	0.003	0.003	0.003	0.002	0.004	0.002	0.003	0.003	0.005	0.002
[13.0 , 13.5 [	0.002	0.001	0.002	0.001	0.001	0.003	0.003	0.001	0.003	0.002	0.005	0.002
[13.5 , 14.0 [	0.002	0.002	0.002	0.001	0.001	0.002	0.003	0.002	0.002	0.002	0.005	0.001
[14.0 , 14.5 [	0.001	0.001	0.001	0.001	0.001	0.001	0.002	0.002	0.001	0.001	0.002	0.001
[14.5 , 15.0 [	0.001	0.001	0.001	0.001	0.001	0.001	0.001	0.001	0.001	0.001	0.002	0.001
[15.0 , 15.5 [	0.001	0.001	0.001	0.001	0.001	0.001	0.001	0.001	0.001	0.001	0.001	0.001
[15.5 , 16.0 [	0.001	0.001	0.001	0.001	0.001	0.001	0.001	0.001	0.001	0.001	0.001	0.001
[16.0 , 16.5 [	0.001	0.001	0.001	0.001	0.001	0.001	0.001	0.001	0.001	0.001	0.001	0.001

d	E-H	E-I	E-K	E-L	E-M	E-N	E-P	E-Q	E-R	E-S	E-T	E-V
[0.0 , 4.0 [	6.281	6.008	11.864	4.973	6.193	3.433	5.236	4.318	5.191	2.937	3.015	7.836
[4.0 , 4.5 [	4.798	3.881	4.499	2.482	3.224	2.104	6.071	2.826	5.611	5.205	4.321	3.520
[4.5 , 5.0 [	3.276	3.403	3.321	3.170	3.402	3.880	1.161	4.076	2.936	3.888	2.469	3.153
[5.0 , 5.5 [	5.468	7.108	5.628	7.128	4.787	5.798	4.924	4.869	4.445	5.033	8.669	7.258
[5.5 , 6.0 [	0.880	3.260	1.653	2.598	1.842	1.815	1.144	2.250	1.694	1.975	1.753	2.173
[6.0 , 6.5 [	0.767	1.618	0.771	0.948	0.544	1.496	0.745	1.215	1.199	0.386	0.838	1.481
[6.5 , 7.0 [	0.528	0.788	0.874	0.687	0.489	0.541	0.566	0.404	0.445	0.449	1.216	0.849
[7.0 , 7.5 [	0.012	0.147	0.066	0.095	0.025	0.029	0.035	0.017	0.059	0.022	0.033	0.079
[7.5 , 8.0 [	0.016	0.029	0.026	0.046	0.011	0.010	0.021	0.017	0.019	0.008	0.022	0.026
[8.0 , 8.5 [	0.011	0.031	0.018	0.035	0.016	0.015	0.023	0.014	0.015	0.010	0.014	0.011
[8.5 , 9.0 [	0.025	0.031	0.026	0.059	0.029	0.042	0.021	0.028	0.018	0.018	0.023	0.063
[9.0 , 9.5 [	0.016	0.056	0.023	0.031	0.053	0.023	0.020	0.016	0.025	0.017	0.014	0.033
[9.5 , 10.0 [	0.027	0.046	0.031	0.035	0.024	0.031	0.024	0.027	0.017	0.014	0.026	0.030
[10.0 , 10.5 [	0.013	0.043	0.033	0.040	0.013	0.034	0.011	0.015	0.015	0.008	0.024	0.034
[10.5 , 11.0 [	0.005	0.032	0.008	0.015	0.010	0.019	0.010	0.013	0.012	0.007	0.011	0.015
[11.0 , 11.5 [	0.004	0.020	0.004	0.012	0.006	0.009	0.003	0.009	0.005	0.005	0.007	0.011
[11.5 , 12.0 [	0.002	0.012	0.004	0.007	0.004	0.006	0.005	0.003	0.005	0.003	0.006	0.008
[12.0 , 12.5 [	0.003	0.009	0.004	0.006	0.004	0.005	0.003	0.003	0.002	0.002	0.004	0.008
[12.5 , 13.0 [	0.001	0.005	0.003	0.006	0.006	0.003	0.003	0.002	0.002	0.002	0.004	0.004
[13.0 , 13.5 [	0.002	0.004	0.003	0.004	0.002	0.002	0.002	0.002	0.001	0.002	0.003	0.004
[13.5 , 14.0 [	0.002	0.003	0.002	0.002	0.002	0.002	0.002	0.002	0.002	0.001	0.002	0.003
[14.0 , 14.5 [	0.001	0.002	0.001	0.002	0.002	0.001	0.001	0.001	0.001	0.001	0.001	0.002
[14.5 , 15.0 [	0.001	0.001	0.001	0.001	0.002	0.001	0.001	0.001	0.001	0.001	0.001	0.001
[15.0 , 15.5 [	0.001	0.001	0.001	0.001	0.002	0.001	0.001	0.001	0.001	0.001	0.001	0.001
[15.5 , 16.0 [	0.001	0.001	0.001	0.001	0.001	0.001	0.001	0.001	0.001	0.001	0.001	0.001
[16.0 , 16.5 [	0.001	0.001	0.001	0.001	0.001	0.001	0.001	0.001	0.001	0.001	0.001	0.001

Supplementary Table S5: Spring constants of the sdENM (4/9)

d	E-W	E-Y	F-F	F-G	F-H	F-I	F-K	F-L	F-M	F-N	F-P	F-Q
[0.0 , 4.0 [	3.320	4.800	4.874	13.043	5.123	3.849	9.110	5.574	3.363	5.853	5.058	2.982
[4.0 , 4.5 [	3.226	6.801	3.846	2.743	2.851	4.039	1.113	3.491	2.662	5.007	5.172	4.186
[4.5 , 5.0 [	3.275	4.855	2.177	0.743	1.816	5.767	1.950	7.642	3.515	3.772	1.478	3.144
[5.0 , 5.5 [	7.138	6.108	8.528	3.270	2.733	3.874	8.433	4.435	5.114	5.664	4.104	6.276
[5.5 , 6.0 [	2.185	2.810	2.094	0.951	1.664	3.500	1.594	2.253	1.327	2.819	1.863	2.557
[6.0 , 6.5 [	0.736	1.236	1.608	0.574	0.964	2.351	0.846	2.925	0.845	0.596	2.834	0.754
[6.5 , 7.0 [	0.894	0.728	1.746	0.248	0.568	1.769	0.893	1.206	0.985	0.907	0.341	0.562
[7.0 , 7.5 [	0.045	0.068	0.135	0.024	0.088	0.396	0.050	0.247	0.021	0.121	0.082	0.079
[7.5 , 8.0 [	0.018	0.017	0.082	0.012	0.005	0.126	0.053	0.090	0.020	0.040	0.027	0.034
[8.0 , 8.5 [	0.046	0.037	0.081	0.011	0.043	0.146	0.068	0.111	0.081	0.098	0.026	0.021
[8.5 , 9.0 [	0.096	0.048	0.164	0.019	0.028	0.173	0.067	0.156	0.045	0.028	0.048	0.039
[9.0 , 9.5 [	0.052	0.034	0.070	0.010	0.017	0.086	0.046	0.083	0.064	0.048	0.070	0.050
[9.5 , 10.0 [	0.034	0.057	0.119	0.020	0.021	0.164	0.061	0.101	0.072	0.039	0.024	0.041
[10.0 , 10.5 [	0.040	0.045	0.061	0.018	0.034	0.115	0.054	0.076	0.042	0.044	0.026	0.046
[10.5 , 11.0 [	0.007	0.028	0.074	0.007	0.049	0.060	0.031	0.056	0.033	0.017	0.020	0.025
[11.0 , 11.5 [	0.019	0.009	0.027	0.011	0.010	0.043	0.016	0.036	0.029	0.013	0.010	0.011
[11.5 , 12.0 [	0.004	0.007	0.011	0.005	0.003	0.019	0.020	0.017	0.008	0.007	0.020	0.008
[12.0 , 12.5 [	0.006	0.006	0.015	0.005	0.004	0.020	0.013	0.014	0.006	0.007	0.006	0.009
[12.5 , 13.0 [	0.004	0.007	0.013	0.003	0.005	0.011	0.008	0.016	0.005	0.013	0.003	0.006
[13.0 , 13.5 [	0.005	0.005	0.006	0.002	0.002	0.010	0.006	0.008	0.007	0.008	0.003	0.005
[13.5 , 14.0 [	0.003	0.003	0.005	0.002	0.002	0.008	0.005	0.007	0.009	0.002	0.002	0.004
[14.0 , 14.5 [	0.001	0.002	0.001	0.002	0.001	0.004	0.003	0.003	0.006	0.005	0.001	0.002
[14.5 , 15.0 [	0.001	0.001	0.006	0.001	0.001	0.002	0.002	0.002	0.003	0.001	0.001	0.001
[15.0 , 15.5 [	0.001	0.001	0.002	0.001	0.001	0.003	0.001	0.001	0.001	0.002	0.001	0.001
[15.5 , 16.0 [	0.001	0.001	0.001	0.001	0.001	0.002	0.001	0.001	0.001	0.001	0.001	0.001
[16.0 , 16.5 [	0.001	0.001	0.001	0.001	0.001	0.001	0.001	0.001	0.001	0.001	0.001	0.001
d	F-R	F-S	F-T	F-V	F-W	F-Y	G-G	G-H	G-I	G-K	G-L	G-M
[0.0 , 4.0 [	4.374	3.879	3.815	6.759	6.521	2.812	1.084	5.126	2.756	1.860	3.939	3.406
[4.0 , 4.5 [	2.288	4.786	1.925	4.260	3.933	8.847	1.581	1.863	3.279	0.619	2.180	3.095
[4.5 , 5.0 [	3.917	2.907	5.597	3.870	6.508	2.324	0.455	0.914	2.038	1.325	1.957	1.351
[5.0 , 5.5 [	5.115	4.569	5.953	8.232	3.712	4.890	0.754	1.755	7.350	2.878	3.249	3.780
[5.5 , 6.0 [	1.905	2.320	2.298	3.067	1.840	1.785	0.315	0.521	2.554	1.115	0.788	0.516
[6.0 , 6.5 [	0.582	1.083	1.200	1.812	1.438	0.951	0.379	0.143	0.278	0.392	0.462	0.866
[6.5 , 7.0 [	0.724	0.390	1.422	1.877	0.771	1.452	0.150	0.252	0.352	0.365	0.404	0.133
[7.0 , 7.5 [	0.066	0.027	0.059	0.193	0.326	0.069	0.003	0.005	0.034	0.013	0.039	0.010
[7.5 , 8.0 [	0.035	0.042	0.045	0.086	0.074	0.071	0.004	0.003	0.006	0.011	0.011	0.006
[8.0 , 8.5 [	0.029	0.035	0.023	0.080	0.109	0.039	0.002	0.013	0.018	0.007	0.007	0.005
[8.5 , 9.0 [	0.049	0.028	0.070	0.105	0.146	0.077	0.010	0.040	0.013	0.014	0.017	0.015
[9.0 , 9.5 [	0.025	0.025	0.026	0.075	0.053	0.085	0.010	0.012	0.018	0.023	0.027	0.008
[9.5 , 10.0 [	0.030	0.031	0.042	0.119	0.215	0.074	0.012	0.014	0.022	0.009	0.018	0.016
[10.0 , 10.5 [	0.024	0.016	0.044	0.060	0.052	0.063	0.012	0.008	0.019	0.014	0.014	0.005
[10.5 , 11.0 [	0.019	0.018	0.018	0.028	0.043	0.055	0.002	0.004	0.009	0.008	0.009	0.003
[11.0 , 11.5 [	0.009	0.011	0.014	0.035	0.030	0.018	0.003	0.003	0.005	0.004	0.004	0.004
[11.5 , 12.0 [	0.008	0.011	0.005	0.033	0.016	0.014	0.001	0.003	0.006	0.004	0.004	0.003
[12.0 , 12.5 [	0.010	0.006	0.016	0.018	0.009	0.008	0.002	0.003	0.005	0.002	0.003	0.002
[12.5 , 13.0 [	0.004	0.004	0.005	0.009	0.007	0.012	0.001	0.001	0.003	0.002	0.002	0.003
[13.0 , 13.5 [	0.003	0.004	0.006	0.007	0.006	0.010	0.001	0.002	0.003	0.001	0.002	0.001
[13.5 , 14.0 [	0.003	0.001	0.006	0.004	0.002	0.005	0.001	0.002	0.002	0.001	0.002	0.001
[14.0 , 14.5 [	0.004	0.001	0.002	0.002	0.003	0.004	0.001	0.001	0.002	0.001	0.001	0.001
[14.5 , 15.0 [	0.001	0.002	0.001	0.002	0.003	0.002	0.001	0.001	0.001	0.001	0.001	0.001
[15.0 , 15.5 [	0.001	0.001	0.001	0.001	0.002	0.002	0.001	0.001	0.001	0.001	0.001	0.001
[15.5 , 16.0 [	0.001	0.001	0.001	0.001	0.001	0.001	0.001	0.001	0.001	0.001	0.001	0.001
[16.0 , 16.5 [	0.001	0.001	0.001	0.001	0.001	0.001	0.001	0.001	0.001	0.001	0.001	0.001

Supplementary Table S5: Spring constants of the sdENM (5/9)

d	G-N	G-P	G-Q	G-R	G-S	G-T	G-V	G-W	G-Y	H-H	H-I	H-K
[0.0 , 4.0 [	2.683	4.593	4.498	1.654	3.034	3.043	6.660	4.177	5.907	3.320	3.320	4.006
[4.0 , 4.5 [	1.874	1.380	1.323	1.671	1.019	1.447	2.394	0.816	0.930	3.125	4.965	1.589
[4.5 , 5.0 [	1.259	0.561	1.085	0.963	0.674	2.242	1.022	1.176	1.278	2.595	2.846	4.667
[5.0 , 5.5 [	2.885	1.466	1.940	2.506	1.807	2.314	3.701	1.691	2.657	3.964	6.440	3.393
[5.5 , 6.0 [	0.637	0.792	1.161	0.603	0.626	0.880	1.512	1.138	0.921	1.178	2.788	2.412
[6.0 , 6.5 [	0.443	0.614	0.299	0.304	0.619	0.314	0.746	0.686	0.672	1.953	1.224	0.836
[6.5 , 7.0 [	0.348	0.554	0.261	0.418	0.402	0.590	0.246	0.267	0.414	0.878	0.459	0.294
[7.0 , 7.5 [	0.008	0.028	0.010	0.013	0.013	0.007	0.023	0.035	0.021	0.050	0.169	0.033
[7.5 , 8.0 [	0.016	0.014	0.007	0.007	0.007	0.008	0.009	0.061	0.012	0.010	0.035	0.023
[8.0 , 8.5 [	0.009	0.011	0.006	0.008	0.004	0.004	0.009	0.017	0.021	0.069	0.063	0.022
[8.5 , 9.0 [	0.017	0.032	0.007	0.020	0.009	0.011	0.016	0.031	0.014	0.019	0.039	0.030
[9.0 , 9.5 [	0.008	0.011	0.004	0.010	0.007	0.009	0.011	0.020	0.028	0.017	0.016	0.010
[9.5 , 10.0 [	0.008	0.011	0.005	0.009	0.007	0.022	0.021	0.038	0.043	0.027	0.067	0.014
[10.0 , 10.5 [	0.006	0.007	0.004	0.013	0.007	0.011	0.012	0.010	0.011	0.016	0.023	0.012
[10.5 , 11.0 [	0.011	0.007	0.006	0.003	0.003	0.010	0.008	0.008	0.009	0.043	0.026	0.010
[11.0 , 11.5 [	0.003	0.003	0.003	0.006	0.004	0.002	0.006	0.004	0.005	0.008	0.027	0.005
[11.5 , 12.0 [	0.003	0.003	0.002	0.003	0.002	0.002	0.002	0.002	0.011	0.006	0.004	0.006
[12.0 , 12.5 [	0.001	0.002	0.002	0.003	0.002	0.002	0.003	0.009	0.002	0.003	0.006	0.003
[12.5 , 13.0 [	0.003	0.003	0.001	0.002	0.002	0.001	0.002	0.002	0.003	0.001	0.004	0.003
[13.0 , 13.5 [	0.001	0.002	0.002	0.001	0.001	0.002	0.001	0.001	0.003	0.002	0.002	0.002
[13.5 , 14.0 [	0.001	0.002	0.002	0.001	0.001	0.001	0.001	0.002	0.001	0.001	0.001	0.002
[14.0 , 14.5 [	0.002	0.001	0.001	0.001	0.001	0.001	0.001	0.001	0.001	0.001	0.002	0.001
[14.5 , 15.0 [	0.001	0.001	0.001	0.001	0.001	0.001	0.001	0.001	0.001	0.001	0.001	0.001
[15.0 , 15.5 [	0.001	0.002	0.001	0.001	0.001	0.001	0.001	0.001	0.001	0.001	0.001	0.001
[15.5 , 16.0 [	0.001	0.001	0.001	0.001	0.001	0.001	0.001	0.001	0.001	0.001	0.001	0.001
[16.0 , 16.5 [	0.001	0.001	0.001	0.001	0.001	0.001	0.001	0.001	0.001	0.001	0.001	0.001
d	H-L	H-M	H-N	H-P	H-Q	H-R	H-S	H-T	H-V	H-W	H-Y	I-I
[0.0 , 4.0 [	3.796	3.320	2.125	4.458	4.047	3.710	4.482	3.726	6.959	3.320	3.840	5.039
[4.0 , 4.5 [	2.180	3.455	1.484	1.905	3.842	2.845	5.933	4.190	3.247	1.754	4.413	10.864
[4.5 , 5.0 [	4.626	1.060	4.271	1.905	2.670	2.034	1.552	2.882	3.941	4.798	2.765	7.415
[5.0 , 5.5 [	4.760	6.038	6.394	3.566	2.698	7.674	4.255	5.441	6.489	3.050	4.883	6.505
[5.5 , 6.0 [	2.010	2.052	0.995	1.802	0.796	1.070	0.578	1.010	3.070	1.356	1.481	3.291
[6.0 , 6.5 [	0.955	0.228	0.470	0.563	1.202	0.905	0.485	0.570	1.089	0.703	0.715	2.327
[6.5 , 7.0 [	0.307	0.313	1.221	1.358	0.283	0.428	0.239	1.146	0.869	1.640	0.721	1.972
[7.0 , 7.5 [	0.022	0.036	0.015	0.027	0.053	0.021	0.067	0.026	0.104	0.042	0.137	0.230
[7.5 , 8.0 [	0.029	0.007	0.009	0.008	0.011	0.002	0.005	0.017	0.045	0.023	0.027	0.122
[8.0 , 8.5 [	0.040	0.017	0.013	0.025	0.014	0.012	0.005	0.029	0.021	0.009	0.039	0.095
[8.5 , 9.0 [	0.046	0.034	0.037	0.024	0.052	0.012	0.014	0.068	0.034	0.070	0.024	0.190
[9.0 , 9.5 [	0.023	0.022	0.007	0.025	0.017	0.027	0.005	0.014	0.025	0.035	0.038	0.103
[9.5 , 10.0 [	0.031	0.017	0.011	0.007	0.025	0.016	0.017	0.012	0.022	0.016	0.020	0.112
[10.0 , 10.5 [	0.027	0.012	0.021	0.008	0.013	0.013	0.017	0.014	0.040	0.018	0.021	0.050
[10.5 , 11.0 [	0.015	0.002	0.006	0.004	0.009	0.008	0.005	0.025	0.013	0.007	0.026	0.039
[11.0 , 11.5 [	0.007	0.005	0.002	0.008	0.004	0.008	0.004	0.004	0.011	0.004	0.010	0.030
[11.5 , 12.0 [	0.009	0.007	0.004	0.004	0.004	0.002	0.002	0.005	0.008	0.003	0.009	0.016
[12.0 , 12.5 [	0.007	0.015	0.001	0.004	0.004	0.003	0.001	0.003	0.005	0.017	0.005	0.018
[12.5 , 13.0 [	0.003	0.002	0.002	0.003	0.004	0.002	0.002	0.003	0.004	0.001	0.004	0.010
[13.0 , 13.5 [	0.003	0.005	0.002	0.002	0.001	0.001	0.002	0.004	0.001	0.001	0.003	0.014
[13.5 , 14.0 [	0.002	0.001	0.001	0.001	0.001	0.001	0.002	0.002	0.001	0.001	0.002	0.007
[14.0 , 14.5 [	0.001	0.001	0.001	0.003	0.002	0.001	0.001	0.001	0.001	0.003	0.001	0.004
[14.5 , 15.0 [	0.001	0.001	0.001	0.001	0.001	0.001	0.001	0.001	0.001	0.001	0.001	0.002
[15.0 , 15.5 [	0.001	0.001	0.002	0.001	0.001	0.001	0.001	0.001	0.001	0.001	0.001	0.003
[15.5 , 16.0 [	0.001	0.001	0.001	0.001	0.001	0.001	0.001	0.001	0.001	0.001	0.001	0.001
[16.0 , 16.5 [	0.001	0.001	0.001	0.001	0.001	0.001	0.001	0.001	0.001	0.001	0.001	0.001

Supplementary Table S5: Spring constants of the sdENM (6/9)

d	I-K	I-L	I-M	I-N	I-P	I-Q	I-R	I-S	I-T	I-V	I-W	I-Y
[0.0 , 4.0 [	3.559	3.780	4.167	5.025	3.716	4.126	2.546	8.452	3.631	4.159	3.770	5.298
[4.0 , 4.5 [	2.089	4.833	5.404	5.510	3.539	5.757	4.302	3.202	3.366	8.691	3.007	6.810
[4.5 , 5.0 [	5.396	8.617	2.999	6.965	5.176	5.404	4.584	3.789	3.919	6.972	5.797	6.024
[5.0 , 5.5 [	8.847	6.270	8.550	8.759	2.943	10.214	8.718	8.133	8.279	8.356	6.224	4.516
[5.5 , 6.0 [	2.814	3.433	2.033	1.832	2.102	3.147	2.154	2.302	3.172	3.366	5.907	3.982
[6.0 , 6.5 [	1.236	2.461	1.248	1.401	1.228	1.522	1.301	1.910	2.034	2.572	1.889	1.678
[6.5 , 7.0 [	1.395	1.214	1.890	1.212	1.576	1.275	0.690	0.673	1.828	2.635	1.166	1.533
[7.0 , 7.5 [	0.165	0.379	0.215	0.074	0.325	0.143	0.096	0.065	0.093	0.253	0.095	0.349
[7.5 , 8.0 [	0.069	0.095	0.029	0.034	0.074	0.123	0.053	0.036	0.064	0.140	0.040	0.067
[8.0 , 8.5 [	0.042	0.103	0.067	0.019	0.060	0.047	0.027	0.025	0.063	0.117	0.571	0.084
[8.5 , 9.0 [	0.079	0.166	0.066	0.041	0.036	0.072	0.044	0.053	0.069	0.115	0.135	0.083
[9.0 , 9.5 [	0.053	0.104	0.061	0.041	0.035	0.027	0.022	0.022	0.039	0.176	0.089	0.062
[9.5 , 10.0 [	0.049	0.113	0.067	0.061	0.054	0.024	0.057	0.058	0.081	0.103	0.115	0.077
[10.0 , 10.5 [	0.031	0.063	0.054	0.031	0.050	0.037	0.018	0.038	0.038	0.115	0.143	0.106
[10.5 , 11.0 [	0.029	0.037	0.027	0.035	0.013	0.015	0.028	0.011	0.026	0.034	0.196	0.041
[11.0 , 11.5 [	0.025	0.022	0.024	0.011	0.008	0.019	0.011	0.010	0.031	0.050	0.025	0.017
[11.5 , 12.0 [	0.011	0.019	0.010	0.019	0.018	0.008	0.010	0.007	0.008	0.020	0.025	0.017
[12.0 , 12.5 [	0.010	0.012	0.014	0.013	0.007	0.006	0.006	0.009	0.017	0.014	0.018	0.014
[12.5 , 13.0 [	0.008	0.011	0.007	0.005	0.004	0.007	0.006	0.004	0.007	0.016	0.005	0.009
[13.0 , 13.5 [	0.007	0.008	0.004	0.006	0.008	0.003	0.004	0.003	0.004	0.008	0.010	0.009
[13.5 , 14.0 [	0.003	0.006	0.003	0.003	0.004	0.003	0.003	0.002	0.005	0.005	0.004	0.006
[14.0 , 14.5 [	0.002	0.004	0.004	0.003	0.002	0.004	0.003	0.001	0.003	0.003	0.009	0.004
[14.5 , 15.0 [	0.001	0.002	0.002	0.002	0.001	0.002	0.001	0.001	0.001	0.002	0.001	0.003
[15.0 , 15.5 [	0.001	0.002	0.002	0.002	0.001	0.001	0.001	0.001	0.001	0.002	0.002	0.002
[15.5 , 16.0 [	0.001	0.001	0.002	0.001	0.001	0.001	0.001	0.001	0.001	0.001	0.001	0.001
[16.0 , 16.5 [	0.001	0.001	0.001	0.001	0.001	0.001	0.001	0.001	0.001	0.001	0.002	0.002

d	K-K	K-L	K-M	K-N	K-P	K-Q	K-R	K-S	K-T	K-V	K-W	K-Y
[0.0 , 4.0 [	3.167	3.693	3.909	4.613	3.940	3.834	0.883	3.661	4.283	5.477	3.440	5.971
[4.0 , 4.5 [	5.894	5.508	1.664	2.696	1.794	2.521	2.831	2.251	2.937	2.539	3.154	2.522
[4.5 , 5.0 [	1.985	4.461	6.985	2.515	1.686	4.613	1.286	2.420	2.520	3.903	4.817	2.908
[5.0 , 5.5 [	5.587	7.274	7.276	8.116	3.426	4.873	7.243	4.323	6.008	7.322	5.704	7.702
[5.5 , 6.0 [	1.988	1.826	1.980	1.405	1.002	1.232	1.278	1.587	1.836	2.667	1.993	2.006
[6.0 , 6.5 [	0.587	0.946	1.669	0.448	1.257	0.854	0.663	0.666	0.801	1.217	0.711	1.230
[6.5 , 7.0 [	2.507	0.775	0.411	1.304	0.803	0.428	0.279	0.466	0.648	1.116	0.797	0.677
[7.0 , 7.5 [	0.034	0.074	0.084	0.053	0.061	0.043	0.070	0.013	0.042	0.060	0.091	0.077
[7.5 , 8.0 [	0.011	0.051	0.032	0.032	0.015	0.021	0.019	0.008	0.020	0.030	0.052	0.039
[8.0 , 8.5 [	0.031	0.032	0.022	0.026	0.018	0.015	0.019	0.011	0.013	0.029	0.034	0.021
[8.5 , 9.0 [	0.048	0.059	0.038	0.043	0.037	0.012	0.032	0.018	0.033	0.042	0.122	0.082
[9.0 , 9.5 [	0.022	0.038	0.017	0.023	0.029	0.018	0.015	0.017	0.029	0.033	0.062	0.032
[9.5 , 10.0 [	0.045	0.049	0.032	0.017	0.047	0.027	0.021	0.014	0.037	0.044	0.058	0.045
[10.0 , 10.5 [	0.028	0.047	0.025	0.017	0.017	0.013	0.012	0.011	0.009	0.037	0.067	0.060
[10.5 , 11.0 [	0.016	0.022	0.018	0.012	0.006	0.008	0.009	0.005	0.012	0.022	0.034	0.020
[11.0 , 11.5 [	0.008	0.022	0.019	0.012	0.015	0.007	0.004	0.004	0.010	0.018	0.027	0.014
[11.5 , 12.0 [	0.009	0.009	0.010	0.006	0.004	0.003	0.004	0.003	0.006	0.007	0.018	0.012
[12.0 , 12.5 [	0.006	0.008	0.005	0.004	0.006	0.004	0.006	0.003	0.003	0.005	0.010	0.006
[12.5 , 13.0 [	0.004	0.005	0.003	0.003	0.002	0.003	0.002	0.002	0.003	0.006	0.003	0.004
[13.0 , 13.5 [	0.004	0.004	0.003	0.002	0.004	0.002	0.003	0.002	0.002	0.004	0.002	0.003
[13.5 , 14.0 [	0.002	0.003	0.002	0.003	0.002	0.002	0.002	0.001	0.002	0.003	0.002	0.003
[14.0 , 14.5 [	0.002	0.002	0.001	0.002	0.001	0.001	0.001	0.001	0.001	0.002	0.002	0.003
[14.5 , 15.0 [	0.001	0.001	0.001	0.001	0.001	0.001	0.001	0.001	0.001	0.001	0.002	0.001
[15.0 , 15.5 [	0.001	0.002	0.001	0.002	0.001	0.001	0.001	0.001	0.001	0.001	0.002	0.002
[15.5 , 16.0 [	0.001	0.001	0.001	0.001	0.001	0.001	0.001	0.001	0.001	0.001	0.001	0.001
[16.0 , 16.5 [	0.001	0.001	0.002	0.001	0.001	0.001	0.001	0.001	0.001	0.001	0.001	0.001

Supplementary Table S5: Spring constants of the sdENM (7/9)

d	L-L	L-M	L-N	L-P	L-Q	L-R	L-S	L-T	L-V	L-W	L-Y	M-M
[0.0 , 4.0 [	8.150	3.863	4.035	5.453	0.875	7.130	5.034	7.488	3.693	2.099	4.659	5.075
[4.0 , 4.5 [	7.200	5.782	5.413	4.274	1.636	1.262	3.447	3.324	6.012	6.904	3.686	3.225
[4.5 , 5.0 [	5.206	5.535	3.148	3.240	5.384	3.309	2.013	4.383	6.740	3.879	5.555	1.524
[5.0 , 5.5 [	6.226	6.860	6.129	4.451	5.940	6.600	6.473	6.954	6.224	6.609	4.694	1.945
[5.5 , 6.0 [	2.228	1.685	1.842	1.584	2.493	2.135	2.041	2.492	3.493	1.838	2.330	0.888
[6.0 , 6.5 [	1.412	0.749	0.991	1.781	0.825	0.837	0.960	1.322	2.355	1.672	1.697	0.699
[6.5 , 7.0 [	1.215	0.987	0.627	0.751	0.604	0.624	0.482	1.455	1.392	0.896	1.630	1.024
[7.0 , 7.5 [	0.161	0.061	0.070	0.086	0.055	0.058	0.055	0.164	0.180	0.250	0.090	0.118
[7.5 , 8.0 [	0.139	0.061	0.028	0.061	0.021	0.037	0.034	0.047	0.098	0.181	0.063	0.057
[8.0 , 8.5 [	0.128	0.094	0.031	0.027	0.018	0.029	0.018	0.032	0.078	0.060	0.150	0.009
[8.5 , 9.0 [	0.086	0.089	0.056	0.055	0.042	0.041	0.037	0.069	0.129	0.179	0.111	0.157
[9.0 , 9.5 [	0.062	0.046	0.053	0.040	0.020	0.029	0.015	0.035	0.057	0.042	0.058	0.060
[9.5 , 10.0 [	0.080	0.030	0.028	0.027	0.045	0.044	0.023	0.035	0.071	0.086	0.123	0.032
[10.0 , 10.5 [	0.069	0.044	0.038	0.018	0.040	0.042	0.016	0.027	0.056	0.068	0.093	0.061
[10.5 , 11.0 [	0.032	0.029	0.016	0.014	0.016	0.017	0.009	0.020	0.033	0.051	0.039	0.011
[11.0 , 11.5 [	0.023	0.015	0.011	0.008	0.009	0.010	0.008	0.011	0.021	0.013	0.028	0.011
[11.5 , 12.0 [	0.012	0.011	0.008	0.005	0.009	0.008	0.007	0.009	0.025	0.021	0.024	0.007
[12.0 , 12.5 [	0.011	0.010	0.008	0.004	0.006	0.006	0.003	0.008	0.010	0.009	0.010	0.009
[12.5 , 13.0 [	0.010	0.003	0.005	0.002	0.005	0.004	0.003	0.005	0.007	0.007	0.006	0.008
[13.0 , 13.5 [	0.006	0.005	0.003	0.003	0.003	0.004	0.002	0.004	0.006	0.004	0.005	0.003
[13.5 , 14.0 [	0.004	0.003	0.002	0.002	0.002	0.002	0.002	0.003	0.005	0.003	0.004	0.001
[14.0 , 14.5 [	0.002	0.002	0.002	0.001	0.001	0.002	0.001	0.002	0.003	0.003	0.005	0.001
[14.5 , 15.0 [	0.002	0.001	0.001	0.001	0.001	0.001	0.001	0.002	0.002	0.003	0.002	0.003
[15.0 , 15.5 [	0.001	0.001	0.001	0.001	0.002	0.001	0.001	0.001	0.002	0.001	0.002	0.003
[15.5 , 16.0 [	0.001	0.001	0.001	0.001	0.001	0.001	0.001	0.001	0.001	0.001	0.001	0.001
[16.0 , 16.5 [	0.001	0.001	0.001	0.001	0.001	0.001	0.001	0.001	0.001	0.001	0.001	0.001

d	M-N	M-P	M-Q	M-R	M-S	M-T	M-V	M-W	M-Y	N-N	N-P	N-Q
[0.0 , 4.0 [	3.320	4.192	3.320	4.001	3.625	4.264	4.343	4.405	4.072	3.932	3.765	4.524
[4.0 , 4.5 [	9.984	1.452	1.552	1.923	3.027	2.133	2.447	0.639	6.685	4.138	1.945	2.314
[4.5 , 5.0 [	4.149	2.187	2.678	2.874	1.030	1.994	4.130	3.268	3.950	1.151	2.130	2.874
[5.0 , 5.5 [	6.263	2.972	5.891	5.568	6.329	6.088	6.909	6.033	4.590	6.275	2.750	6.722
[5.5 , 6.0 [	2.030	1.078	2.916	2.518	1.381	2.044	1.975	3.158	2.703	2.338	0.685	1.171
[6.0 , 6.5 [	0.364	0.992	0.486	0.637	0.331	0.614	2.296	0.663	1.069	1.675	0.672	0.591
[6.5 , 7.0 [	0.844	0.460	0.853	0.231	0.872	0.282	0.540	2.934	0.932	0.840	2.221	0.883
[7.0 , 7.5 [	0.026	0.032	0.053	0.050	0.030	0.059	0.138	0.087	0.024	0.036	0.032	0.064
[7.5 , 8.0 [	0.009	0.023	0.011	0.023	0.006	0.030	0.059	0.027	0.084	0.079	0.016	0.023
[8.0 , 8.5 [	0.020	0.008	0.069	0.017	0.008	0.015	0.081	0.024	0.026	0.003	0.012	0.014
[8.5 , 9.0 [	0.023	0.038	0.015	0.023	0.026	0.014	0.062	0.053	0.083	0.037	0.028	0.031
[9.0 , 9.5 [	0.017	0.009	0.017	0.020	0.018	0.030	0.054	0.063	0.026	0.018	0.008	0.021
[9.5 , 10.0 [	0.018	0.024	0.018	0.051	0.019	0.049	0.042	0.022	0.103	0.029	0.030	0.023
[10.0 , 10.5 [	0.034	0.009	0.060	0.031	0.011	0.034	0.063	0.025	0.030	0.015	0.011	0.019
[10.5 , 11.0 [	0.013	0.011	0.007	0.007	0.011	0.010	0.026	0.015	0.034	0.015	0.009	0.007
[11.0 , 11.5 [	0.005	0.006	0.005	0.007	0.006	0.006	0.017	0.010	0.011	0.007	0.003	0.003
[11.5 , 12.0 [	0.011	0.005	0.003	0.007	0.003	0.005	0.012	0.006	0.011	0.005	0.004	0.005
[12.0 , 12.5 [	0.007	0.003	0.003	0.003	0.002	0.005	0.009	0.006	0.003	0.004	0.002	0.006
[12.5 , 13.0 [	0.002	0.003	0.002	0.009	0.002	0.002	0.008	0.005	0.011	0.004	0.002	0.003
[13.0 , 13.5 [	0.002	0.004	0.002	0.002	0.001	0.005	0.003	0.003	0.004	0.010	0.001	0.004
[13.5 , 14.0 [	0.001	0.003	0.002	0.001	0.001	0.002	0.002	0.002	0.005	0.004	0.001	0.002
[14.0 , 14.5 [	0.001	0.001	0.001	0.002	0.001	0.001	0.002	0.001	0.002	0.002	0.003	0.001
[14.5 , 15.0 [	0.001	0.001	0.001	0.001	0.001	0.001	0.001	0.001	0.001	0.001	0.001	0.001
[15.0 , 15.5 [	0.001	0.001	0.001	0.001	0.001	0.001	0.001	0.002	0.001	0.001	0.001	0.001
[15.5 , 16.0 [	0.001	0.001	0.001	0.001	0.001	0.001	0.001	0.001	0.001	0.001	0.001	0.001
[16.0 , 16.5 [	0.001	0.001	0.002	0.001	0.001	0.001	0.001	0.001	0.001	0.001	0.001	0.001

Supplementary Table S5: Spring constants of the sdENM (8/9)

d	N-R	N-S	N-T	N-V	N-W	N-Y	P-P	P-Q	P-R	P-S	P-T	P-V
[0.0 , 4.0 [	4.021	2.571	1.378	4.491	3.597	1.789	2.173	3.278	3.019	4.214	4.120	4.460
[4.0 , 4.5 [	5.461	7.215	3.572	4.132	0.796	5.027	3.673	3.914	6.783	2.785	7.003	2.450
[4.5 , 5.0 [	1.922	1.685	2.313	3.095	3.212	5.282	2.272	1.509	1.404	2.064	1.063	4.192
[5.0 , 5.5 [	6.063	5.803	5.734	6.695	4.150	3.166	2.738	3.270	3.723	3.675	5.577	6.181
[5.5 , 6.0 [	1.732	0.969	2.926	1.860	1.334	2.065	0.608	0.989	1.359	0.791	1.591	2.191
[6.0 , 6.5 [	0.341	0.789	1.018	0.643	0.565	0.923	1.384	0.679	1.040	0.630	0.872	1.695
[6.5 , 7.0 [	0.255	0.467	0.857	0.889	0.946	0.455	1.146	0.651	0.355	0.788	0.748	1.187
[7.0 , 7.5 [	0.019	0.045	0.034	0.102	0.098	0.016	0.085	0.082	0.039	0.071	0.047	0.064
[7.5 , 8.0 [	0.026	0.008	0.028	0.034	0.302	0.020	0.048	0.012	0.012	0.017	0.015	0.029
[8.0 , 8.5 [	0.018	0.005	0.011	0.024	0.021	0.034	0.019	0.012	0.051	0.006	0.019	0.038
[8.5 , 9.0 [	0.030	0.019	0.025	0.037	0.021	0.059	0.025	0.023	0.013	0.027	0.018	0.023
[9.0 , 9.5 [	0.011	0.018	0.022	0.027	0.054	0.037	0.045	0.026	0.009	0.015	0.020	0.021
[9.5 , 10.0 [	0.016	0.004	0.019	0.033	0.024	0.038	0.055	0.014	0.017	0.016	0.030	0.025
[10.0 , 10.5 [	0.007	0.020	0.018	0.022	0.014	0.015	0.015	0.010	0.016	0.010	0.020	0.026
[10.5 , 11.0 [	0.006	0.011	0.016	0.014	0.016	0.019	0.008	0.006	0.014	0.004	0.011	0.013
[11.0 , 11.5 [	0.004	0.003	0.012	0.010	0.008	0.017	0.004	0.005	0.005	0.007	0.007	0.008
[11.5 , 12.0 [	0.005	0.003	0.005	0.007	0.006	0.011	0.011	0.002	0.004	0.005	0.004	0.004
[12.0 , 12.5 [	0.004	0.002	0.003	0.006	0.005	0.006	0.005	0.003	0.004	0.004	0.004	0.005
[12.5 , 13.0 [	0.003	0.003	0.002	0.005	0.007	0.005	0.001	0.002	0.001	0.001	0.002	0.004
[13.0 , 13.5 [	0.002	0.002	0.002	0.004	0.011	0.003	0.002	0.001	0.001	0.002	0.002	0.003
[13.5 , 14.0 [	0.001	0.002	0.002	0.003	0.005	0.003	0.002	0.001	0.002	0.001	0.002	0.004
[14.0 , 14.5 [	0.001	0.001	0.001	0.001	0.003	0.002	0.001	0.001	0.001	0.001	0.001	0.002
[14.5 , 15.0 [	0.001	0.001	0.001	0.001	0.001	0.001	0.001	0.001	0.001	0.002	0.001	0.001
[15.0 , 15.5 [	0.001	0.001	0.001	0.001	0.003	0.001	0.001	0.001	0.001	0.001	0.001	0.001
[15.5 , 16.0 [	0.001	0.001	0.001	0.001	0.001	0.001	0.001	0.001	0.001	0.001	0.001	0.001
[16.0 , 16.5 [	0.001	0.001	0.001	0.001	0.001	0.001	0.001	0.001	0.001	0.001	0.001	0.001

d	P-W	P-Y	Q-Q	Q-R	Q-S	Q-T	Q-V	Q-W	Q-Y	R-R	R-S	R-T
[0.0 , 4.0 [	5.658	2.934	3.929	3.108	2.001	4.443	4.480	6.497	4.219	3.884	4.238	5.393
[4.0 , 4.5 [	3.402	2.726	3.528	5.016	2.837	3.966	3.877	3.653	5.466	1.818	2.785	3.298
[4.5 , 5.0 [	3.654	2.723	3.828	2.493	2.761	2.997	4.446	0.766	4.588	3.489	1.748	1.239
[5.0 , 5.5 [	4.970	2.323	7.265	4.665	4.777	5.490	5.753	7.787	4.575	4.835	3.724	4.831
[5.5 , 6.0 [	3.280	1.053	0.865	1.446	1.286	1.368	1.926	2.295	3.462	1.047	0.838	1.002
[6.0 , 6.5 [	2.085	1.424	1.161	0.659	0.498	0.761	1.310	1.809	1.164	1.021	0.486	0.898
[6.5 , 7.0 [	1.498	0.677	0.435	0.531	0.247	0.530	0.786	0.741	0.556	0.361	0.282	0.799
[7.0 , 7.5 [	0.124	0.100	0.017	0.037	0.025	0.028	0.129	0.034	0.029	0.025	0.038	0.023
[7.5 , 8.0 [	0.078	0.047	0.009	0.007	0.015	0.007	0.022	0.016	0.031	0.006	0.009	0.007
[8.0 , 8.5 [	0.035	0.040	0.038	0.018	0.020	0.013	0.034	0.030	0.021	0.022	0.008	0.020
[8.5 , 9.0 [	0.136	0.095	0.016	0.033	0.031	0.018	0.024	0.019	0.051	0.027	0.024	0.019
[9.0 , 9.5 [	0.036	0.045	0.013	0.012	0.008	0.018	0.027	0.059	0.081	0.010	0.008	0.012
[9.5 , 10.0 [	0.036	0.031	0.008	0.015	0.013	0.014	0.050	0.035	0.054	0.014	0.012	0.025
[10.0 , 10.5 [	0.011	0.027	0.019	0.053	0.015	0.012	0.029	0.021	0.028	0.018	0.013	0.021
[10.5 , 11.0 [	0.007	0.015	0.012	0.010	0.004	0.008	0.014	0.020	0.014	0.009	0.006	0.007
[11.0 , 11.5 [	0.005	0.025	0.008	0.007	0.007	0.004	0.009	0.015	0.017	0.006	0.003	0.009
[11.5 , 12.0 [	0.007	0.011	0.003	0.003	0.002	0.003	0.007	0.008	0.011	0.004	0.005	0.004
[12.0 , 12.5 [	0.001	0.009	0.002	0.004	0.002	0.003	0.006	0.007	0.004	0.005	0.003	0.002
[12.5 , 13.0 [	0.005	0.008	0.002	0.004	0.003	0.002	0.006	0.009	0.007	0.002	0.002	0.003
[13.0 , 13.5 [	0.006	0.002	0.002	0.002	0.002	0.002	0.002	0.002	0.004	0.001	0.002	0.002
[13.5 , 14.0 [	0.004	0.002	0.001	0.001	0.001	0.001	0.003	0.002	0.002	0.001	0.001	0.001
[14.0 , 14.5 [	0.002	0.001	0.001	0.001	0.001	0.001	0.002	0.005	0.001	0.001	0.001	0.001
[14.5 , 15.0 [	0.001	0.001	0.001	0.001	0.001	0.001	0.001	0.001	0.001	0.001	0.001	0.002
[15.0 , 15.5 [	0.001	0.001	0.001	0.001	0.001	0.001	0.001	0.001	0.001	0.001	0.001	0.001
[15.5 , 16.0 [	0.001	0.001	0.001	0.001	0.001	0.001	0.001	0.001	0.001	0.001	0.001	0.001
[16.0 , 16.5 [	0.001	0.001	0.001	0.001	0.001	0.001	0.001	0.001	0.001	0.001	0.001	0.001

Supplementary Table S5: Spring constants of the sdENM (9/9)

d	R-V	R-W	R-Y	S-S	S-T	S-V	S-W	S-Y	T-T	T-V	T-W	T-Y
[0.0 , 4.0 [	7.121	6.368	5.130	8.520	5.236	9.399	3.320	4.356	9.553	6.000	3.809	3.723
[4.0 , 4.5 [	2.310	2.668	3.371	5.756	6.379	4.077	3.106	4.673	3.747	4.629	3.298	3.286
[4.5 , 5.0 [	2.628	2.078	4.855	2.606	2.666	3.111	2.896	4.429	1.217	4.131	5.821	1.928
[5.0 , 5.5 [	5.830	7.161	4.993	2.495	5.144	5.366	3.541	6.014	5.168	7.128	6.994	4.748
[5.5 , 6.0 [	1.747	0.778	2.597	0.957	1.246	2.185	1.884	1.548	1.521	2.669	1.597	2.237
[6.0 , 6.5 [	1.571	1.159	0.605	0.785	0.510	0.808	0.730	0.595	1.647	0.957	1.464	1.483
[6.5 , 7.0 [	1.059	0.629	1.439	1.053	0.490	0.603	0.446	0.525	0.695	1.047	0.319	1.268
[7.0 , 7.5 [	0.066	0.156	0.018	0.017	0.039	0.061	0.103	0.013	0.303	0.088	0.149	0.127
[7.5 , 8.0 [	0.023	0.022	0.011	0.004	0.011	0.031	0.025	0.021	0.013	0.035	0.035	0.039
[8.0 , 8.5 [	0.030	0.017	0.024	0.003	0.026	0.033	0.015	0.016	0.013	0.033	0.004	0.047
[8.5 , 9.0 [	0.038	0.082	0.065	0.013	0.019	0.029	0.029	0.025	0.020	0.039	0.058	0.078
[9.0 , 9.5 [	0.026	0.051	0.026	0.030	0.024	0.027	0.008	0.028	0.031	0.029	0.043	0.033
[9.5 , 10.0 [	0.038	0.033	0.044	0.011	0.013	0.028	0.016	0.029	0.032	0.035	0.080	0.034
[10.0 , 10.5 [	0.029	0.036	0.018	0.011	0.014	0.024	0.021	0.050	0.007	0.047	0.026	0.084
[10.5 , 11.0 [	0.021	0.010	0.014	0.002	0.010	0.009	0.005	0.018	0.010	0.027	0.017	0.017
[11.0 , 11.5 [	0.009	0.018	0.016	0.004	0.004	0.007	0.010	0.005	0.007	0.008	0.051	0.010
[11.5 , 12.0 [	0.005	0.006	0.007	0.002	0.004	0.005	0.010	0.012	0.003	0.007	0.012	0.015
[12.0 , 12.5 [	0.006	0.005	0.003	0.001	0.003	0.004	0.005	0.003	0.002	0.005	0.006	0.007
[12.5 , 13.0 [	0.004	0.003	0.004	0.001	0.002	0.003	0.002	0.002	0.003	0.003	0.004	0.004
[13.0 , 13.5 [	0.004	0.003	0.003	0.001	0.001	0.003	0.004	0.003	0.003	0.004	0.005	0.005
[13.5 , 14.0 [	0.002	0.001	0.001	0.001	0.002	0.003	0.001	0.001	0.003	0.003	0.001	0.003
[14.0 , 14.5 [	0.001	0.001	0.002	0.001	0.001	0.001	0.001	0.002	0.001	0.002	0.001	0.003
[14.5 , 15.0 [	0.001	0.001	0.001	0.001	0.001	0.001	0.001	0.001	0.001	0.001	0.001	0.001
[15.0 , 15.5 [	0.001	0.001	0.001	0.001	0.001	0.001	0.002	0.001	0.001	0.001	0.001	0.001
[15.5 , 16.0 [	0.001	0.001	0.001	0.001	0.001	0.001	0.001	0.001	0.001	0.001	0.001	0.001
[16.0 , 16.5 [	0.001	0.001	0.001	0.001	0.001	0.001	0.001	0.001	0.001	0.001	0.001	0.001

d	V-V	V-W	V-Y	W-W	W-Y	Y-Y
[0.0 , 4.0 [	6.749	4.047	5.521	3.320	4.161	4.372
[4.0 , 4.5 [	5.375	3.485	7.551	2.909	4.775	3.364
[4.5 , 5.0 [	3.879	5.118	4.295	3.502	4.355	3.164
[5.0 , 5.5 [	6.795	6.638	8.155	2.977	4.393	5.364
[5.5 , 6.0 [	3.721	3.661	3.538	2.311	3.954	2.809
[6.0 , 6.5 [	2.140	1.446	1.768	0.848	0.585	0.578
[6.5 , 7.0 [	1.768	1.391	1.536	1.229	2.174	1.708
[7.0 , 7.5 [	0.265	0.143	0.168	0.148	0.086	0.188
[7.5 , 8.0 [	0.093	0.056	0.049	0.013	0.037	0.035
[8.0 , 8.5 [	0.074	0.065	0.043	0.022	0.075	0.040
[8.5 , 9.0 [	0.052	0.106	0.056	0.017	0.031	0.054
[9.0 , 9.5 [	0.058	0.086	0.063	0.032	0.093	0.023
[9.5 , 10.0 [	0.052	0.031	0.064	0.142	0.099	0.036
[10.0 , 10.5 [	0.050	0.063	0.084	0.044	0.066	0.043
[10.5 , 11.0 [	0.033	0.073	0.043	0.088	0.059	0.022
[11.0 , 11.5 [	0.023	0.026	0.019	0.013	0.017	0.015
[11.5 , 12.0 [	0.014	0.019	0.010	0.002	0.012	0.021
[12.0 , 12.5 [	0.012	0.015	0.009	0.024	0.014	0.025
[12.5 , 13.0 [	0.009	0.019	0.007	0.006	0.009	0.006
[13.0 , 13.5 [	0.006	0.004	0.004	0.002	0.005	0.004
[13.5 , 14.0 [	0.005	0.003	0.004	0.006	0.002	0.003
[14.0 , 14.5 [	0.002	0.002	0.003	0.002	0.004	0.002
[14.5 , 15.0 [	0.002	0.002	0.001	0.003	0.002	0.002
[15.0 , 15.5 [	0.001	0.002	0.001	0.001	0.001	0.001
[15.5 , 16.0 [	0.001	0.001	0.001	0.001	0.001	0.001
[16.0 , 16.5 [	0.001	0.001	0.001	0.001	0.001	0.001
