# Supplementary material for: Effective Harmonic Potentials: Insights into the Internal Cooperativity and Sequence-Specificity of Protein Dynamics
Source: PLoS Comput Biol. 2013 Aug 29;9(8):e1003209. doi: 10.1371/journal.pcbi.1003209 (PMC3757084; doi:10.1371/journal.pcbi.1003209)
Supplement: Text S1 — Derivation of effective harmonic potentials. (PDF) [file pcbi.1003209.s013.pdf]

Effective harmonic potentials: insights into the internal cooperativity and sequence-specificity of protein dynamics.

Yves Dehouck and Alexander S. Mikhailov

Supporting Text 1: Derivation of effective harmonic potentials

Let us first consider a single protein network, consisting of n residues connected by a certain number of elastic bonds. N_p is the total number of residue pairs, and is equal to $n(n-1)/2$. The total energy of the network, E^{tot}, is constant at a given temperature, and can be expressed as the sum of the energies associated with each bond in the network:

$$E^{\text{tot}} = \sum_{ij}^{N_p} E_{ij}^{\text{bond}}. \tag{S1}$$

In the overdamped limit:

$$E_{ij}^{\text{bond}} = \frac{1}{2}\kappa_{ij}\sigma_{r_{ij}}^2 = k_B T \frac{\kappa_{ij}}{\gamma_{ij}}, \tag{S2}$$

where κ_{ij} is the stiffness of the spring connecting residues i and j. It is equal to zero for residue pairs that are not connected. $\sigma_{r_{ij}}^2$ is the variance of the distance between residues i and j in the equilibrium ensemble, and γ_{ij} is the apparent stiffness of the pair (i, j).

Assuming that relevant experimental data about the equilibrium ensemble of the protein is available, $\sigma_{r_{ij}}^2$ and γ_{ij} can be computed for each pair of residues (i, j). However, this does not allow to directly identify the values of the spring constants κ_{ij} that would ensure an optimal reproduction, by the ENM, of the observed dynamical behavior of the protein. For example, two pairs (a, b) and (c, d) may be connected by elastic bonds of equal stiffness ($\kappa_{ab} = \kappa_{cd}$), but experience differently the influence of the network and thus behave differently, i.e. be characterized by different γ_{ij} values. On the other hand, from any set of spring constants κ'_{ij}, the ENM can be built and employed to compute the apparent stiffness γ'_{ij} of each pair (i, j). The optimal set of spring constants that we wish to determine is the one that leads to predicted values (γ'_{ij}) as close as possible to the corresponding experimental values (γ_{ij}). Valuable information may thus be retrieved from the comparison of γ'_{ij} and γ_{ij} and used to refine an initial, approximately chosen, set of the spring constants κ'_{ij}.

If we focus on a given pair of residues (a, b), we can rewrite eq. **S1** as follows:

$$E^{\text{tot}} = E_{ab}^{\text{bond}} + E_{ab}^{\text{network}}, \tag{S3}$$

where E_{ab}^{network} is simply the sum of the energies associated with all other bonds in the network:

$$E_{ab}^{\text{network}} = \sum_{ij \neq ab}^{N_p} E_{ij}^{\text{bond}} = E^{\text{tot}} - E_{ab}^{\text{bond}}. \tag{S4}$$

If the residues (a, b) are not directly connected by an elastic spring, $E_{ab}^{\text{network}} = E^{\text{tot}}$. On the other hand, if the network consists of only two beads connected by an elastic spring, $E_{ab}^{\text{bond}} = E^{\text{tot}}$. Note that both E^{bond} and E^{network} depend on the choice of a particular pair (a, b), whereas E^{tot} is identical for all pairs. E_{ab}^{network} can thus be understood as the influence that the network as a whole has on the individual pair

(a, b). Indeed, since E^{tot} is constant at a given temperature, the value of E_{ab}^{network} determines E_{ab}^{bond}, and thus the amplitude of the fluctuations of the distance r_{ab}.

If we build the ENM using an approximate set of spring constants κ'_{ij}, and follow the procedures described in the main text of the manuscript (*Methods*), we may compute the covariance matrix of the spatial coordinates $\mathbf{C}$ (eqs. **7, 8**) and, subsequently, the apparent stiffness of each pair of residues (i, j), γ'_{ij} (eqs. **1, 10**). This allows the obtention of an estimation of both E_{ab}^{network} and E_{ab}^{bond}:

$$E'^{\text{network}}_{ab} = \sum_{ij \neq ab}^{N_p} k_B T \frac{\kappa'_{ij}}{\gamma'_{ij}} = E^{\text{tot}} - k_B T \frac{\kappa'_{ab}}{\gamma'_{ab}}, \quad \text{(S5)}$$

$$E'^{\text{bond}}_{ab} = k_B T \frac{\kappa'_{ab}}{\gamma'_{ab}}, \quad \text{(S6)}$$

E'^{network}_{ab} is determined by the overall architecture of the network, and by the spring constants κ'_{ij} assigned to all other bonds, but depends only marginally on the value of κ'_{ab}. Therefore, in view of refining the value of κ'_{ab}, we may consider that E'^{network}_{ab} is a good approximation of E_{ab}^{network}. If we set $E_{ab}^{\text{network}} = E'^{\text{network}}_{ab}$ in eq. **S3**, we find with eqs. **S5** and **S6**:

$$E'^{\text{bond}}_{ab} = E^{\text{bond}}_{ab}, \quad \text{(S7)}$$

and consequently:

$$\kappa_{ab} = \kappa'_{ab} \frac{\gamma_{ab}}{\gamma'_{ab}}. \quad \text{(S8)}$$

The idea is thus that E'^{bond}_{ab} is a relatively good approximation of E^{bond}_{ab}, even if $\kappa'_{ab} \neq \kappa_{ab}$. Indeed, since the contribution of the network is mostly independent of the error on κ'_{ab}, this error should be (mostly) compensated by the resulting error on γ'_{ab}. For example, if the stiffness of the spring connecting residues a and b is underestimated ($\kappa'_{ab} < \kappa_{ab}$), the pair (a, b) should appear as less rigid in the ENM than in the real protein ($\gamma'_{ab} < \gamma_{ab}$).

Eq. **S8** allows to compute a new estimation of κ_{ab}, which is dependent on the overall quality of the initial set of spring constants κ'_{ij}. It is thus necessary to devise an iterative procedure. At each step of this procedure, the values of the apparent stiffness γ'_{ij} predicted using approximate values of the spring constants, κ'_{ij}, are compared with the experimental values γ_{ij}, in order to obtain refined approximations of the spring constants, κ_{ij}, which will be used as κ'_{ij} during the next step. This procedure is expected to converge when $\gamma'_{ij} \rightarrow \gamma_{ij}$, that is, when the predictions of the model agree with the experimental observations.

Other methods have been proposed to identify the optimal value of the spring constant to be assigned to each bond in a given protein, using for example entropy maximization [1]. However, our purpose here is more general, as we wish to derive a set of spring constants $\kappa(s, d)$ that depend only on the nature of the amino acids (s) and on the interresidue distances (d), and that may be applied to any protein. For that purpose, we consider that the optimal value of κ for one given pair (s, d) is the one that leads to the most efficient reproduction of the dynamical behavior of this type of residue pair in a mean protein environment.

By analogy with eq. **S3**, we may write:

$$\overline{E} = \overline{E}^{\text{bond}}(s, d) + \overline{E}^{\text{network}}(s, d) \quad \text{(S9)}$$

$\overline{E}^{\text{bond}}$ is the energy of the elastic spring connecting two residues of type (s, d), in a mean protein environment:

$$\overline{E}^{\text{bond}}(s, d) = \frac{1}{2} \kappa(s, d) \sigma_r^2(s, d) = k_B T \frac{\kappa(s, d)}{\overline{\gamma}(s, d)}. \quad \text{(S10)}$$

where $\kappa(s, d)$ is the value that we want to identify for each type of pairs (s, d), and $\overline{\gamma}(s, d)$ the apparent stiffness extracted from the dataset of 1500 NMR ensembles. $\overline{E}^{\text{network}}(s, d)$ represents the influence of the mean protein environment on pairs of a given type (s, d), and is considered to depend only marginally on the stiffness of the bond between this particular type of pairs (although any estimation of $\overline{E}^{\text{network}}$ will depend

more generally on the set of κ values for all types of pairs). $\overline{E}$ is to the mean protein environment what E^{tot} is to a single protein, and is assumed to be constant and equal for all types of pairs (s, d).

Starting from an approximate functional form of the spring constants, $\kappa'(s, d)$, we can compute an estimation of the apparent stiffness of each type of pairs in a mean protein environment, $\overline{\gamma}'(s, d)$. By analogy with eq. **S8**, we find:

$$\kappa(s, d) = \kappa'(s, d)\frac{\overline{\gamma}(s, d)}{\overline{\gamma}'(s, d)} \tag{S11}$$

which can be used to devise an iterative procedure in which the functional form of $\kappa(s, d)$ is updated at each step k by confronting the predicted values of the apparent stiffness, $\overline{\gamma}_k(s, d)$, with the experimental ones, $\overline{\gamma}(s, d)$:

$$\kappa_{k+1}(s, d) = \kappa_k(s, d)\frac{\overline{\gamma}(s, d)}{\overline{\gamma}_k(s, d)} \tag{S12}$$

The procedure is thus expected to converge when $\overline{\gamma}_k(s, d) \to \overline{\gamma}(s, d)$, that is, when the predictions of the model agree with the experimental observations.

References

[1] Lezon RL, Bahar I (2010) Using entropy maximization to understand the determinants of structural dynamics beyond native contact topology. *PLoS Comput Biol* 6:e1000816.
